# Identification of sense and antisense transcripts regulated by drought in sugarcane

**DOI:** 10.1007/s11103-012-9922-1

**Published:** 2012-05-19

**Authors:** Carolina Gimiliani Lembke, Milton Yutaka Nishiyama, Paloma Mieko Sato, Rodrigo Fandiño de Andrade, Glaucia Mendes Souza

**Affiliations:** Laboratório de Transdução de Sinal, Departamento de Bioquímica, Instituto de Química, Universidade de São Paulo, Av. Prof. Lineu Prestes 748, São Paulo, SP 05508-000 Brazil

**Keywords:** Sugarcane, Drought, Gene expression, Antisense transcriptome

## Abstract

Sugarcane is an important sugar and energy crop that can be used efficiently for biofuels production. The development of sugarcane cultivars tolerant to drought could allow for the expansion of plantations to sub-prime regions. Knowledge on the mechanisms underlying drought responses and its relationship with carbon partition would greatly help to define routes to increase yield. In this work we studied sugarcane responses to drought using a custom designed oligonucleotide array with 21,901 different probes. The oligoarrays were designed to contain probes that detect transcription in both sense and antisense orientation. We validated the results obtained using quantitative real-time PCR (qPCR). A total of 987 genes were differentially expressed in at least one sample of sugarcane plants submitted to drought for 24, 72 and 120 h. Among them, 928 were sense transcripts and 59 were antisense transcripts. Genes related to Carbohydrate Metabolism, RNA Metabolism and Signal Transduction were selected for gene expression validation by qPCR that indicated a validation percentage of 90 %. From the probes presented on the array, 75 % of the sense probes and 11.9 % of the antisense probes have signal above background and can be classified as expressed sequences. Our custom sugarcane oligonucleotide array provides sensitivity and good coverage of sugarcane transcripts for the identification of a representative proportion of natural antisense transcripts (NATs) and sense-antisense transcript pairs (SATs). The antisense transcriptome showed, in most cases, co-expression with respective sense transcripts.

**Electronic supplementary material:**

The online version of this article (doi:10.1007/s11103-012-9922-1) contains supplementary material, which is available to authorized users.

## Introduction

Sugarcane is an important food and bioenergy source and a significant component of the world economy. Sugarcane is cultivated on 24 million hectares which correspond to 0.5 % of the world agricultural area (FAOSTAT 2009). The substitution of gasoline by sugarcane bioethanol has been shown to reduce by 80 % green house gas emissions (Macedo et al. 2008) and many countries have adopted mandates for blending increasing the demand and leading to the expansion of planted areas. Sustainable practices are determining the development of an international bioenergy market. Among other things, it is expected that bioenergy crops are able to grow, be harvested and processed with a low water footprint (Waclawovsky et al. 2010). Water stress is one of the major abiotic stresses affecting the development of plants. The development of sugarcane cultivars tolerant to drought conditions would allow for the expansion of plantations to sub-prime regions. Knowledge on the mechanisms underlying drought responses and its relationship with carbon partition would greatly help to define routes to increase yield.

Water deficit impinges on photosynthesis and on the consequent accumulation of photosynthetic products (Shao et al. 2006). Gene products induced by drought stress are involved in the protection of cells and in the regulation of signal transduction pathways of stress responses. Some of these products are chaperons, late embryogenesis abundant proteins, water channels, sugar transporters, enzymes involved in osmolyte synthesis, transcription factors, protein kinases, protein phosphatases and 14-3-3 proteins (Shao et al. 2006; Shinozaki and Yamaguchi-Shinozaki 2006). Rocha et al. (2007) analyzed the transcriptome of sugarcane plants (cultivar SP90-1638) after 24, 72 and 120 h of water deficit using cDNA microarrays containing 1,545 genes. The group identified 93 genes differentially expressed that included MYB and WRKY transcription factors and low temperature induced proteins.

In order to increase knowledge on the sugarcane transcriptome associated to drought, we hybridized the same samples used by Rocha in custom designed oligonucleotide arrays with 21,901 different probes. The oligoarrays were designed to contain probes that detect transcription in both sense and antisense orientation.

Natural antisense transcripts (NAT) are classified as endogenous RNA molecules that contain sequences complementary to other RNA transcripts (Lapidot and Pilpel 2006) and are divided into two groups: cis-NAT which are formed by sense and antisense transcripts from the same genomic locus and trans-NAT which are formed from sense and antisense transcripts from different loci (Henz et al. 2007).

The importance of sense-antisense transcript pairs (SATs) in the regulation of gene expression is strongly suggested by its evolutionary conservation and shared characteristics between animals and plants (Kiyosawa et al. 2005). The identification of overlapping gene pairs is being favored through genome wide searches in genomes of different species (Jen et al. 2005). In maize, the antisense transcripts represent about 6.5 % for anther and 14.3 % for pollen transcriptome (Ma et al. 2006). In *Arabidopsis*, 3.7 % of all transcripts pairs are cis-NAT pairs (Henz et al. 2007).

Natural antisense transcripts may regulate the expression of their target genes in different levels including transcription, messenger RNA processing and splicing or polyadenylation (Jen et al. 2005), stability, cellular transport, translation (Lapidot and Pilpel 2006) and methylation status of the sense gene (Kiyosawa et al. 2005). An antisense transcript can also be transcribed in one locus and regulate the expression of a different gene (Kiyosawa et al. 2005). Some SATs generate multiple-sized transcripts that are not polyadenylated and tend to localize in the nucleus in both animals and plants (Kiyosawa et al. 2005). The identification of NATs, the different mechanisms of action with gene examples and the regulation of NAT transcription was reviewed by Lapidot and Pilpel (2006).

At present, most commercial DNA microarrays are designed to hybridize mainly to protein coding sense transcripts and disregard most antisense transcripts. The coverage and sensitivity of commercial DNA probe arrays are sufficient for monitoring antisense RNA expression in total RNA on a genome-wide scale (Werner et al. 2007). In the absence of a sugarcane commercial array with enough coverage of the transcriptome and with probes to detect antisense transcripts, we designed a custom oligonucleotide using the Agilent Platform. Agilent custom arrays were already used in the investigation of SATs for mouse (Kiyosawa et al. 2005) and maize transcriptomes (Ma et al. 2006). Antisense transcripts that are regulated by drought have been identified in *Arabidopsis.* Using a GeneChip *Arabidopsis* tiling array, Matsui et al. (2008) identified after 10 h of drought treatment 2,466 SATs. They also observed a linear correlation between treated/untreated expression ratio of sense and antisense transcripts. SATs identified in wheat showed over-representation of transcripts involved in energy production suggesting that antisense transcription may affect the expression of valuable agronomic phenotypes (Coram et al. 2009).

In the current study, we sought to develop a protocol that could be used for large scale gene expression analysis of sugarcane genes and for identification of sense and antisense transcription using a custom Agilent oligonucleotide array. We validated the results obtained using quantitative real-time PCR (qPCR) and discuss the gene categories altered by water deficit.

## Methods

### Agilent array design

**Fig. 1:**
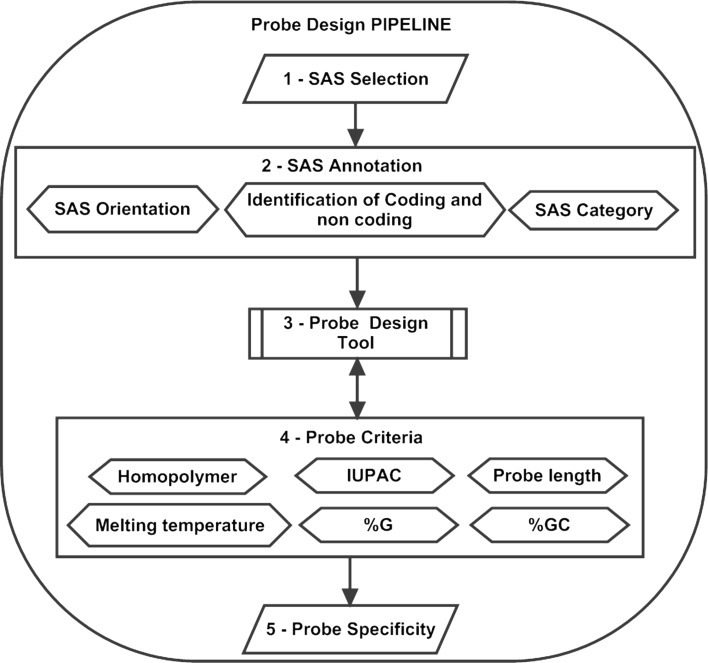
The Probe Design Pipeline was composed of five main steps: *1* Selection of most representative SAS, *2* Identification of Orientation, coding and non-coding SAS and Selection by Functional Category, *3* Design of all available sense and antisense probes for selected SAS, *4* Exclusion of probes that do not agree with the criteria and *5* Probe Blast alignment against Sugarcane EST database to identify the unique probes

This 44 K microarray consists of sense and antisense probes designed from potential genes using an in house pipeline (Fig. 1) based on the 43,141 Sugarcane Assembled Sequences (SAS) from the Sugarcane EST Project (SUCEST) (Vettore et al. 2003). The features are distributed in a 4 × 44 K array format and are composed of 45,220 total features, corresponding to 1,217 Agilent Controls and 43,803 probes representing SAS. The probes are represented in duplicate on the array, so each array has 21,901 different probes.

In the first step of the pipeline we selected the 29,689 most representative SAS in the SUCEST Project, corresponding to 26,303 contigs and 686 singletons. Singletons of interest and that have been studied under the SUCAST and SUCAMET Project (Rocha et al. 2007; Papini-Terzi et al. 2009) were also included.

Since the sugarcane genome complete sequence is not available, we obtained information about exons, introns and SAS orientation by similarity searches against NCBI’s NR protein databank using blastx with an e-value of 10e^−8^. These searches made possible the identification of SAS orientation and to classify them as coding or non-coding. SAS orientation was defined based on the frame from the three first hits in blastx. For non-coding SAS, orientation was defined based on the frequency of EST sequences orientation (5′→3′ or 3′→5′) that composes the SAS. The selected SAS are mainly related to Signal Transduction, Carbohydrate and Cell Wall metabolism, Stress responses and Transcription Factors.

The probe design tool makes an automated search for all available probes for selected SAS and selected the best ones following the probe criteria (Table 1) in the sense and antisense orientation, discarding probes that disagree with one of the parameters mainly to avoid cross-hybridization. It is well documented that probe specificity and sensitivity depend on multiple factors including uniqueness, GC content, steric hindrance, optimal melting temperature and distance from the 3′ end of the ORF (Tsai et al. 2006; Hughes et al. 2001; Nakaya et al. 2007).

**Table 1:** Parameters used to ensure probe sensitivity and specificity

Description	Parameters
Probe length	60 mer
Final orientation	5′→3′
IUPAC	ACTG
% G	<50 %
% GC	≥35 % and ≤ 55 %
Tm	≥68 and ≤ 76
Homopolymer	≤6
Probe location	3′

**Fig. 2:**
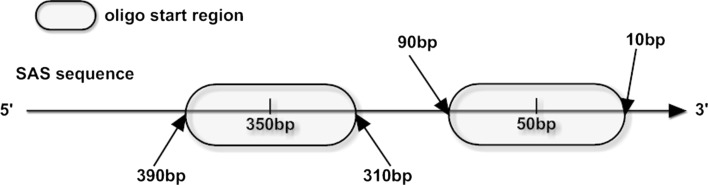
Scheme of oligonucleotide design. Probes start in a distance of 40 bp around positions 50 and 350 bp (*grey square*)

The initial goal was to select two probes in the sense orientation, one at the position 50 bp and another at position 350 bp, and one probe in the antisense orientation located at the position 50 bp for each SAS, always using as start reference the 3′ SAS end. The SAS containing at least one probe sense and one antisense starting in a distance of 40 bp around positions 50 or 350 bp (Fig. 2) were preferred, but all sense probes that fit the criteria were accepted for the specificity analysis (Fig. 1).

A homology search using BLASTN between the selected probes and the Sugarcane ESTs database was used to evaluate specificity and to find alternative targets. The first probe hit should have 100 % identity with the respective transcript and the following hits a Score bit lower than a threshold of 42.1, corresponding to probe coverage lower than 21 bp without gaps (35 % coverage).

### Plant material and cultivation

The plant material used was the same as in Rocha et al. (2007). Plantlets of a sugarcane cultivar SP90-1638 sensitive to drought (Internal Technical Report, CTC, 2002) were obtained from one-eyed seed cultivated on moist sand for 15 days prior to drought experiments. Three biological replicates were performed, two of the replicates were used for microarray experiments and one for qPCR reactions. The plants were transferred to pots containing moist sand, irrigated with Hoagland’s solution (Hoagland and Arnon 1950) and maintained under greenhouse conditions. Regular watering was controlled and maintained for 90 days, being withheld after this period only for the experimental group. To control for water loss, soil samples were collected and the humid weight of each soil sample was compared with its dried weight, in order to verify the water loss in experimental plants. Aerial parts of six plants were collected 24, 72 and 120 h after the onset of drought for the experimental and control groups. This was done in triplicates (three biological replicates) from each experimental point that were immediately frozen in liquid nitrogen (Rocha et al. 2007). Physiological parameters were measured for 10 days as described (Rodrigues et al. 2009).

### RNA extraction, oligoarray hybridization and image processing

Total RNA was extracted as in Rocha et al. (2007). Frozen tissues were grinded using a homogenizer. Tissue samples of 2–2.5 g were weighted and grinded to a fine powder in liquid nitrogen using a pre-cooled mortar and pestle. The pulverized tissue was transferred to a 50 ml tube and homogenized with 5 ml Trizol (Invitrogen) per gram of tissue according to the manufacturer’s instructions. RNA pellets were resuspended in 20 μl of warm diethyl pyrocarbonate-treated water, vortexing gently for about 15 min. RNA samples were quantified in a spectrophotometer and loaded on 1 % agarose/formaldehyde gels for quality inspection. Total RNA was treated with DNase I Amplification Grade enzyme (Invitrogen by Life Technologies) and then purified with RNeasy^®^ Mini Kit (Qiagen) following the RNA Cleanup Kit protocol.

Sample preparation and hybridization was done following the Two-Color Microarray-Based Gene Expression Analysis (Quick Amp Labeling) Protocol. Spike controls were prepared using RNA Spike-In Kit, Two-Color (Agilent Technologies) according to manufacturer’s protocols, and were used in the amplification and labeling reactions. Cyanine 5- and Cyanine 3-labeled and amplified cRNAs were obtained from 2 μg of total RNA from control and experimental samples using the Agilent’s Quick Amp Labeling Kit that uses T7 RNA polymerase, which simultaneously amplifies target material and incorporates Cy3- or Cy5-labeled CTP (Agilent Technologies). Labeled and amplified cRNA was purified using RNeasy mini spin columns from RNeasy^®^ Mini Kit (Qiagen) and quantified using the NanoDrop ND-1000 UV–VIS Spectrophotometer (Thermo Scientific). Hybridization was done following the Gene Expression Hybridization Kit protocol (Agilent Technologies). After 17 h of hybridization, slides were washed with Gene Expression Wash Buffer 1 and Gene Expression Wash Buffer 2 (Agilent Technologies) with 0.005 % Triton X-102 following the Agilent protocol. Slides were scanned using GenePix 4000B scanner (Molecular Devices, Sunnyvale, CA, USA) and Agilent Scan Settings. Two biological replicates and dye swaps were used for each experimental point (Electronic Supplementary Table 1).

### Normalization, data processing and analysis

Two protocols have been implemented to automate the identification of differentially expressed genes, to determine if differential expression was significant or if a signal was significantly above the noise of the background. Feature Extraction 9.5.3.1 software (Agilent Technologies) was used to extract data using as reference the benchmark for Two-Color microarray from Agilent—February 2007 version (protocol GE2-v5_95_Feb07) with minor adjustments. First we applied a background signal correction. The normalization, composed of two steps, was initially applied across the entire range of array data (a linear normalization method). To correct for intensity-dependent dye biases we applied a non-linear LOWESS normalization (Yang et al. 2002) minimizing the variations caused by experimental procedures. Second, outlier genes were identified using a modified HTself method (Vencio and Koide 2005) adapted for the Agilent Platform. The HTSelf method uses only the LOWESS normalization on the log2-ratio. In contrast, the new approach uses two normalization steps and they are applied on each signal separately. We consider a gene model to be up/down regulated if 90 % confidence is obtained for each reference set based on the modified HTSelf method. Additionally we consider a gene to be expressed only if the majority (70 % of all) of the spots shows the same expression profile in one experiment as defined by the HTSelf method. A gene transcript level is defined as enriched in a given condition if the expression level was considered significantly higher in the two biological replicates. This means that the sum of all spots for each gene must have the majority of all spots with the same expression profile. To determine if a feature is significantly above background we developed an analysis based on the significance test of the Feature Extraction software. First, the signal is defined significant if IsWellAboveBG (Is Well Above BackGround) FLAG = 1. This eliminates the signals that were not distinguishable from the local background signal. The criteria assumes that the spot need to have Flag = 1 for IsPosAndSignif (Is Positive And Significant) established via a 2-sided *t* test, which indicates if the mean signal of a spot is greater than the corresponding background and additionally if the gBGSubSignal (Background-subtracted green signal) is greater than 2.6*g(r)BG_SD (green (red) BackGround Standard Deviation). Second, the spot was used and considered a significant signal only if it was positively flagged in the two biological replicates. The last step was the calculation of log2-ratio.

### Functional annotation/categorization

As cited previously, the SUCEST Project generated a total of 43,141 SAS that were estimated to represent a total of 33,600 unique genes. Since initial gene annotation of the SUCEST database was done in 2001 we felt it necessary to produce an updated version. The new annotation and categorization was done automatically and manually, and comprises two principal categories based in gene function and structure. The reference databases used for the alignments were: NCBI-NR (http://www.ncbi.nlm.nih.gov/), Uniprot/Swissprot (http://www.uniprot.org), Gene Ontology (http://www.geneontology.org), KEGG (http://www.genome.jp/kegg/), *Sorghum bicolor* (http://genome.jgi-psf.org/), *Zea mays* (http://genome.jgi-psf.org), *Oryza sativa* (http://www.plantbiology.msu.edu) and *Arabidopsis thaliana* (http://www.arabidopsis.org) species.

Similarity searches were also performed between SAS and sequences available in the DFCI Sugarcane Index (version 3.0) and in NCBI Microarray GEO 09/2009. The sugarcane EST assembly in DFCI Sugarcane Index 3.0 (SGI—http://compbio.dfci.harvard.edu/tgi/cgi-bin/tgi/gimain.pl?gudb=s_officinarum) corresponds to the public reference of sugarcane genes and gives information about the genes, virtual pattern of expression, function and evolutionary relationships. The NCBI Gene Expression Omnibus (GEO) corresponds to an extensive repository of transcriptome data that includes gene expression measurements, sequencing data and others. In this way it was possible to integrate different sources and resources to be used in the functional annotation in order to improve the SAS characterization.

We established thirty different functional categories to manually categorize each SAS based in the information obtained with the web tool cited and based in information obtained from the literature. A secondary and more flexible group of categories was defined based in the gene structure or gene families (Electronic Supplementary Table 2).

To analyze the function enrichment of large gene lists, we used the GeneMerge tool (Castillo-Davis and Hartl 2003), which uses the hypergeometric distribution for obtaining the rank scores for the overrepresentation of the studied gene sets (the differentially expressed genes) compared to the population gene sets (the microarray set of sugarcane genes).

### Quantitative real-time PCR (qPCR)

For gene expression validation of differentially expressed sense oligonucleotides, cDNA synthesis was done using SuperScript First-Strand Synthesis System for RT-PCR (Invitrogen by Life Technologies) and random hexamers and oligo(dT) primers as described in Rocha et al. (2007). For gene expression validation of the differentially expressed sense and antisense oligonucleotides pairs, strand-specific reverse transcription was done using SuperScript III First-Strand Synthesis Super Mix (Invitrogen by Life Technologies) following manufacture’s protocol and gene specific primer (GSP) for the amplification of sense or antisense transcripts. Total RNA used as template for reverse transcriptase reactions was initially treated with DNase I Amplification Grade enzyme (Invitrogen by Life Technologies). An aliquot of treated RNA was used in qPCR to rule out DNA contamination. For the GSP design, we analyzed the SAS sequence correspondent to the oligonucleotide differentially expressed. At first, we defined the orientation of the SAS sequence using blastx against the NR-NCBI database. For amplification of the sense transcript we designed a reverse primer starting near the 3` end of the SAS sequence. For amplification of the antisense transcript we designed a forward primer starting near the 5` end of SAS sequence. For amplification of endogenous reference gene we used a reverse GSP primer for glyceraldehyde 3-phosphate dehydrogenase (GAPDH) Gene ID: 542367 (Papini-Terzi et al. 2009). Primers were designed using Primer 3 (http://frodo.wi.mit.edu/primer3/) and the following parameters: 68 °C ≤ Tm ≤ 72 °C and 40–60 % GC. Primers for qPCR were designed using Primer Express 2.0 Software (Applied Biosystems by Life Technologies) and the following parameters: 58 °C ≤ Tm ≤ 60 °C Tm, 30–80 % GC content and 50–150 bp amplicon length. For validation of SATs, qPCR primers were designed in a sequence with approximately 300 bp around the oligonucleotide sequence position. Primer specificity was confirmed by blastn at the SUCEST database. Primer sequences are shown in ESM_6. All qPCR reactions were done in triplicates. As SYBR Green PCR Master Mix (Applied Biosystems by Life Technologies) was used in the reactions, dissociation curves were done to evaluate for the presence of contaminants. PCR amplification was monitored and analyzed with 7300 Real Time PCR System (Applied Biosystems by Life Technologies). Primer efficiencies were calculated in standard curve dilutions and primers with efficiency below 90 % and greater than 110 % were excluded from analyses. Expression ratio was determined by 2^−DDCt^ method (Livak and Schmittgen 2001) and statistical significance as described in Rocha et al. (2007).

## Results

### Array probes

An in house pipeline was developed for the creation of customized arrays. After processing and filtering putative probes, we obtained 21,902 unique probes with high specificity (21,901 probes in duplicate and 1 single probe, resulting in 43,803 probes in the customized array). From the 21,901 probes, 14,554 probes were selected from the SUCEST database, 10,417 probes were designed to hybridize to sense transcripts and 4,137 probes were designed to hybridize to antisense transcripts. From the 7,347 probes selected from the SUCAST/SUCAMET database, there are 3,243 sense and 3,243 antisense probes close to position 50 bp and 861 sense probes close to position 350 bp. The 43,803 probes present in the array represent 14,522 different SAS.

### Differentially expressed genes

**Fig. 3:**
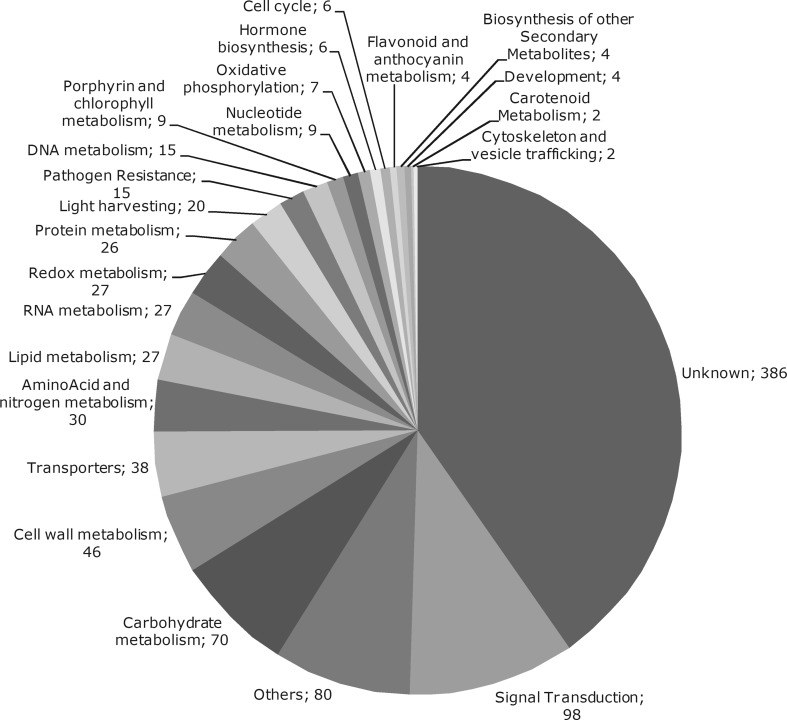
Functional categories of genes differentially expressed in sugarcane plants submitted to drought stress for 24, 72 and 120 h. Numbers indicate the total of genes identified in each category

A total of 987 probes were differentially expressed in at least one sample of sugarcane plants submitted to drought for 24, 72 and 120 h (Electronic Supplementary Table 3). Among them, 928 were sense transcripts and 59 were antisense. Only 24 differentially expressed genes had both sense and antisense transcripts regulated by drought and 22 of them had the same expression pattern between antisense and sense, meaning, when sense transcript was up-regulated, the antisense transcript was also up-regulated and vice versa. As seen before (Rocha et al. 2007), the number of differentially expressed genes increased significantly after 72 and 120 h of water stress compared to 24 h of stress. From the thirty functional categories created, only two categories were not represented (Fig. 3). Using the GeneMerge Tool, we could identify functional categories enriched in each experimental time point (Table 2).

**Table 2:** Enriched functional categories after water withholding

24 h		72 h		120 h	
E-score	Description	E-score	Description	E-score	Description
1.94E−09	Unknown	2.98E−126	Unknown	5.92E−171	Unknown
7.34E−08	Signal transduction	1.22E−19	RNA metabolism	2.05E−55	Signal transduction
0.000643	Transporters	2.68E−19	Signal transduction	1.13E−33	Others
0.016202	DNA metabolism	1.38E−15	Others	6.52E−23	Carbohydrate metabolism
0.096266	Protein metabolism	7.33E−14	Carbohydrate metabolism	4.90E−20	Light harvesting
		2.11E−10	Cell wall metabolism	9.96E−18	Amino acid and nitrogen metabolism
		4.20E−08	Protein metabolism	9.59Ev16	Transporters
		2.14E−07	Amino acid and nitrogen metabolism	9.97E−16	Lipid metabolism
		4.66E−07	Redox metabolism	8.19E−12	Pathogen resistance
		1.87E−06	DNA metabolism	4.70E−10	Cell wall metabolism
		3.57E−06	Porphyrin and chlorophyll metabolism	4.24E−08	Redox metabolism
		0.000455	Lipid metabolism	3.81E−06	Oxidative phosphorylation
		0.000593	Nucleotide metabolism	1.07E−05	Protein metabolism
		0.000593	Pathogen resistance	3.55E−05	Hormone biosynthesis
		0.001092	Transporters	6.52E−05	DNA metabolism
		0.001851	Light harvesting	0.001815	Biosynthesis of other secondary metabolites
		0.013756	Flavonoid and anthocyanin metabolism	0.003273	RNA metabolism
		0.013756	Biosynthesis of other secondary metabolites	0.003692	Cell cycle
		0.013756	Development	0.012429	Porphyrin and chlorophyll metabolism
		0.131435	Cell cycle	0.049646	Development
		0.325692	Hormone biosynthesis	0.052671	Nucleotide metabolism
				0.058446	Carotenoid metabolism
				0.771816	Flavonoid and anthocyanin metabolism

### Quantitative real-time PCR (qPCR) validation

Genes were selected for gene expression validation by qPCR. Twenty experimental points (including control and stress samples) from genes where sense transcripts were differentially expressed (exclusively) were tested and eighteen points exhibited the same expression pattern as the oligoarray experiments, resulting in a validation percentage of 90 % (Electronic Supplementary Table 4). For genes with sense and antisense transcripts differentially expressed, thirty-six experimental points (control and stress samples) were tested and all of them exhibited the same expression pattern as observed in the array experiments resulting in 100 % of validation (Electronic Supplementary Table 5).

Genes primarily related to Carbohydrate metabolism, RNA metabolism and Signal Transduction were selected for qPCR validation.

**Fig. 4:**
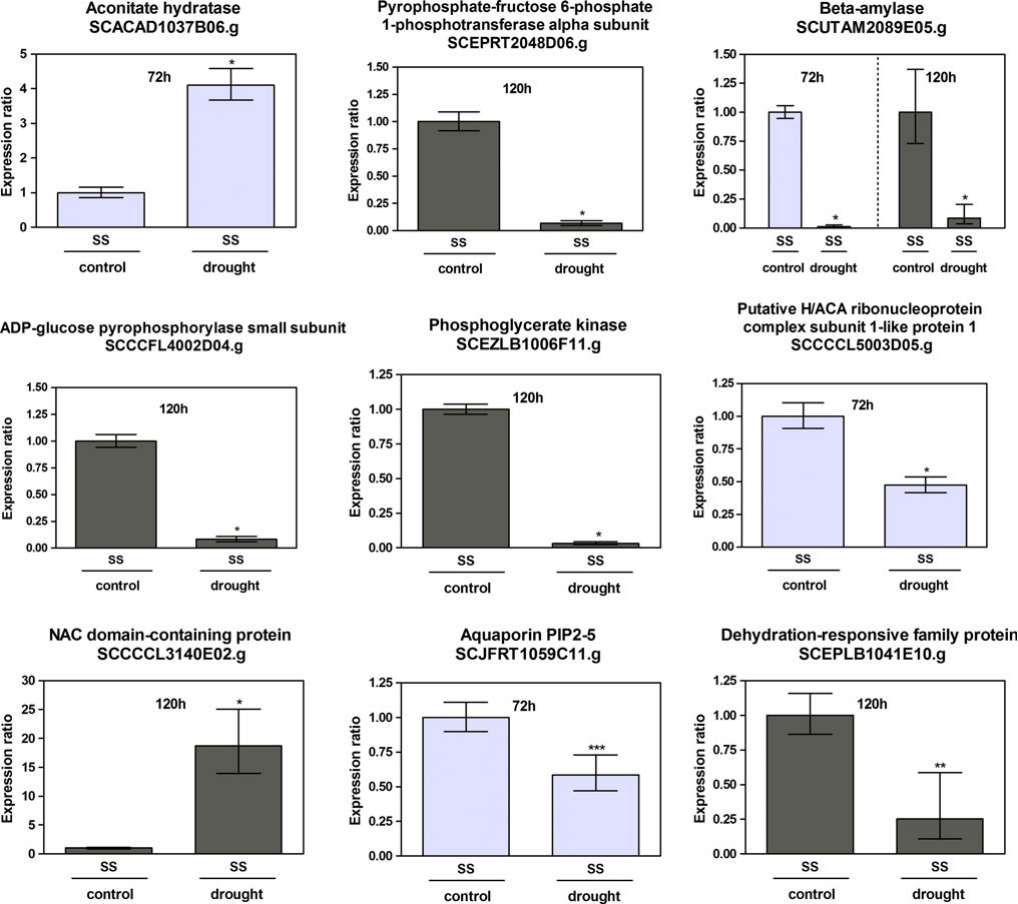
qPCR of sense transcripts regulated by drought stress. The *y* axis is the normalized relative expression ratio between stressed versus irrigated samples. qPCR reactions were done only for experimental points differentially expressed in microarray experiments. Reactions were done in triplicates and on a third biological replicate. *Error bars* were calculated as in Rocha et al. (2007). **p = 0.95; ***p = 0.99; *p = 1.00 for control versus drought sample

It is known that some of the pathways associated with sucrose content may overlap with drought stress signaling pathways (Papini-Terzi et al. 2009). This work shows that different aspects of carbohydrate metabolism were down regulated in drought stress. We observed that the Pyrophosphate-fructose 6-phosphate 1-phosphotransferase alpha subunit (SCEPRT2048D06.g) is repressed after 120 h of drought (Fig. 4). This alpha subunit is involved in the regulation of the enzyme which catalyzes the reversible interconversion of fructose-6-phosphate and fructose-1,6-bisphosphate, a key step in the regulation of the metabolic flux toward glycolysis or gluconeogenesis (Buchanan et al. 2002). A phosphoglycerate kinase (SCEZLB1006F11.g), which catalyzes the formation of 3-phosphoglycerate from 1,3-bisphosphoglycerate in glycolysis is also repressed after 120 h of drought (Fig. 4). An Aconitate hydratase (SCACAD1037B06.g), overexpressed after 72 h of drought (Fig. 4), was the only carbohydrate metabolism gene that we selected for qPCR analysis that was overexpressed; the majority of genes involved in carbohydrate metabolism were down-regulated. The formation and mobilization of starch may also be altered in sugarcane leaves without irrigation. An ADP-glucose pyrophosphorylase small subunit (SCCCFL4002D04.g) involved in the biosynthesis of alpha 1,4-glucans (glycogen or starch) in bacteria and plants was repressed after 120 h of water deprivation (Fig. 4). It was already observed that the large subunit of ADP-glucose pyrophosphorylase was repressed in RNA from epidermal fragments of potato leaves after potato plants had been submitted to water deprivation (Kopka et al. 1997). A beta-amylase (SCUTAM2089E05.g) involved in the cleavage of maltose residues from the non-reducing end of starch was repressed after 72 and 120 h and of drought stress (Fig. 4).

**Fig. 5:**
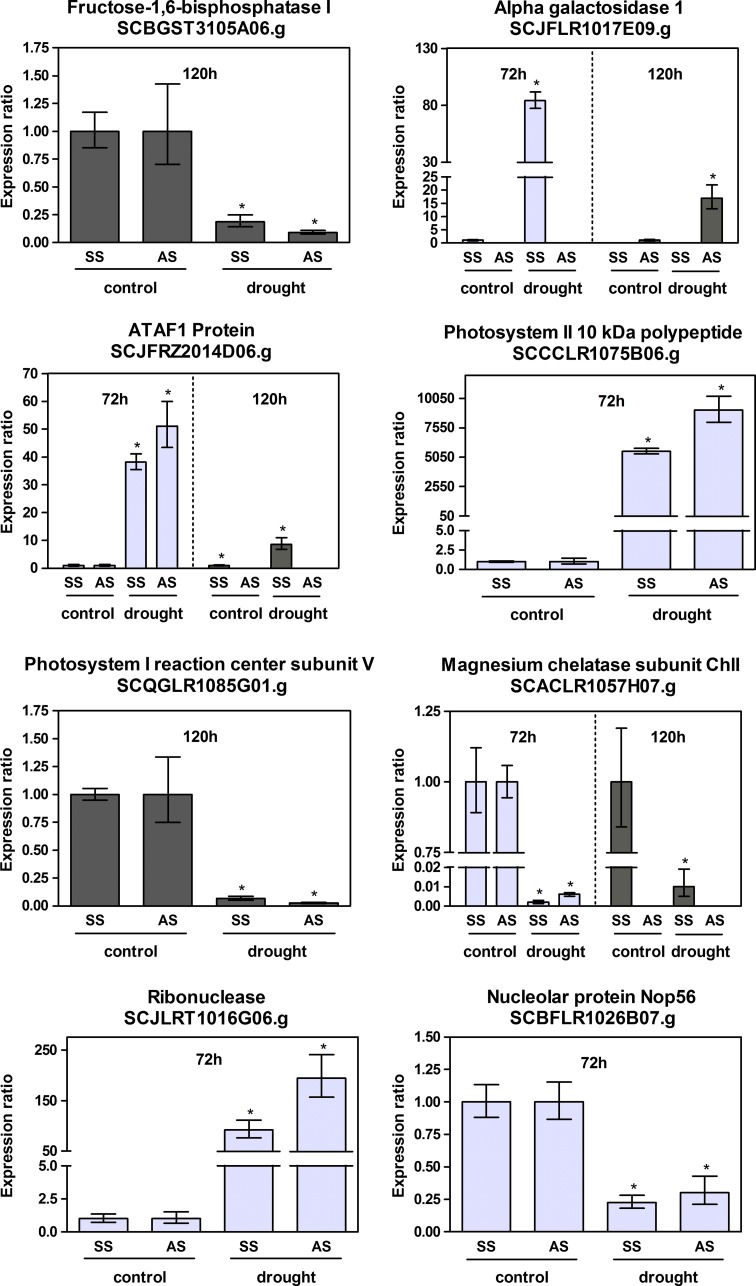
qPCR of sense and antisense transcripts regulated by drought stress. The *y* axis is the normalized relative expression ratio between stressed versus irrigated samples. qPCR reactions were done only for experimental points differentially expressed in microarray experiments. Reactions were done in triplicates; on a third biological replicate and using strand specific cDNA as template. *Error bars* were calculated as in Rocha et al. (2007). *p = 1.00 for control versus drought sample

The expression of genes involved in Photosynthesis was also altered in our experiments. Photosystem I reaction center subunit V was repressed in both sense and antisense transcripts and Photosystem II polypeptide was induced (Fig. 5).

Genes related to RNA metabolism were identified as differentially expressed. Small nucleolar RNAs (snoRNAs) participates in nucleolytic processing of rRNAs, post-transcriptional synthesis of 2′-O-methylated nucleotides and pseudouridines in rRNAs, small nuclear RNAs (snRNAs) and probably other cellular RNAs (Kiss 2002). The snoRNAs are divided into two classes. One class contains the box C/D motifs and directs the 2′-O methylation of rRNA and the other class contains box H and ACA elements and directs the isomerization of uridine to pseudouridine (Kiss 2002). Two RNA binding proteins, one that binds with box C/D snoRNA and one that binds with box H/ACA snoRNA were selected for qPCR validation. A Putative H/ACA ribonucleoprotein complex subunit 1-like protein 1 (SCCCCL5003D05.g) that functions in ribosome biogenesis, pre-mRNA splicing and telomere maintenance (Meier 2005) was repressed after 72 h of water withholding (Fig. 4). The ACA box family of snoRNAs was identified in 1996. At that time, Balakin et al. (1996), observed that all members of the Yeast ACA family were associated with proteins. The H hairpin elements in ACA snoRNAs was identified in the next year (Ganot et al. 1997). Nop56 is one of the proteins that binds in the box C/D core motif (Kiss 2002). Our work shows that a nucleolar protein Nop56 (SCBFLR1026B07.g) was repressed as was the Putative H/ACA ribonucleoprotein complex subunit 1-like cited earlier after 72 h of drought stress, but in this case both sense and antisense transcripts were repressed (Fig. 5). Another gene involved in RNA metabolism, a Ribonuclease (SCJLRT1016G06.g) was up-regulated after 72 h of drought stress in both sense and antisense transcripts (Fig. 5). This gene expression induction was 194.5-fold higher in the antisense transcript and 92.5-fold higher in the sense transcript. The ribonuclease (SCJLRT1016G06.g) has 84 % of identity with an S-like RNase from *Triticum aestivum*. S-RNase is also involved in self-incompatibility, phosphate starvation and inhibition of fungi hyphae development in plants (Goldraij et al. 2006; Qin et al. 2006; Cruz-Garcia et al. 2003; Kock et al. 2006; Hugot et al. 2002).

The NAC transcription factor is well known as being part of drought signaling pathways. We identified that the sense/antisense transcript pair for ATAF1, a NAC domain transcription factor (SCJFRZ2014D06.g) was up-regulated after 72 h and the sense transcript was up-regulated after 120 h of drought. After 72 h of stress, the sense transcript induction was 38-fold and antisense induction was 51-fold. After 120 h of stress the induction was smaller, eightfold for sense transcripts (Fig. 5). Another NAC domain containing protein (SCCCCL3140E02.g) had only the sense transcripts up-regulated after 120 h of stress (Fig. 4). A NAC domain containing transcription factor was already identified as a target for miRNAs in switchgrass, a model biofuel plant species (Matts et al. 2010).

One plasma membrane intrinsic aquaporin (PIP2-5) (SCJFRT1059C11.g) was selected for gene expression validation. Aquaporins are proteins involved in the control of water movement between cells and cell compartments (Maurel and Chrispeels 2001). Papini-Terzi et al. (2009) believe that low expression of aquaporins has been segregated and selected by the breeding process and that this is strongly associated with high sucrose content. Among the abiotic stresses tested by Jang et al. (2004) in *Arabidopsis thaliana* that included drought, cold, high salinity or abscisic acid (ABA) treatment, drought stress was the one that most significantly altered the expression of PIPs. Some of them were up-regulated whereas some were down-regulated. The transcript level of PIP2-5 increased up to fivefold in both the roots and aerial parts. By microarray experiments, we observed that aquaporin PIP2-5 (SCJFRT1059C11.g) was up-regulated after 72 h of drought stress in sugarcane. But this result was not confirmed by qPCR. This method was repeated two times and we concluded that PIP2-5 (SCJFRT1059C11.g) was down-regulated after 72 h of drought stress in sugarcane plants (Fig. 4). Transgenic plants overexpressing PIP2-5 showed rapid water loss during dehydration stress resulting in retarded germination and seedling growth (Jang et al. 2007).

A sugarcane Dehydrin (SCQGLR1085F11.g) that presents 78 % of identity with Sorghum dehydrin DHN1 was evaluated in this work. This type of dehydrin helps in the maintenance of membrane structures in cellular dehydration conditions (Koag et al. 2003; Rorat 2006) and is induced in rice plants submitted to cold, drought and in transgenic plants expressing the CBF1/DREB1b gene under the control of a ubiquitin promoter (Lee et al. 2004b). Dehydrins are proteins that accumulate during late embryogenesis or in response to low temperatures, ABA treatment or any other environment stimuli that causes dehydration, like salinity, drought or freezing (Close 1997). Sense transcripts from the sugarcane dehydrin (SCQGLR1085F11.g) were up-regulated after 72 and 120 h of water deprivation (ESM_3).

Sometimes it is difficult to distinguish between cause and consequence of expression changes in abiotic stress responses. The expression of some genes may be altered as a consequence of the stress. Senescence occurs as an age dependent process and as biotic and abiotic stress-responses. The senescence process involves highly regulated and orderly molecular and cellular events that allow efficient recycling of the nutrients to other sink tissues (Lee et al. 2004a). We detected an alkaline alpha galactosidase 1 (SCJFLR1017E09.g) that was up-regulated in both sense and antisense transcripts after 72 or 120 h of drought stress (Fig. 5). The expression induction of this gene may be a consequence of the stress as Lee et al. (2004a) observed in rice. An alkaline alpha galactosidase (Osh69) was induced during natural leaf senescence and H_2_O_2_ stresses and wounding. Osh69 is involved in the degradation of chloroplast galactolipids during leaf senescence.

### Transcript expression

**Fig. 6:**
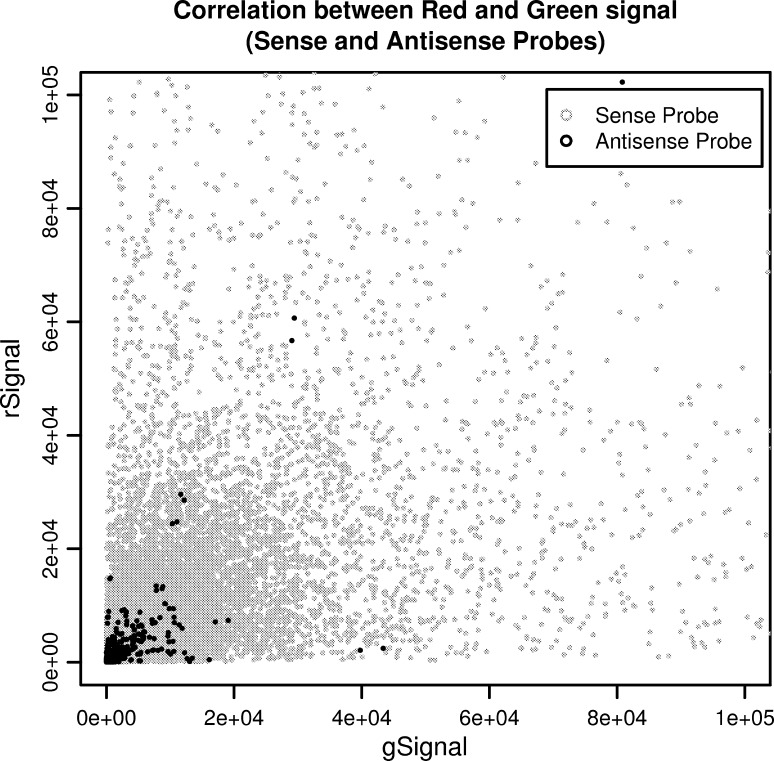
A plot of the *red* background-corrected signal versus the *green* background-corrected signal for sense and antisense features

**Fig. 7:**
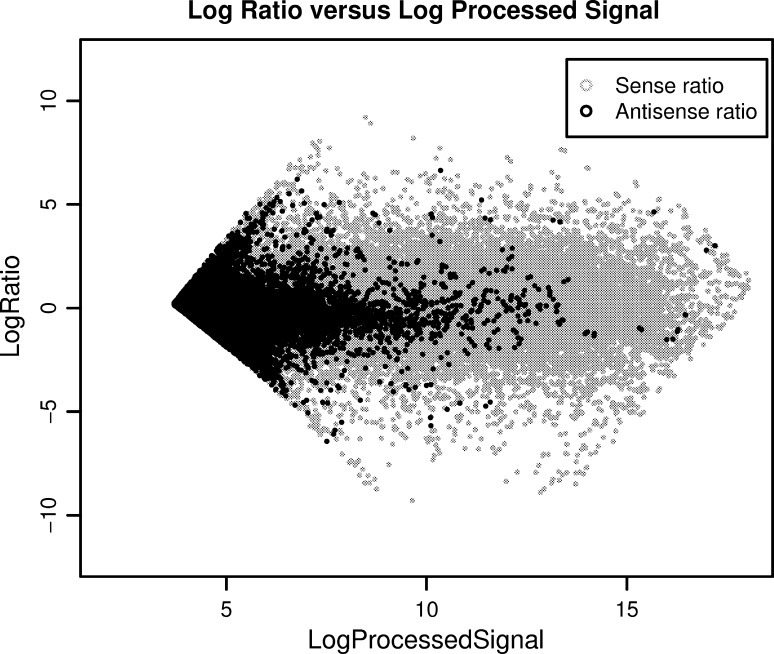
Log ratio of sense and antisense features vs. the log of their *red* and *green* processed signals. The *gray color* represents the sense features and the *black color* represents the antisense features, showing the low log ratio signal for antisense probes

The intensity-based analysis has identified a large number of significantly expressed transcript probes, especially for antisense probes. The low number of differentially expressed antisense probes may be due to their low signal intensity (Figs. 6, 7), as it is known that antisense expression in generally represented in low levels (Verjovski-Almeida et al. 2007; Chan et al. 2006). This fact led us to analyze the signal intensities in the two channels normalized and used separately. This approach has increased the reproducibility and sensitivity for the identification of expressed genes in two independent datasets (Hoen et al. 2004; Bossers et al. 2010).

In the intensity-based analysis we identified 11,780 probes with signal above background in at least one of the six experimental points and 7,973 probes with signal above background in all experimental samples (24 h control and experimental sample, 72 h control and experimental sample and 120 h control and experimental sample). The array has 21,902 different probes of which 14,522 probes hybridize with sense transcripts and 7,380 hybridize with antisense transcripts. From the 11,780 probe signals identified above background, 10,903 were for probes that detect sense transcripts and 876 corresponded to probes that detect antisense transcripts. In this way, 75 % of the sense probes and 11.9 % of the antisense probes that are present on the array have signal above background and can be classified as expressed sequences (Table 3).

**Table 3:** Probes presenting signal above background

	Total on slide	Above background	%
Sense probes	14,522	10,904	75.0
Antisense probes	7,380	876	11.9

The total number of probe signals above background changes in the time course of the experiment. The control samples were irrigated and were collected in parallel with the experimental samples. Over time, there was a decrease in expression of transcripts corresponding to sense probes (above background in control samples). An interesting finding is that the number of antisense probes above background is the same at 24 h on control and drought samples, a little higher in drought samples at 72 h against control plants and it is fourfold higher in drought samples at 120 h compared to control samples (Table 4).

**Table 4:** Probes with signal above background in each experimental point

Sample	SS	AS
24 h control	10,030	609
24 h drought	10,110	609
72 h control	9,745	470
72 h drought	9,689	503
120 h control	8,814	286
120 h drought	7,611	833

**Fig. 8:**
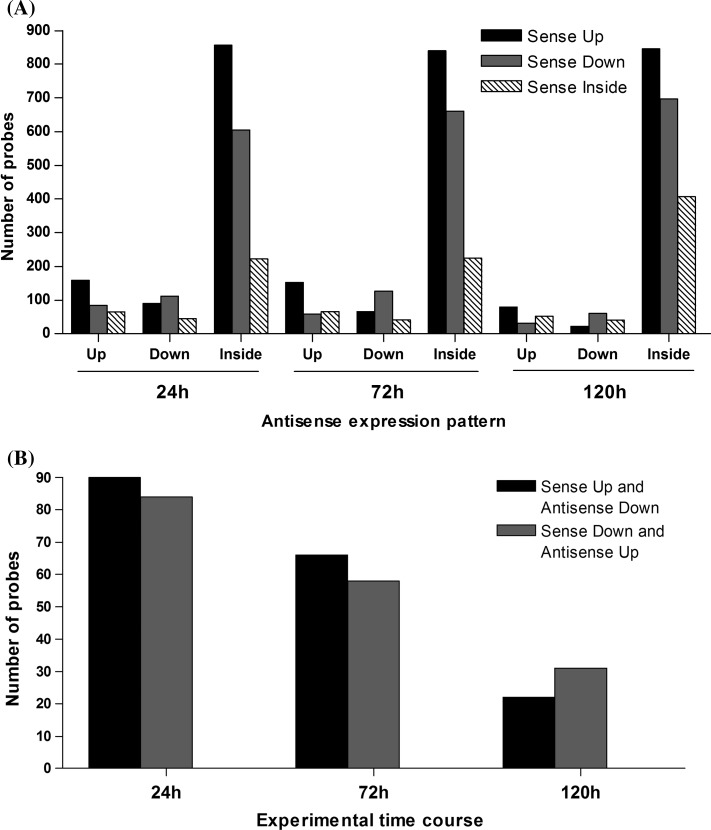
Expression pattern of sense and antisense probe pairs with signal above background. **a** Expression pattern of sense and antisense probes pairs separated by time of water withholding. The *Y* axis indicates the number of probes. The *X* axis indicates the expression pattern of antisense probes. The *colors* on the legend indicate the expression pattern of the sense probe from the probe pair. **b** Expression pattern of sense and antisense probes pairs with opposite expression pattern in each experimental time course

To compare expression profile between sense and antisense transcripts, we selected only oligonucleotides that are represented by both sense and antisense probe pairs in the array. Using the signal intensity log ratio between experimental and control sample, we classified the probe as up-regulated if logratio > 0,down-regulated if logratio < 0 and as inside if the signal intensity is not significantly above the background, based on the filtering background methodology. At first, we could observe that the expression pattern of sense and antisense pairs is quite similar in the three different experimental time points. As cited earlier, there are more sense transcripts above background than antisense transcripts. This can be observed in Fig. 8 where the majority of sense up and sense down transcripts has its antisense pair classified as inside (black and gray bars on the third (antisense inside) group of each experiment time group). The following observations are relative only to pairs which sense and antisense probes were classified as up or down-regulated. When the sense probe is up-regulated (black bars) its respective antisense probe is in most cases also up (first black bar of each time group) (Fig. 8a) and when sense probe is down (gray bars), its respective antisense is in most cases also down (gray bar on the second group of each time group) (Fig. 8a). There are also some examples which sense and antisense have different expression patterns (Fig. 8b) and it is interesting that the increase of drought stress period is accompanied with a decrease in the proportion of pairs of sense and antisense with different expression patterns (Fig. 8b).

**Fig. 9:**
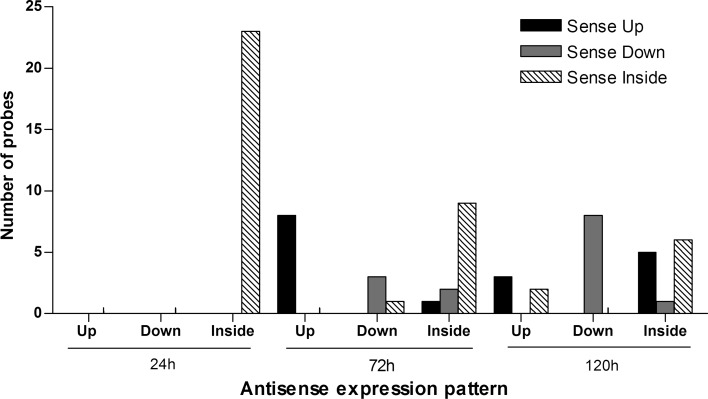
Expression pattern of sense and antisense probe pairs that were identified as differentially expressed by the modified HTself method. The *Y* axis indicates the number of probes. The *X* axis indicates the expression pattern of the antisense probe. The *colors* on the legend indicate the expression pattern of the sense probe from the probe pair

When we analyzed the expression pattern between sense and antisense pairs that were identified as differentially expressed by the modified HTself method, we could not observe the pairs with opposite pattern between sense and antisense transcripts (sense up and antisense down, and/or sense down and antisense up) and at 24 h of water withholding all of the pairs had both probes classified as inside (Fig. 9). This may be due to the high stringency used in the Outlier method.

## Discussion

We have developed a customized oligonucleotide array containing almost 50 % of sugarcane genes. The identification of sense and antisense differential expression will allow for a broader view of gene expression regulation. We have used two different methods in the identification of expressed sense and antisense transcripts. The modified HTSelf method has allowed for the identification of differentially expressed genes with medium to high intensity in relation to a reference, but it has been inefficient to identify sense or antisense probes with low signal intensity. In this way the use of an intensity-based analysis has been more efficient than ratio-based analysis, increasing the number of expressed antisense signals identified. The intensity-based analysis allows the comparison between samples that were not hybridized against each other based in a same common reference. As reported previously, the intensity-based models are very powerful in the analysis of dual-color gene expression data (Hoen et al. 2004; Bossers et al. 2010).

As mentioned earlier, we used the same samples analyzed by Rocha et al. (2007) to evaluate the transcriptome related to drought responses. In this work we used a microarray with 14 times more elements represented. Rocha and colleagues identified 93 genes differentially expressed. Of these, 51 are present on our custom Agilent microarray and 31 genes had the same expression profile in both plataforms. The 20 remaining genes were defined as not differentially expressed in the oligoarrays plataform probably due to increased stringency in the analysis. Rocha et al. (2007) confirmed the expression profile of 4 genes with qPCR. Three of these genes were also identified with the same expression profile in our experiments and the other one was classified as not differentially expressed. Overall the present work is more comprehensive and in good agreement with the previous data.

Drought responses of sugarcane plants are very broad. The expression of genes in twenty-eight functional categories was altered (Fig. 3). The observation of enriched functional categories in each experimental point (24, 72 and 120 h) shows a progression of events during the stress. At the early stage, after 24 h of water withholding, there were fewer genes differentially expressed and an enrichment of expression from genes related to Signal Transduction, Transporters, DNA Metabolism and Protein Metabolism (Table 2). At this point it appears the plant perceives the stress signal and starts to modify basic cell mechanisms such as DNA and Protein metabolism to respond to the stress. After 72 h of water withholding, an array of categories were enriched that may reflect the modification of plant metabolism, including Redox Metabolism, Cell Wall Metabolism and Carbohydrate Metabolism including Photosynthesis. After 120 h of stress, RNA and DNA metabolism, initially enriched at early steps of cell adaptation, are no more on the top list. Signal Transduction is still enriched probably to maintain the stress response on. In this level of stress, different energy pathways are altered (Light harvesting, Carbohydrate Metabolism, Oxidative Phosphorylation and Lipid Metabolism).

Sense and antisense transcripts for a NAC transcription factor were induced by drought stress (SCJFRZ2014D06.g). This gene family is responsive to drought in different species such as *Arabidopsis thaliana,* chickpea and rice (Lu et al. 2007; Peng et al. 2009; Fang et al. 2008). There is evidence suggesting that ATAF1 (a NAC transcription factor), acting as a transcriptional regulator, negatively regulates the expression of stress responsive genes under drought stress in *Arabidopsis thaliana* (Lu et al. 2007). To our knowledge this is the first evidence of antisense expression for this gene pointing to a complex regulation of this pathway.

RNA metabolism is largely altered during water stress in sugarcane plants. An H/ACA ribonucleoprotein complex subunit 1-like protein 1 (SCCCCL5003D05.g) was repressed after water stress. H/ACA RNP are protein-RNA complexes responsible for the most abundant post-transcriptional RNA modification, pseudouridylation. snoRNAs in the ACA family play a direct role in pseudouridine synthesis including site selection (Ni et al. 1997). This reduction in Putative H/ACA ribonucleoprotein complex subunit 1-like protein 1 may lead to reduction in RNA pseudouridylation and a consequent modification in RNA maturation. Nop56p, another RNA binding protein was repressed after water stress (SCBFLR1026B07.g), in complex with Nop1p, is required for ribosome assembly in yeast (Gautier et al. 1997). We identified a ribonuclease (SCJLRT1016G06.g) induced after drought. The induction of a ribonuclease after water stress was already observed by Lewis Dove in 1967 in tomato leaflets (Dove 1967) and in a proteomic study with rice plants (Salekdeh et al. 2002). In a study with barley (*Hordeum vulgare*), a very close relationship between RNase activity and water saturation deficit was found (Arad et al. 1973). It is still to be determined if the increase in ribonuclease expression is a consequence of water deprivation or a mechanism to improve drought tolerance.

Altered expression was observed for a Pyrophosphate-fructose 6-phosphate 1-phosphotransferase alpha subunit (SCEPRT2048D06.g) (down-regulated), phosphoglycerate kinase (SCEZLB1006F11.g) (down-regulated) and an aconitate hydratase (SCACAD1037B06.g) (up-regulated). Transgenic sugarcane clones with reduced cytosolic pyrophosphate: d-fructose-6-phosphate 1-phosphotransferase (PFP) activity displayed significant changes in metabolite levels and fluxes during internode development. In three independent transgenic lines, sucrose concentrations increased between three and sixfold in immature internodes (van der Merwe et al. 2010; Groenewald and Botha 2008). The alpha subunit, as stated earlier, is involved in enzyme regulation because it binds to fructose 2-6-bisphosphate, the enzyme activator. We can hypothesize that the reduction of the alpha subunit expression probably will decrease the binding of the enzyme activator and in consequence, glycolysis will be reduced. Transgenic *Arabidopsis* plants overexpressing PFP displayed increased PFP activity, faster growth but the levels of metabolites appeared not to have significantly changed. Transgenic *Arabidopsis* with reduction expression of PFP showed reduced PFP activity and retarded growth accompanied by reduced rates of CO_2_ assimilation. Opposite to what has been shown for sugarcane, reduced expression of PFP caused a slight decrease in the sucrose levels (Lim et al. 2009). Aconitate hydratase isomerizes citrate to isocitrate in the Citric Acid cycle. The repression of Aconitate hydratase 1 transcripts in wild tomato leads to the increase of CO_2_ assimilation ratio and photosynthetic sucrose synthesis (Carrari et al. 2003). We can hypothesize that the up-regulation of this gene in response to drought may contribute to decrease photosynthesis in sugarcane.

The decrease in the number of transcripts expressed above background during the time course of the experiment and the increase in the number of genes differentially expressed after 72 and 120 h of stress may be explained because the methodology used for the identification of genes differentially expressed does not take into account if the signal of the probe was above or below the background. The observation that at 120 h of drought stress there are fourfold more antisense transcripts above background and that the majority of genes differentially expressed were identified at 120 h of stress, indicates that extended stress causes major alteration in the regulation of gene expression.

In our study with sugarcane aerial parts, 11.9 % of the antisense probes that are present on the array indicated expression of the antisense message. This value is in agreement with values detected for maize leaves. In juvenile leaf, Ma et al. (2006) observed that 10.0–11.1 % of the transcriptome is represented by antisense transcripts. In maize, a lower proportion of antisense transcripts was observed in anther (6.5–6.7 %) and a higher proportion in pollen (14.3 %).

Our data of genes differentially expressed showed a predominance of SATs with the same expression pattern (when sense transcript was up-regulated, the antisense transcript was also up-regulated and vice versa). Henz et al. (2007) concluded with a study using *Arabidopsis* that the simple presence of an antisense transcript is not sufficient for the negative cross regulation. They suggest that the effectiveness of posttranscriptional RNA regulation by RNA interference greatly varies. Depending on the mechanism of antisense action, different relationships between sense and antisense mRNA levels can be expected. In the mechanism of transcriptional interference, two RNA polymerase II complexes on opposite DNA strands might collide with each other and this can cause transcriptional arrest or transcription in only one direction. In this case it is expected an inverse mRNA level between sense and antisense transcripts. In the double-stranded RNA dependent mechanism the presence of both transcripts is required for duplex formation (Lapidot and Pilpel 2006). One explanation for the co-expression of sense and antisense is that the transcription of sense transcripts would reduce nucleosome density throughout the transcribed region, thereby increasing DNA accessibility and the likelihood of non-specific transcription (Struhl 2007). Antisense transcripts might regulate the sense partner in a condition dependent manner. Continuous production of low levels of non-coding antisense transcripts maybe the cost of this regulatory mechanism (Swiezewski et al. 2009). Matsui et al. (2008) studied the biogenesis mechanisms of stress or ABA-inducible antisense RNAs in *Arabidopsis*. They assume two mechanisms for the biogenesis of antisense RNAs. One mechanism is the generation of antisense transcripts by RNA-dependent RNA polymerases from RNA templates. The other mechanism is the generation from DNA template. Combined with chromatin immunoprecipitation studies of the sugarcane genome, the identification of antisense messages might point to putative regulatory elements at 3′ end of genes.

SATs tend to be poly(A) negative in both plants and animals (Kiyosawa et al. 2005). The relation between RNA transcription and RNA processing, like polyadenylation, implies possible different cellular fates. Common labeling methods as the one that we used for this work’s array hybridizations, select for polyadenylated transcripts. This bias may be advantageous to distinguish between transcripts of different functions since fully processed RNA are more stable and thus less likely to be transcriptional noise (Werner et al. 2007).

We conclude that our custom sugarcane oligonucleotide array provides sensitivity and good coverage of sugarcane transcripts for the identification of a representative proportion of NATs and SATs. The antisense transcriptome showed, in most cases, co-expression with respective sense transcripts.

## Additional material

### ID for platform description in GEO

GPL14862 SUCEST-FUN Sugarcane 44 k v1.0 Nov 01, 2012

### ID for sample description in GEO

GSM830125 Sugarcane transcriptome—ch2: Drought_24 h_Exp_1; ch1: control_24 h_1 Nov 01, 2012

GSM830126 Sugarcane transcriptome—ch1: Drought_24 h_Exp_2; ch2: control_24 h_2 Nov 01, 2012

GSM830127 Sugarcane transcriptome—ch2: Drought_72 h_Exp_1; ch1: control_72 h_1 Nov 01, 2012

GSM830128 Sugarcane transcriptome—ch1: Drought_72 h_Exp_2; ch2: control_72 h_2 Nov 01, 2012

GSM830129 Sugarcane transcriptome—ch2: Drought_120 h_Exp_1; ch1: control_120 h_1 Nov 01, 2012

GSM830130 Sugarcane transcriptome—ch1: Drought_120 h_Exp_2; ch2: control_120 h_2 Nov 01, 2012

ID for Series description in GEO GSE33574 Sugarcane Expression Data from Stress Time Series Nov 01, 2012

## Electronic supplementary material

Below is the link to the electronic supplementary material.

**Electronic Supplementary Table** 1 **-** Scheme of microarray hybridizations. (PDF 7 kb)

**Electronic Supplementary Table 2 –** Functional categories for gene annotation. (PDF 15 kb)

**Electronic Supplementary Table 3 –** Genes differentially expressed in sugarcane plants submitted to drought for 24 h, 72 h and 120 h. (PDF 323 kb)

**Electronic Suplementary Table 4 –** qPCR validation of differentially expressed sense transcripts. (PDF 66 kb)

**Electronic Suplementary Table 5 -** qPCR validation of differentially expressed sense and antisense transcripts. (PDF 67 kb)

**Electronic Supplementary Table 6 –** Primer sequences for qPCR validation of differentially expressed transcripts. (PDF 7 kb)
