# Supplementary material for: Identification of sense and antisense transcripts regulated by drought in sugarcane
Source: Plant Mol Biol. 2012 May 19;79(4):461–77. doi: 10.1007/s11103-012-9922-1 (PMC3369129; doi:10.1007/s11103-012-9922-1)
Supplement: Supplementary file 1 — Electronic Supplementary Table 1 - Scheme of microarray hybridizations. (PDF 7 kb) [file 11103_2012_9922_MOESM1_ESM.pdf]

Supplementary Table 1 - Scheme of microarray hybridizations.

	Cy 3		Cy 5
24h	Contr. R1	vs.	Exp. R1
	Exp. R2	vs.	Contr. R2
72h	Contr. R1	vs.	Exp. R1
	Exp. R2	vs.	Contr. R2
120h	Contr. R1	vs.	Exp. R1
	Exp. R2	vs.	Contr. R2

Cy3 and Cy5: dyes. Contr.: control irrigated sample. Exp.: experimental drought sample. R1: biological replicate 1. R2: biological replicate 2
