# Supplementary material for: Identification of sense and antisense transcripts regulated by drought in sugarcane
Source: Plant Mol Biol. 2012 May 19;79(4):461–77. doi: 10.1007/s11103-012-9922-1 (PMC3369129; doi:10.1007/s11103-012-9922-1)
Supplement: Supplementary file 2 — Electronic Supplementary Table 2 – Functional categories for gene annotation. (PDF 15 kb) [file 11103_2012_9922_MOESM2_ESM.pdf]

Functional Category	Examples of Included Processes
Carbohydrate metabolism	Glycolysis
	Krebs Cycle
	Gluconeogenesis
	Pentose Phosphate pathway
	Synthesis and degradation of starch
	Fructose/Mannose/Galactose
	Calvin Cycle
	Inositol metabolism
Light Harvesting	Photochemical reaction centers (PSI e PSII)
Oxidative phosphorylation	
Lipid metabolism	Fatty acid biosynthesis
	Fatty acid elongation in mitochondria
	Fatty acid metabolism
	Synthesis and degradation of ketone bodies
	Steroid hormone biosynthesis
	Glycerolipid metabolism
	Glycerophospholipid metabolism
	Ether lipid metabolism
	Sphingolipid metabolism
	Arachidonic acid metabolism
	Alpha-Linolenic acid metabolism
	Biosynthesis of unsaturated fatty acids
Nucleotide metabolism	Purine metabolism
	Pyrimidine metabolism
AminoAcid and nitrogen metabolism	Alanine, aspartate and glutamate metabolism
	Glycine, serine and threonine metabolism
	Cysteine and methionine metabolism
	Valine, leucine and isoleucine degradation
	Valine, leucine and isoleucine biosynthesis
	Lysine biosynthesis
	Lysine degradation
	Arginine and proline metabolism
	Histidine metabolism
	Tyrosine metabolism
	Phenylalanine metabolism
	Tryptophan metabolism
	Phenylalanine, tyrosine and tryptophan biosynthesis
	beta-Alanine metabolism
	Taurine and hypotaurine metabolism
	Selenoamino acid metabolism
	Cyanoamino acid metabolism

	Glutathione metabolism	
	Nitrogen assimilation pathway	
Cell wall metabolism	N-Glycan biosynthesis	
	Glycosylphosphatidylinositol(GPI)-anchor biosynthesis	
	Glycosphingolipid biosynthesis - globo series	
	Glycosphingolipid biosynthesis - ganglio series	
	Other glycan degradation	
	Lignin metabolism	
	Suberin metabolism	
	Phenylpropanoid biosynthesis	
	Structural proteins of the cell wall	
	Cell wall growth/extension	
	Cell wall differentiation	
Vitamin and Cofactor metabolism	Thiamine metabolism	
	Riboflavin metabolism	
	Vitamin B6 metabolism	
	Nicotinate and nicotinamide metabolism	
	Pantothenate and CoA biosynthesis	
	Biotin metabolism	
	Lipoic acid metabolism	
	Folate biosynthesis	
	One carbon pool by folate	
	Retinol metabolism	
	Ubiquinone and other terpenoid-quinone biosynthesis	
Phorphyrin and chlorophyll metabolism		
Hormone biosynthesis	Auxin synthesis and degradation	Tryptophane metabolism
	ABA synthesis and degradation	Carotenoid byosynthesis
	Ethylene synthesis and degradation	
	Cytokinin synthesis and degradation	Zeatin biosynthesis
	Brassinosteroid synthesis and degradation	Brassinosteroid biosynthesis
	Gibberellin synthesis and degradation	Diterpenoid biosynthesis
	Salicylic acid synthesis and degradation	
	Methyl jasmonate	
Carotenoid Metabolism		
Other terpenoids metabolism	Terpenoid backbone biosynthesis	
	Monoterpenoid biosynthesis	
	Sesquiterpenoid biosynthesis	
	Limonene and pinene degradation	
	Polyketide sugar unit biosynthesis	
Flavonoid and anthocyanin metabolism	Isoflavonoid biosynthesis	
	Anthocyanin biosynthesis	
	Flavonoid biosynthesis	

Biosynthesis of other Secondary Metabolites	Stilbenoid, diarylheptanoid and gingerol biosynthesis	
	Flavone and flavonol biosynthesis	
	Indole alkaloid biosynthesis	
	Isoquinoline alkaloid biosynthesis	
	Tropane, piperidine and pyridine alkaloid biosynthesis	
	Acridone alkaloid biosynthesis	
	Caffeine metabolism	
	Betalain biosynthesis	
	Glucosinolate biosynthesis	
	Benzoxazinoid biosynthesis	
Sulphur metabolism		
DNA metabolism	Replication/repair	
	Histones and associated proteins	
	Metilation and acetylation	
	Telomer maintenance	
RNA metabolism	Transcription	
	RNA binding proteins	
Protein Metabolism	Translation	
	Chaperones	
	Degradation	
	Modification	
Signal Transduction	Biotic stress signal transduction	ABA
		Drought
		Cold
		Light
		Heat
	Abiotic stress signal transduction	Jasmonic acid
		Salicylates
	Internal signal transduction	Auxin
		Cytokinin
		Ethylene
		Brassinosteroid
	Signal transduction - general	Some Kinases and phosohates
		Some Transcription Factors
		Calcium signaling
Cell cycle	Meiosis	
	Mitosis	
	Cell cycle control	
Circadian Clock		
Development	Differentiation	
	Speciation	

Transporters	Simporters
	Antiporters
	Aquaporin
	Membrane transporters
	Pumps
	Channels
	Carriers
Senescence and cell death	
Cytoskeleton and vesicle trafficking	Secretory pathways
	Endocytic pathway
	Protein sorting
Pathogen Resistance	
Redox Metabolism	
Transposable element-related	Transposase
	Reverse transcriptase
Others	
Unknown	
