# Supplementary material for: Identification of sense and antisense transcripts regulated by drought in sugarcane
Source: Plant Mol Biol. 2012 May 19;79(4):461–77. doi: 10.1007/s11103-012-9922-1 (PMC3369129; doi:10.1007/s11103-012-9922-1)
Supplement: Supplementary file 3 — Electronic Supplementary Table 3 – Genes differentially expressed in sugarcane plants submitted to drought for 24 h, 72 h and 120 h. (PDF 323 kb) [file 11103_2012_9922_MOESM3_ESM.pdf]

IDENTIFICATION OF SENSE AND ANTISENSE TRANSCRIPTS REGULATED BY DROUGHT IN SUGARCANE

Plant Molecular Biology

Carolina Gimiliani Lembke*[1], Milton Yutaka Nishiyama Jr*[1], Paloma Mieko Sato[1], Rodrigo Fandiño de Andrade[1], Glaucia Mendes Souza[1#]

[1]Laboratório de Transdução de Sinal, Departamento de Bioquímica, Instituto de Química, Universidade de São Paulo.

[#]Corresponding author e-mail address: glmsouza@iq.usp.br

transcript_name	probe_name	function_category	structural_category	category	sub_category1	sub_category2	24h drought	72h drought	120h drought	Up freq.	Down freq.	Inside freq.	Total freq.
SCEQLR1093E06.g	GS1_SS_06392_03925	AminoAcid metabolism	aminotransferase	.	Alanine-glyoxylate aminotransferase 2 homolog 3, mitochondrial; Beta-alanine-pyruvate aminotransferase 3;	unknown [Zea mays]			up (4.5300,5.2100)	1	0	0	1
SCRFLR1034F03.g	GS1_SS_08899_20441	AminoAcid metabolism	aminotransferase	Amino acid metabolism	alanine-glyoxylate aminotransferase	hypothetical protein OsJ_018143 [Oryza sativa (japonica cultivar-group)]			up (4.7400,5.7300)	1	0	0	1
SCJLLR1011C10.g	GS1_SS_00118_18846	AminoAcid metabolism	aminotransferase	Carbohydrate metabolism	Carbon fixation	Aspartate transaminase EC 2.6.1.1			up (4.2300,4.3100)	1	0	0	1
SCUTAM2010C11.g	GS1_SS_05426_12819	AminoAcid metabolism	carboxylase	.	Methylcrotonoyl-CoA carboxylase subunit alpha, mitochondrial; 3-methylcrotonyl-CoA carboxylase 1; MCCase subunit alpha; 3-methylcrotonyl-CoA:carbon dioxide ligase subunit alpha;	unknown [Zea mays]			up (2.9900,3.3200)	1	0	0	1
SCJFRZ2007C01.g	GS1_SS_14783_16054	AminoAcid metabolism	Cysteine metabolism	Amino acid metabolism	Cysteine synthase (EC 4.2.99.8)	Os06g0149900 [Oryza sativa (japonica cultivar-group)] putative cysteine synthase [Oryza sativa Japonica Group] Os06g0149900 [Oryza sativa (japonica cultivar-group)]		down (-3.0300,-4.1900)		0	1	0	1
SCEQRT1029F09.g	GS1_SS_11796_19381	AminoAcid metabolism	decarboxylase	Amino acid metabolism	Glutamate decarboxylase (EC 4.1.1.15)	unknown [Zea mays]		up (2.0400,3.1800)		1	0	0	1
SCJLRT1020D01.g	GS1_SS_12558_17983	AminoAcid metabolism	decarboxylase	Amino acid metabolism	serine decarboxylase	retrotransposon protein, putative, unclassified [Oryza sativa (japonica cultivar-group)]			up (4.6500,4.5200)	1	0	0	1
SCVPCL6046C06.g	GS1_SS_05641_02503	AminoAcid metabolism	dehydrogenase	.	Glutamate dehydrogenase; GDH	hypothetical protein LOC100193614 [Zea mays] unknown [Zea mays]			up (4.7600,4.8900)	1	0	0	1
SCVPRT2076C02.g	GS1_SS_11765_09739	AminoAcid metabolism	dehydrogenase	.	Isovaleryl-CoA dehydrogenase, mitochondrial; IVD;	unknown [Zea mays]		up (3.3700,4.1500)	up (5.3000,5.8600)	2	0	0	2
SCQSRT1036B03.g	GS1_SS_12504_13385	AminoAcid metabolism	dehydrogenase	.	Alpha-aminoadipic semialdehyde synthase; cAt-LKR/SDH; LKR/SDH; Includes: Lysine ketoglutarate reductase; LKR; Includes: Saccharopine dehydrogenase; cAt-SDH; SDH	lysine-ketoglutarate reductase/saccharopine dehydr [Zea mays] lysine ketoglutarate reductase/saccharopine dehydrogenase [Zea mays] lysine-ketoglutarate reductase/saccharopine dehydrogenase bifunctional enzyme [Zea mays]		up (5.3700,5.5200)	up (7.0600,6.8600)	2	0	0	2
SCAGLR2011G09.g	GS1_SS_11372_16920	AminoAcid metabolism	hydrolase	Secondary metabolism	putrescine biosynthesis	Agmatinase, mitochondrial precursor (EC 3.5.3.11) (Agmatine ureohydrolase) (AUH)		up (2.2100,2.5200)		1	0	0	1
SCJFLR1073D03.g	GS1_SS_09817_18375	AminoAcid metabolism	isomerase	Secondary metabolism	Glutamate-1-semialdehyde 2,1-aminomutase, chloroplast precursor (GSA)	unknown [Zea mays]		down (-2.8400,-2.8100)		0	1	0	1
SCSGAM1094D07.g	GS1_SS_21852_21710	AminoAcid metabolism	kinase/phosphatase	Amino acid metabolism	aspartate kinase	monofunctional aspartate kinase 1 [Zea mays]		down (-3.1500,-4.7000)	down (-4.7600,-5.3400)	0	2	0	2
SCSGAM1094D07.g	GS1_AS_21852_21709	AminoAcid metabolism	kinase/phosphatase	Amino acid metabolism	aspartate kinase	monofunctional aspartate kinase 1 [Zea mays]			down (-3.6400,-3.6900)	0	1	0	1
SCCCRT1002D04.g	GS1_AS_04538_19991	AminoAcid metabolism	lyase	Amino acid metabolism	hydroxymethylglutaryl-CoA lyase	unknown [Zea mays]		down (-2.6600,-4.1000)		0	1	0	1
SCCCRT1002D04.g	GS1_SS_04538_19992	AminoAcid metabolism	lyase	Amino acid metabolism	hydroxymethylglutaryl-CoA lyase	unknown [Zea mays]			up (4.7500,3.8200)	1	0	0	1
SCRFLB1054G09.g	GS1_SS_09389_18091	AminoAcid metabolism	lyase	Amino acid metabolism	homogentisate 1,2-dioxygenase	hypothetical protein OsI_020541 [Oryza sativa (indica cultivar-group)]			up (4.2700,4.4900)	1	0	0	1
SCEPRT2043A05.g	GS1_SS_04882_15060	AminoAcid metabolism	oxidoreductase	Amino acid metabolism	dihydrodipicolinate reductase	hypothetical protein LOC100194039 [Zea mays] unknown [Zea mays]		down (-2.3500,-3.5400)		0	1	0	1

SCQGLR1041H02.g	GS1_SS_25524_14233	AminoAcid metabolism	oxidoreductase	.	Copper methylamine oxidase; Primary amine oxidase; MAOXII;	hypothetical protein OsJ_014565 [Oryza sativa (japonica cultivar-group)]			up (3.7100,3.9800)	1	0	0	1
SCCCLR2C03D07.g	GS1_SS_11323_12949	AminoAcid metabolism	oxygenase	.	.	unknown [Zea mays]	down (-1.8400,-1.6500)		up (3.1200,4.3500)	1	1	0	2
SCRFRZ3056G09.g	GS1_SS_16192_16179	AminoAcid metabolism	Reductase	Carbohydrate metabolism	nitrate reductase	nitrate reductase [Zea mays]			down (-4.2600,-5.8500)	0	1	0	1
SCEZLB1013C10.g	GS1_SS_08139_00976	AminoAcid metabolism	synthase	.	Non-specific lipid-transfer protein-like protein At5g64080;	unknown [Zea mays]			up (4.1200,6.5400)	1	0	0	1
SCBFRZ2016H04.g	GS1_SS_23025_03030	AminoAcid metabolism	synthase	.	Proline synthetase co-transcribed bacterial homolog protein	unknown [Zea mays]			down (-3.3700,-3.4000)	0	1	0	1
SCAGLR1021D07.g	GS1_SS_21790_21400	AminoAcid metabolism	synthase	Amino acid metabolism	Acetohydroxyacid synthase	Acetolactate synthase 2, chloroplastic; Acetohydroxy-acid synthase 2; acetohydroxyacid synthase [Zea mays]		down (-3.7000,-4.0900)		0	1	0	1
SCAGLR2033D10.g	GS1_SS_17194_20403	AminoAcid metabolism	Transferase family	Stress	Water stress	repressed			down (-3.3600,-3.4400)	0	1	0	1
SCEPRZ1010D11.g	GS1_SS_09749_16325	AminoAcid metabolism	transporter	Transporters	Transmembrane amino acid transporter protein (Aa_trans)	unknown [Zea mays]			down (-3.6400,-4.5000)	0	1	0	1
SCCCFL5058C04.g	GS1_SS_23749_13229	AminoAcid metabolism	Transporters	.	.	hypothetical protein [Vitis vinifera]			down (-3.4800,-3.7800)	0	1	0	1
SCVPST1062B05.g	GS1_SS_00738_19752	AminoAcid metabolism	uncharacterized	Amino acid metabolism	Serpin	Os05g0511800 [Oryza sativa (japonica cultivar-group)] Os05g0511800 [Oryza sativa (japonica cultivar-group)]		down (-2.7700,-2.9800)		0	1	0	1
SCBGST3111B12.g	GS1_SS_18535_09005	AminoAcid metabolism		.	Aspartokinase 1, chloroplastic; Aspartate kinase 1;	monofunctional aspartate kinase 1 [Zea mays]		down (-2.7800,-4.2700)	down (-5.3700,-5.2800)	0	2	0	2
SCAGLR1043B10.g	GS1_SS_18416_17211	Biosynthesis of other Secondary Metabolites	Flavone and flavonol biosynthesis	Cell wall	putative flavonoid 3 hydroxilase (F3H)	CYP75A5 (flavonoid 3-monooxygenase / flavonoid 3-hydroxylase)		down (-2.1700,-5.0700)	down (-3.8900,-5.5500)	0	2	0	2
SCCCCL3003H07.b	GS1_SS_05810_08429	Biosynthesis of other Secondary Metabolites	lyase	.	.	unknown [Zea mays]			up (4.3200,5.0900)	1	0	0	1
SCCCLR1022F07.g	GS1_SS_08126_19540	Biosynthesis of other Secondary Metabolites	oxidoreductase	Secondary metabolism	putative short chain alcohol dehydrogenase	unknown [Zea mays]		down (-2.0400,-3.1700)	down (-4.7500,-4.6100)	0	2	0	2
SCCCRZ1C01E09.g	GS1_SS_10514_16646	Biosynthesis of other Secondary Metabolites	transferase	Secondary metabolism	putative steroid sulfotransferase	unknown [Zea mays]		down (-3.8100,-4.7600)	down (-4.7600,-5.5600)	0	2	0	2
SCSBSB1096D03.g	GS1_SS_19516_18083	Carbohydrate metabolism	aldolase	Bioenergetics	glycolysis	Fructose-bisphosphate aldolase chloroplast precursor (EC 4.1.2.13) (ALDP)		down (-3.4900,-4.7100)	down (-6.3400,-6.3200)	0	2	0	2
SCRULR1020D11.g	GS1_SS_16825_18372	Carbohydrate metabolism	aldolase	Carbohydrate metabolism	glycolysis	Fructose-bisphosphate aldolase			down (-4.8100,-8.7800)	0	1	0	1
SCQSLR1018D02.g	GS1_SS_18505_19852	Carbohydrate metabolism	aldolase	Bioenergetics	glycolysis	Aldolase		down (-3.0900,-3.9800)	down (-6.2400,-6.2400)	0	2	0	2
SCCCLR1001E04.g	GS1_SS_09575_15878	Carbohydrate metabolism	carboxylase	Carbohydrate metabolism	photosynthesis (Calvin cycle)	RUBISCO - small subunit		down (-3.1300,-3.3800)	down (-5.1100,-8.4700)	0	2	0	2
SCCCLR1048B08.g	GS1_SS_10105_14869	Carbohydrate metabolism	carboxylase	Carbohydrate metabolism	Fructose and Mannose Metabolism	Triose-phosphate isomerase			down (-4.0300,-5.5900)	0	1	0	1
SCRFLR1012B07.g	GS1_SS_13556_16301	Carbohydrate metabolism	dehydrogenase	Carbohydrate metabolism	Citrate cycle (TCA cycle)	Succinate dehydrogenase EC 1.3.99.1			up (2.8400,3.6400)	1	0	0	1
SCEPAM2056A01.g	GS1_SS_04079_03112	Carbohydrate metabolism	dehydrogenase	.	.	unknown [Zea mays]		down (-3.7900,-6.0700)		0	1	0	1
SCJFRT1009B10.g	GS1_SS_12181_09892	Carbohydrate metabolism	dehydrogenase	.	Acyl-coenzyme A oxidase 4, peroxisomal; AOX 4; Short-chain acyl-CoA oxidase; SAOX; AtCX4; AtG6; G6p	unknown [Zea mays]			up (3.9100,4.9500)	1	0	0	1
SCSBSB1052D07.g	GS1_SS_21449_18655	Carbohydrate metabolism	dehydrogenase	Carbohydrate metabolism	Glycolysis/Gluconeogenesis	Glyceraldehyde-3-phosphate dehydrogenase EC 1.2.1.12			down (-4.7300,-7.7700)	0	1	0	1
SCVPRZ2037A02.g	GS1_SS_02328_19965	Carbohydrate metabolism	Fructose/Mannose/Galactose	Cell wall	Beta-galactosidase	Os03g0165400 [Oryza sativa (japonica cultivar-group)] Beta-galactosidase 5; Lactase 5; Beta-galactosidase precursor, putative, expressed [Oryza sativa (japonica cultivar-group)] Os03g0165400 [Oryza sativa (japonica cultivar-group)]			down (-3.5600,-4.3800)	0	1	0	1
SCEPAM1022E11.g	GS1_SS_01796_14727	Carbohydrate metabolism	Fructose/Mannose/Galactose	Cell wall	Beta-galactosidase	unknown [Zea mays]			up (3.5600,3.6300)	1	0	0	1
SCJLFL3014G04.g	GS1_SS_25944_16171	Carbohydrate metabolism	Frutose/Manose/Galactose	Cell wall	Beta-galactosidase	hypothetical protein OsI_002513 [Oryza sativa (indica cultivar-group)]		down (-3.1100,-3.8300)	down (-4.7800,-5.1600)	0	2	0	2

SCCCCL4009F05.g	GS1_AS_21374_15488	Carbohydrate metabolism	Glyco-Hydro	Cell wall	Beta-xylosidase/alpha-L-arabinofuranosidase (GH family 3)	Xylan 1,4-beta-xylosidase EC 3.2.1.37			up (4.7400,4.6500)	1	0	0	1
SCCCCL4009F05.g	GS1_SS_21374_15489	Carbohydrate metabolism	Glyco-Hydro	Cell wall	Beta-xylosidase/alpha-L-arabinofuranosidase (GH family 3)	Xylan 1,4-beta-xylosidase EC 3.2.1.37		down (-2.7200,-3.9900)		0	1	0	1
SCCCCL7C02F04.g	GS1_AS_21108_19494	Carbohydrate metabolism	glycosyltransferases	Carbohydrate metabolism	Putative 4-alpha-glucanotransferase	disproportionating enzyme [Aegilops tauschii]			up (3.7900,3.1300)	1	0	0	1
SCACAD1037B06.g	GS1_SS_00142_20039	Carbohydrate metabolism	Hydratase	Bioenergetics	glyoxylate cycle	Aconitate hydratase cytoplasmic (EC 4.2.1.3)(Citrate hydro-lyase)(Aconitase)(Aconitase)		up (2.3400,2.5900)		1	0	0	1
SCJLLR1107D06.g	GS1_SS_11621_09365	Carbohydrate metabolism	hydrolase	.	.	unknown [Zea mays]		up (7.1400,3.6800)	up (3.7600,7.5500)	2	0	0	2
SCEPAM2053H08.g	GS1_SS_04043_15271	Carbohydrate metabolism	hydrolase	Carbohydrate metabolism	Starch and sucrose metabolism	1,4-alpha-glucan branching enzyme EC 2.4.1.18		down (-2.8600,-3.6000)		0	1	0	1
SCCCST1C02D05.g	GS1_SS_17710_19657	Carbohydrate metabolism	hydrolase	Carbohydrate metabolism	Aminosugars metabolism	beta-N-Acetylhexosaminidase EC 3.2.1.52		down (-6.0000,-5.2600)	down (-3.4800,-4.1900)	0	2	0	2
SCCCCL3002B11.b	GS1_SS_05765_17065	Carbohydrate metabolism	hydrolase	Carbohydrate metabolism	Starch and sucrose metabolism	Alpha amylase EC 3.2.1.1			up (3.4800,4.4500)	1	0	0	1
SCMCRT2086D03.g	GS1_SS_11933_07426	Carbohydrate metabolism	kinase/phosphatase	.	Hexokinase-7; Hexokinase-6	unknown [Zea mays]			up (4.7100,2.9100)	1	0	0	1
SCJFRZ2007A03.g	GS1_SS_11611_11276	Carbohydrate metabolism	kinase/phosphatase	Protein kinase	other	canePEPC kinase-1 (Phosphoenolpyruvate carboxylase kinase)	down (-1.4900,-2.0700)	up (3.9500,2.8300)		1	1	0	2
SCJFRT1060F11.g	GS1_SS_12311_21183	Carbohydrate metabolism	kinase/phosphatase	Carbohydrate metabolism	Photosynthesis	Pyruvate,orthophosphate dikinase			down (-3.3500,-4.2200)	0	1	0	1
SCBGST3105A06.g	GS1_AS_18508_15469	Carbohydrate metabolism	kinase/phosphatase	Carbohydrate metabolism	Pentose phosphate pathway	Fructose-bisphosphatase Hexosediphosphatase EC 3.1.3.11			down (-2.7300,-3.1200)	0	1	0	1
SCBGLR1115H04.g	GS1_SS_17458_16314	Carbohydrate metabolism	kinase/phosphatase	Carbohydrate metabolism	Carbon fixation	Sedoheptulose bisphosphatase EC 3.1.3.37			down (-2.9700,-4.2500)	0	1	0	1
SCCCLR1048E03.g	GS1_SS_17069_03411	Carbohydrate metabolism	kinase/phosphatase	.	Alpha-glucan water dikinase, chloroplastic; Starch-related R1 protein;	Os06g0498400 [Oryza sativa (japonica cultivar-group)] Os06g0498400 [Oryza sativa (japonica cultivar-group)]		down (-3.2100,-3.1500)		0	1	0	1
SCEZLB1006F11.g	GS1_SS_17085_18093	Carbohydrate metabolism	kinase/phosphatase	Carbohydrate metabolism	Glycolysis/Gluconeogenesis	Phosphoglycerate kinase EC 2.7.2.3			down (-4.5300,-6.1600)	0	1	0	1
SCBGST3105A06.g	GS1_SS_18508_15468	Carbohydrate metabolism	kinase/phosphatase	Carbohydrate metabolism	Pentose phosphate pathway	Fructose-bisphosphatase Hexosediphosphatase EC 3.1.3.11			down (-3.2400,-5.1400)	0	1	0	1
SCEPRT2048D06.g	GS1_SS_06461_16032	Carbohydrate metabolism	kinase/phosphatase	Carbohydrate metabolism	Fructose and Mannose Metabolism	6-Phosphofructokinase EC 2.7.1.11			down (-5.0900,-4.5200)	0	1	0	1
SCEPAM1015F07.g	GS1_SS_04314_15179	Carbohydrate metabolism	kinase/phosphatase	Carbohydrate metabolism	Alpha-glucan phosphorylase (starch phosphorylase)	starch phosphorylase 1 precursor [Zea mays]		down (-2.7200,-4.7600)		0	1	0	1
SCUTAM2089E05.g	GS1_SS_05498_17977	Carbohydrate metabolism	lyase	Carbohydrate metabolism	Starch and sucrose metabolism	Beta-Amylase EC 3.2.1.2		down (-4.4800,-7.4300)	down (-5.5500,-5.8500)	0	2	0	2
SCUTLR2030E06.g	GS1_SS_11655_20223	Carbohydrate metabolism	lyase	Carbohydrate metabolism	Starch and sucrose metabolism	Amylophosphorylase, alpha-glucan phosphorylase EC 2.4.1.1		down (-3.8200,-4.5000)		0	1	0	1
SCCCST2002A09.g	GS1_SS_07635_18343	Carbohydrate metabolism	mutase	Bioenergetics	glycolysis	23-bisphosphoglycerate-independent phosphoglycerate mutase (EC 5.4.2.1)(Phosphoglyceromut		up (4.5300,4.3300)	up (3.4400,4.4800)	2	0	0	2
SCCCLR1C02C02.g	GS1_SS_05575_08561	Carbohydrate metabolism	Nucleotide sugar synthesis, conversion and transport	Cell wall	UDP-glucuronate 4-epimerase	unknown [Zea mays]		down (-4.2800,-3.2200)		0	1	0	1
SCCCRT1C05A07.g	GS1_SS_24436_08415	Carbohydrate metabolism	others	.	Beta-hexosaminidase subunit B2; N-acetyl-beta-glucosaminidase subunit B2; Beta-N-acetylhexosaminidase subunit B2;	Os01g0891000 [Oryza sativa (japonica cultivar-group)] putative beta-N-acetylhexosaminidase [Oryza sativa Japonica Group] Os01g0891000 [Oryza sativa (japonica cultivar-group)]		down (-5.8500,-6.3900)	down (-4.2500,-4.7100)	0	2	0	2
SCCCLR1048E04.g	GS1_SS_07122_06922	Carbohydrate metabolism	others	.	Uncharacterized protein YKL128C	OSIGBa0123D13.3 [Oryza sativa (indica cultivar-group)]		up (1.9600,4.3000)	up (4.8000,4.1600)	2	0	0	2
SCACSB1117A12.g	GS1_SS_20946_16760	Carbohydrate metabolism	others	Carbohydrate metabolism	Alpha-glucan phosphorylase (starch phosphorylase)	starch phosphorylase 1 precursor [Zea mays]		down (-3.2500,-5.5500)		0	1	0	1
SCCCCL6C03G01.g	GS1_SS_06606_16330	Carbohydrate metabolism	others	Pathogenicity	Protease inhibitors	Kunitz			up (4.2000,4.5400)	1	0	0	1
SCEPRT2048D07.g	GS1_SS_12960_09973	Carbohydrate metabolism	oxidoreductase	.	Probable NAD(P)H-dependent oxidoreductase 1	unknown [Zea mays]		up (3.0400,3.0200)		1	0	0	1
SCJLLR1103A10.g	GS1_SS_20438_20605	Carbohydrate metabolism	protein-protein interaction	Carbohydrate metabolism	Putative 32.7 kDa jasmonate-induced protein	hypothetical protein OsI_036610 [Oryza sativa (indica cultivar-group)]		down (-3.6600,-7.6000)		0	1	0	1
SCEQLR1094C07.g	GS1_SS_10405_10135	Carbohydrate metabolism	Reductase	.	Uncharacterized oxidoreductase At1g06690, chloroplastic;	unknown [Zea mays]			down (-3.3300,-3.2700)	0	1	0	1

SCEQAM1036A06.g	GS1_SS_02122_15843	Carbohydrate metabolism	Sacarose/trealose/sorbitol e outros mono-oligosacarideos	Cell wall	sucrose-phosphate synthase	Sucrose phosphate synthase EC 2.4.1.14			down (-2.8500,-4.8600)	0	1	0	1
SCJFRZ2029F03.g	GS1_SS_11586_15029	Carbohydrate metabolism	several pathways (several putative substrates)	Cell wall	Glycoside hydrolase family 31 (alpha-glucosidase or alpha-xylosidase)	Alpha-Glucosidase EC 3.2.1.20		down (-3.2900,-4.0200)		0	1	0	1
SCPIRT3021H05.g	GS1_SS_24492_21541	Carbohydrate metabolism	several pathways (several putative substrates)	Cell wall	glucosyltransferase	ref|NP_910035.1| putative UDP-glucosyltransferase [Oryza sativa (japonica cultivar-group)] gb|AAO18436.1| putative UDP-glucosyltransferase [Oryza sativa (japonica cultivar-group)]		down (-4.7200,-5.9200)	down (-6.1400,-7.0600)	0	2	0	2
SCMCRT2103F09.g	GS1_SS_13619_18216	Carbohydrate metabolism	several pathways (several putative substrates)	Cell wall	UDP- glucosyltransferase	gb|AAF17077.1| UDP-glucose glucosyltransferase [Sorghum bicolor]		down (-2.4300,-4.3000)	down (-4.6000,-5.4100)	0	2	0	2
SCBGSB1026A09.g	GS1_SS_33513_19414	Carbohydrate metabolism	several pathways (several putative substrates)	Cell wall	Glycosyl hydrolase family 1	Os01g0508000 [Oryza sativa (japonica cultivar-group)] putative beta-glucosidase [Oryza sativa Japonica Group] Os01g0508000 [Oryza sativa (japonica cultivar-group)]			down (-3.4200,-3.9000)	0	1	0	1
SCEQHR1082B01.g	GS1_SS_14031_15728	Carbohydrate metabolism	several pathways (several putative substrates)	Cell wall	Glycosyl hydrolase family 1	hypothetical protein OsI_030804 [Oryza sativa (indica cultivar-group)]		down (-4.4400,-7.3600)	down (-6.0200,-7.2500)	0	2	0	2
SCSBSB1057E03.g	GS1_SS_17065_21024	Carbohydrate metabolism	several pathways (several putative substrates)	Cell wall	Glycosyl hydrolase family 1	hypothetical protein OsI_002091 [Oryza sativa (indica cultivar-group)]			down (-4.5700,-4.5300)	0	1	0	1
SCSGAM2105D04.g	GS1_SS_23663_18537	Carbohydrate metabolism	several pathways (several putative substrates)	Cell wall	UDP- glucosyltransferase	induced			down (-4.9100,-6.0400)	0	1	0	1
SCMCRZ3068G10.g	GS1_SS_16085_12578	Carbohydrate metabolism	several pathways (several putative substrates)	Cell wall	Galactosyltransferase	unknown [Zea mays]			up (3.9400,5.4800)	1	0	0	1
SCAGST3140F06.g	GS1_SS_12529_20983	Carbohydrate metabolism	several pathways (several putative substrates)	Cell wall	UDP- glucosyltransferase	hypothetical protein OsJ_021002 [Oryza sativa (japonica cultivar-group)]		up (2.2400,2.4900)		1	0	0	1
SCJFHR1C03E12.b	GS1_SS_07966_02619	Carbohydrate metabolism	several pathways (several putative substrates)	Cell wall	Glycosyl hydrolase family 1	unknown [Zea mays]		up (2.8200,3.1500)		1	0	0	1
SCBFAM2022B05.g	GS1_SS_20357_15309	Carbohydrate metabolism	several pathways (several putative substrates)	Cell wall	Galactosyltransferase	unknown [Zea mays] unknown [Zea mays]			down (-3.3800,-4.1100)	0	1	0	1
SCCCLB1021E06.g	GS1_SS_18463_21827	Carbohydrate metabolism	Starch synthase EC 2.4.1.21	Carbohydrate metabolism	Starch and sucrose metabolism	Starch synthase EC 2.4.1.21			down (-6.4200,-5.9800)	0	1	0	1
SCEPCL6023F02.g	GS1_SS_00431_19531	Carbohydrate metabolism	sucrose methabolism	Cell wall	sucrose synthase	Sucrose synthase EC 2.4.1.13		up (4.3300,3.2800)		1	0	0	1
SCJFLR1017E09.g	GS1_AS_08724_06084	Carbohydrate metabolism	synthase	.	Uncharacterized glycosyltransferase At1g55740	unknown [Zea mays]			up (3.9600,4.4900)	1	0	0	1
SCJLRT2052C09.g	GS1_SS_13522_14567	Carbohydrate metabolism	synthase	Carbohydrate metabolism	chalcone synthase	unknown [Zea mays]			up (5.8600,4.8800)	1	0	0	1
SCJFLR1017E09.g	GS1_SS_08724_06083	Carbohydrate metabolism	synthase	.	Uncharacterized glycosyltransferase At1g55740	unknown [Zea mays]		up (4.6700,2.3600)		1	0	0	1
SCCCAD1004H06.g	GS1_SS_00075_12059	Carbohydrate metabolism	synthase	.	Inositol-3-phosphate synthase; Myo-inositol-1-phosphate synthase; MI-1-P synthase; IPS	unknown [Zea mays]		up (6.4900,4.1600)		1	0	0	1
SCCCCL4001F08.g	GS1_SS_05956_20395	Carbohydrate metabolism	synthase	Carbohydrate metabolism	Glyoxylate cycle	malate synthase		up (6.8900,9.1600)	up (8.3500,8.3000)	2	0	0	2
SCAGCL6016B11.g	GS1_SS_06470_14954	Carbohydrate metabolism	synthase	Carbohydrate metabolism	Trehalose 6-phosphate synthase	unknown [Zea mays]		up (2.0800,5.3500)	up (5.0500,4.8700)	2	0	0	2
SCBFLR1039F10.g	GS1_SS_19267_20380	Carbohydrate metabolism	transferase	Carbohydrate metabolism	Starch and sucrose metabolism	Glucose 1-phosphate adenylyltransferase EC 2.7.7.27			down (-3.1300,-2.4800)	0	1	0	1
SCCCFL4002D04.g	GS1_SS_19851_18640	Carbohydrate metabolism	transferase	Carbohydrate metabolism	Starch and sucrose metabolism	Glucose 1-phosphate adenylyltransferase EC 2.7.7.27			down (-3.3100,-3.7000)	0	1	0	1
SCSBSB1057D05.g	GS1_SS_16966_14693	Carbohydrate metabolism	transferase	Carbohydrate metabolism	Alpha-glucan phosphorylase (starch phosphorylase)	starch phosphorylase 1 precursor [Zea mays]		down (-2.6900,-5.2400)		0	1	0	1
SCCCLR1C01F04.g	GS1_SS_12391_12149	Carbohydrate metabolism	Transferase family	.	Probable UDP-glucosyl transferase At1g05670	hypothetical protein OsJ_028867 [Oryza sativa (japonica cultivar-group)]			up (3.2500,3.7100)	1	0	0	1
SCCCLR1048C12.g	GS1_SS_10109_00767	Carbohydrate metabolism	Transferase family	.	Uncharacterized glycosyltransferase At1g55740	unknown [Zea mays]			up (4.5400,4.1000)	1	0	0	1

SCVPCL6061A06.g	GS1_SS_23499_16674	Carbohydrate metabolism	Transferase family	Carbohydrate metabolism	Glucose 1-phosphate adenylyltransferase (large subunit)	putative glucose-1-phosphate adenylyltransferase large subunit 4 precursor [Zea mays]			down (-2.8800,-3.9900)	0	1	0	1
SCUTST3085B04.g	GS1_SS_34834_14615	Carbohydrate metabolism	Transferase family	Carbohydrate metabolism	Phosphoglucomutase	unknown [Zea mays]			down (-3.7900,-6.1200)	0	1	0	1
SCCCLR1072D05.g	GS1_SS_10125_06806	Carbohydrate metabolism	translocator	.	Protein RUPTURED POLLEN GRAIN 1	unknown [Zea mays]		up (3.5100,3.0500)	up (4.4600,4.2600)	2	0	0	2
SCSFRT2067F07.g	GS1_SS_16058_18459	Carbohydrate metabolism	transporter	Transporters	Sugar transporters	putative sugar transporter type 2a [Saccharum hybrid cultivar]			down (-5.0000,-3.3000)	0	1	0	1
SCRLRZ3043D02.g	GS1_SS_23626_18022	Carbohydrate metabolism	Transporters	Transporters	Sugar transporters	putative monosaccharide transporter [Oryza sativa (japonica			down (-4.8200,-5.2900)	0	1	0	1
SCBFRT1064F09.g	GS1_SS_11766_12374	Carbohydrate metabolism	Trehalose metabolism	.	Probable alpha,alpha-trehalose-phosphate synthase [UDP-forming] 9; Trehalose-6-phosphate synthase 9; AtTPS9	hypothetical protein LOC100191265 [Zea mays] unknown [Zea mays]			up (4.2300,5.2800)	1	0	0	1
SCSGSB1009B08.b	GS1_SS_17356_10984	Carbohydrate metabolism		.	Soluble starch synthase 2-2, chloroplastic/amyloplastic; Soluble starch synthase II-2;	starch synthase IIb-2 precursor [Zea mays] starch synthase IIb-2 precursor [Zea mays]			down (-4.4000,-7.0500)	0	1	0	1
SCEQRT2092D05.g	GS1_SS_06885_18778	Carbohydrate metabolism		Carbohydrate metabolism	Glycolysis/Gluconeogenesis	Pyruvate decarboxylase EC 4.1.1.1			up (2.9200,3.9500)	1	0	0	1
SCSGFL4196D07.b	GS1_SS_24381_01934	Carotenoid Metabolism	synthase	.	Phytoene synthase, chloroplastic;	chloroplast phytoene synthase 1 [Sorghum bicolor]			down (-3.0400,-5.1200)	0	1	0	1
SCACAM2044B11.g	GS1_SS_22983_21250	Carotenoid Metabolism	synthase	Lipid, fatty-acid and isoprenoid metabolism	Phytoene synthase, chloroplast precursor (EC 2.5.1.-)	chloroplast phytoene synthase 1 [Sorghum bicolor]			down (-3.6800,-5.2200)	0	1	0	1
SCEPCL6029D04.g	GS1_SS_06635_14709	Cell cycle	cyclin	Cell cycle	cyclin	Cyclin type B-like		up (2.7300,3.5500)	up (3.5500,4.3500)	2	0	0	2
SCCCRZ1C01E03.g	GS1_SS_00247_17909	Cell cycle	DNA replication factor	Cell cycle	CDC	replication licensing factor MCM4			down (-3.0700,-5.7000)	0	1	0	1
SCCCLR1C05C04.g	GS1_SS_14930_16317	Cell cycle	DNA replication factor	Cell cycle	PROLIFERA protein	replication licensing factor MCM7 homologue [Zea mays] replication licensing factor MCM7 homologue [Zea mays]			down (-3.0800,-5.6900)	0	1	0	1
SCEPCL6029A03.g	GS1_SS_00447_18841	Cell cycle	DNA replication factor	Cell cycle	DNA replication licensing factor mcm6 (Minichromosome maintenance protein 6)	minichromosome maintenance protein [Zea mays] minichromosome maintenance protein [Zea mays]			down (-2.4800,-4.5100)	0	1	0	1
SCCCLR1072B11.g	GS1_SS_02556_20202	Cell cycle	DNA replication factor	Cell cycle	DNA replication licensing factor MCM3 homolog (Replication origin activator) (ROA protein)	replication origin activator 2 [Zea mays] DNA replication licensing factor MCM3 homolog 2; Replication origin activator 2; ROA protein 2 replication origin activator 2 [Zea mays]			down (-3.9700,-4.8000)	0	1	0	1
SCBFSB1047G04.g	GS1_SS_16748_10326	Cell cycle	others	.	Cyclin-P4-1; CycP4;1	unknown [Zea mays]		down (-2.8800,-4.8700)	down (-6.5000,-6.9300)	0	2	0	2
SCJFRZ2011A04.g	GS1_AS_14041_19022	Cell cycle	quinase	Protein kinase	cell cycle-related	caneCDC2-5		up (2.5500,3.2700)		1	0	0	1
SCVPLR1049E05.g	GS1_SS_21231_17810	Cell cycle		Cell cycle	cyclin	Cyclin Ia			down (-4.8100,-3.7600)	0	1	0	1
SCCCRT1002G03.g	GS1_SS_26188_15792	Cell wall metabolism	cell wall differentiation	Cell wall	Glycosyl hydrolase family 3 protein (Xylosidase)	Xylan 1,4-beta-xylosidase EC 3.2.1.37		up (3.5200,3.5300)		1	0	0	1
SCCCLR1C06D03.g	GS1_SS_12746_10371	Cell wall metabolism	cell wall differentiation	Cell wall	alpha-l-fucosidase	unknown [Zea mays]			down (-4.2100,-3.4900)	0	1	0	1
SCCCRT1002F03.g	GS1_SS_11715_16904	Cell wall metabolism	cell wall differentiation	Cell wall	Xyloglucan endotransglycosylase/hydrolase (XTH)	unknown [Zea mays]		down (-4.1600,-3.9500)	down (-3.6400,-6.1000)	0	2	0	2
SCCCLB1024D03.g	GS1_SS_08994_10758	Cell wall metabolism	cell wall differentiation	Cell wall	Pectinacetylesterase family protein	unknown [Zea mays]		down (-4.2900,-4.1500)	down (-4.2800,-3.8400)	0	2	0	2
SCCCCL7038A01.g	GS1_SS_07217_18359	Cell wall metabolism	cell wall differentiation	Cell wall	Mannan endo-1,4-beta-mannosidase	hypothetical protein LOC100191416 [Zea mays] unknown [Zea mays]		down (-2.8400,-7.5700)	down (-7.9700,-6.9600)	0	2	0	2
SCCCLB1023H08.g	GS1_SS_21848_03892	Cell wall metabolism	Cell wall growth/extension	Cell wall	Expansin	unknown [Zea mays]		down (-4.6900,-5.2800)	down (-5.6200,-5.7600)	0	2	0	2
SCCCLR1065A10.g	GS1_SS_15508_08491	Cell wall metabolism	Cell wall growth/extension	Cell wall	Expansin	unknown [Zea mays]		down (-3.2200,-4.5800)	down (-4.8100,-4.6100)	0	2	0	2
SCCCST1004C07.g	GS1_SS_17511_10748	Cell wall metabolism	cell wall growth/extension	Cell wall	Expansin	unknown [Zea mays]		up (5.9500,3.8600)	up (4.8600,7.0500)	2	0	0	2
SCEQRT2026A06.g	GS1_SS_12709_16322	Cell wall metabolism	Cell wall growth/extension	Cell wall	Expansin	Putative selT-like protein F28H7.4 precursor			up (8.8100,5.3500)	1	0	0	1
SCCCCL4006H09.g	GS1_SS_07367_19801	Cell wall metabolism	Cell wall growth/extension	Cell wall	Expansin	unknown [Zea mays]		down (-3.8900,-6.5400)	down (-4.8300,-5.6100)	0	2	0	2
SCEQRT2027A10.g	GS1_SS_09271_13480	Cell wall metabolism	Cell wall growth/extension	Cell wall	Expansin	alpha expansin1 [Zea mays] alpha-expansin 1 [Zea mays] unknown [Zea mays]		down (-5.9700,-7.1600)	down (-6.0100,-8.0600)	0	2	0	2
SCEPRT2043B01.g	GS1_SS_12904_00991	Cell wall metabolism	Cell wall growth/extension	Cell wall	Expansin	unknown [Zea mays]			down (-3.2300,-6.2500)	0	1	0	1

SCCCLR1C06D07.g	GS1_SS_02595_16779	Cell wall metabolism	dehydrogenase	Amino acid metabolism	betaine-aldehyde dehydrogenase	putative cytosolic aldehyde dehydrogenase [Oryza sativa Japonica Group] hypothetical protein OsJ_020998 [Oryza sativa (japonica cultivar-group)]			down (-3.7000,-5.6100)	0	1	0	1
SCMCSD1062A02.g	GS1_SS_25899_16947	Cell wall metabolism	Lignin metabolism	Cell wall	dirigent-like protein	Disease resistance response protein-related			up (4.5100,3.9400)	1	0	0	1
SCCCLR1066B10.g	GS1_SS_13278_17807	Cell wall metabolism	N-Glycan biosynthesis	Cell wall	Cellulose synthase (CesA)	hypothetical protein OsI_024417 [Oryza sativa (indica cultivar-group)]		down (-2.4100,-4.5200)		0	1	0	1
SCCCRZ2002G07.g	GS1_SS_12360_09060	Cell wall metabolism	N-Glycan biosynthesis	Cell wall	Cellulose synthase (CesA)	cellulose synthase7 [Zea mays] cellulose synthase-7 [Zea mays]			down (-3.4000,-5.6600)	0	1	0	1
SCEQLB1066G04.g	GS1_SS_00806_21326	Cell wall metabolism	N-Glycan biosynthesis	Cell wall	Cellulose synthase like (CSL)	Cellulose synthase (UDP-forming) EC 2.4.1.12			down (-3.2300,-5.0000)	0	1	0	1
SCCCLR2C02H01.g	GS1_SS_01270_18061	Cell wall metabolism	N-Glycan biosynthesis	Cell wall	Cellulose synthase (CesA)	Cellulose synthase (UDP-forming) EC 2.4.1.12		down (-4.1300,-9.0700)	down (-4.0900,-9.1200)	0	2	0	2
SCQGRT1044A07.g	GS1_SS_12699_09207	Cell wall metabolism	N-Glycan biosynthesis	Cell wall	Cellulose synthase	hypothetical protein LOC100193757 [Zea mays] unknown [Zea mays]		down (-4.7600,-5.5500)	down (-4.7200,-6.6900)	0	2	0	2
SCJLLR1106H06.g	GS1_SS_16877_07928	Cell wall metabolism	N-Glycan biosynthesis	Cell wall	Cellulose synthase (CesA)	cellulose synthase10 [Zea mays] cellulose synthase catalytic subunit 10 [Zea mays]		down (-3.7200,-8.3200)	down (-3.8900,-8.6900)	0	2	0	2
SCJLFL3019G10.g	GS1_SS_22523_09248	Cell wall metabolism	N-Glycan biosynthesis	Cell wall	cellulose synthase like (CSL)	unknown [Zea mays]		down (-2.6900,-4.3500)	down (-7.2200,-7.4400)	0	2	0	2
SCJFRT1008G05.g	GS1_SS_13327_19746	Cell wall metabolism	Other glycan degradation	Cell wall	beta-1,3-glucanase Glycosyl hydrolases family 17	unknown [Zea mays]		up (2.6400,4.3600)		1	0	0	1
SCUTLR2030G02.g	GS1_SS_00752_15697	Cell wall metabolism	Other glycan degradation	Cell wall	endo-1,4-beta-glucanase Glycosyl hydrolase family 9	Cellulase, Endo-1,4-beta-glucanase EC 3.2.1.4			down (-3.5900,-5.1600)	0	1	0	1
SCEQRT2028G11.g	GS1_SS_07850_21784	Cell wall metabolism	Other glycan degradation	Cell wall	beta-1,3-glucanase Glycosyl hydrolases family 17	unknown [Zea mays]		up (3.3300,4.2900)	up (3.9700,3.9500)	2	0	0	2
SCCCLB2007D02.g	GS1_SS_40273_17948	Cell wall metabolism	Other glycan degradation	Cell wall	pectinesterase family protein	hypothetical protein OsJ_024553 [Oryza sativa (japonica cultivar-group)]			down (-4.0600,-5.3200)	0	1	0	1
SCJFAM1069C01.g	GS1_SS_02484_19499	Cell wall metabolism	Other glycan degradation	Cell wall	Glycoside hydrolase family 28 protein (polygalacturonase)	hypothetical protein OsI_006479 [Oryza sativa (indica cultivar-group)]			down (-3.2400,-3.9500)	0	1	0	1
SCJLAM2093B10.g	GS1_SS_22322_21316	Cell wall metabolism	Other glycan degradation	Cell wall	beta-1,3-glucanase Glycosyl hydrolases family 17	induced		down (-3.4000,-5.1300)	down (-5.7900,-5.9900)	0	2	0	2
SCJLRT1023E06.g	GS1_SS_12580_08945	Cell wall metabolism	Other glycan degradation	Cell wall	beta-1,3-glucanase Glycosyl hydrolases family 17	pathogenesis-related protein 6 [Zea mays subsp. parviglumis]			up (8.4100,6.2400)	1	0	0	1
SCEQRT1026E05.g	GS1_SS_00904_10764	Cell wall metabolism	Other glycan degradation	Cell wall	beta-1,3-glucanase Glycosyl hydrolases family 17	hypothetical protein OsI_005053 [Oryza sativa (indica cultivar-group)]			up (5.7500,3.2900)	1	0	0	1
SCEPRZ1009D10.g	GS1_SS_23462_16559	Cell wall metabolism	Other glycan degradation	Cell wall	pectinesterase family protein	Os06g0193200 [Oryza sativa (japonica cultivar-group)] putative pectin methylesterase [Oryza sativa Japonica Group] Os06g0193200 [Oryza sativa (japonica cultivar-group)]		down (-3.8400,-3.9800)		0	1	0	1
SCBGLR1113B02.g	GS1_SS_09971_00713	Cell wall metabolism	others	.	COBRA-like protein 2; Protein BRITTLE CULM1-like 2;	hypothetical protein OsJ_010812 [Oryza sativa (japonica cultivar-group)]		up (4.1600,4.2200)	up (3.3100,4.8300)	2	0	0	2
SCCCLR1078D02.g	GS1_SS_16898_19287	Cell wall metabolism	others	Carbohydrate metabolism	1,4-alpha-glucan branching enzyme (starch branching enzyme)	starch branching enzyme IIa [Zea mays]		down (-3.3600,-3.5400)		0	1	0	1
SCCCCL4015F12.g	GS1_SS_24513_00878	Cell wall metabolism	peptidase	.	Basic 7S globulin; SBg7S; Bg; Basic 7S globulin high kDa subunit; Basic 7S globulin low kDa subunit;	unknown [Zea mays]			up (3.8700,3.1600)	1	0	0	1
SCEQLR1029E05.g	GS1_SS_15544_17574	Cell wall metabolism	Phenylpropanoid biosynthesis	Cell wall	Cinnamyl alcohol dehydrogenase (CAD)	Mannitol dehydrogenase EC 1.1.2.2		down (-2.7700,-4.3300)	down (-3.6900,-3.1100)	0	2	0	2
SCCCCL6024F07.g	GS1_AS_25287_16689	Cell wall metabolism	Phenylpropanoid biosynthesis	Cell wall	Cinnamoyl-coA reductase (CCR)	Os02g0808800 [Oryza sativa (japonica cultivar-group)] cinnamoyl CoA reductase [Oryza sativa Japonica Group] cinnamoyl CoA reductase [Oryza sativa Japonica Group] Os02g0808800 [Oryza sativa (japonica cultivar-group)] hypothetical protein OsJ_008505 [O		up (3.8100,4.0300)		1	0	0	1

SCCCCL6024F07.g	GS1_SS_25287_16690	Cell wall metabolism	Phenylpropanoid biosynthesis	Cell wall	Cinnamoyl-coA reductase (CCR)	Os02g0808800 [Oryza sativa (japonica cultivar-group)] cinnamoyl CoA reductase [Oryza sativa Japonica Group] cinnamoyl CoA reductase [Oryza sativa Japonica Group] Os02g0808800 [Oryza sativa (japonica cultivar-group)] hypothetical protein OsJ_008505 [O		up (7.2400,5.5100)	up (7.8300,7.3000)	2	0	0	2
SCEQSB1C06D05.g	GS1_SS_36256_18097	Cell wall metabolism	Phenylpropanoid biosynthesis	Cell wall	Cinnamoyl-coA reductase (CCR)	cinnamoyl-CoA reductase [Saccharum officinarum]		down (-2.4900,-6.8500)	down (-3.2800,-7.4200)	0	2	0	2
SCSGAM1094D05.g	GS1_SS_16141_17170	Cell wall metabolism	Phenylpropanoid biosynthesis	Cell wall	phenylalanine ammonia-lyaseÃ,Â (PAL)	Phenylalanine ammonia-lyase		down (-2.0100,-2.8300)		0	1	0	1
SCRUAD1064C10.g	GS1_SS_20738_18502	Cell wall metabolism	Phenylpropanoid biosynthesis	Cell wall	putative ferulate 5 hydroxilase (F5H); flavonoid 3 hydroxilase (F3H)	CYP75A6 (flavonoid 3l 5l-hydroxylase)		down (-2.6400,-4.2400)		0	1	0	1
SCACHR1038E08.g	GS1_SS_24520_21449	Cell wall metabolism	Phenylpropanoid biosynthesis	Cell wall	Cinnamyl alcohol dehydrogenase (CAD)	Mannitol dehydrogenase (cytochrome) EC 1.1.2.2			down (-4.7000,-4.5900)	0	1	0	1
SCRFLR1034D04.g	GS1_SS_15437_21186	Cell wall metabolism	Structural proteins of the cell wall	Cell wall	Fasciclin-like arabinogalactan protein	hypothetical protein LOC100192072 [Zea mays] unknown [Zea mays]		down (-2.0500,-5.4300)	down (-3.0000,-4.3100)	0	2	0	2
SCJLRZ3077F12.g	GS1_SS_16018_00210	Cell wall metabolism	synthase	.	Dehydrodolichyl diphosphate synthase 2 (Dedol-PP synthase 2)	unknown [Zea mays]		up (6.0000,4.9000)	up (5.5200,4.8100)	2	0	0	2
SCCCLR1048E10.g	GS1_SS_10115_00001	Circadian Clock	nucleic acid binding	Transcription Factor	MYB	LHY/CCA1		up (4.5300,3.1300)		1	0	0	1
SCCCLR1066D01.g	GS1_SS_20048_09290	Cytoskeleton and vesicle transport	others	.	.	hypothetical protein OsJ_027041 [Oryza sativa (japonica cultivar-group)]			down (-4.1800,-5.1800)	0	1	0	1
SCACLR2014H03.g	GS1_SS_18482_16104	Cytoskeleton and vesicle transport	SNARE	Stress	.	ST3>ST1		down (-3.3000,-5.5900)		0	1	0	1
SCACSD2018E08.g	GS1_SS_25481_14374	Development	others	.	Stem 28 kDa glycoprotein precursor (Vegetative storage protein A)	unknown [Zea mays]		down (-3.1500,-5.9000)	down (-5.9000,-5.7400)	0	2	0	2
SCBFSD2035E11.g	GS1_SS_25518_18146	Development	others	Protein kinase	undefined	ZCN14 protein [Zea mays] ZCN14 [Zea mays] ZCN14 [Zea mays]		up (3.1500,4.3300)		1	0	0	1
SCCCLR1080F11.g	GS1_SS_10285_01760	Development	synthase	Secondary metabolism	Acyl-CoA synthetases (AMP-forming)/AMP-acid ligases II	unknown [Zea mays]		up (5.1700,5.1000)	up (3.6200,5.7100)	2	0	0	2
SCRUAD1132D09.g	GS1_SS_21620_12252	Development	Uncharacterized	Stress	cytochrome P450	CYP75A5 (flavonoid 3-monooxygenase / flavonoid 3-hydroxylase)			up (3.5900,3.1800)	1	0	0	1
SCUTRZ3071H01.g	GS1_SS_16511_04168	DNA metabolism	helicase	.	DEAD-box ATP-dependent RNA helicase 3	unknown [Zea mays]			down (-4.3500,-3.5800)	0	1	0	1
SCCCRZ2C03C04.g	GS1_SS_09916_17814	DNA metabolism	histone	Amino acid metabolism	Glutamine synthetase (Glutamate--ammonia ligase)	ST3>ST1		down (-2.8000,-3.6800)	down (-3.9500,-4.1900)	0	2	0	2
SCRURT2005G01.g	GS1_SS_03591_13833	DNA metabolism	kinase/phosphatase	.	Deoxycytidine kinase; dCK	unknown [Zea mays]	down (-1.4600,-2.1200)	down (-2.3400,-7.6000)		0	2	0	2
SCQGFL3050B07.g	GS1_SS_22415_09054	DNA metabolism	methyltransferase	.	N(6)-adenine-specific DNA methyltransferase 2	unknown [Zea mays]		down (-2.9800,-3.4000)		0	1	0	1
SCJFRZ2014D06.g	GS1_SS_12278_15377	DNA metabolism	transcription factor	Stress	Water stress	induced		up (4.2300,3.9100)	up (5.1200,3.6400)	2	0	0	2
SCJFRT1058F06.g	GS1_AS_12277_00181	DNA metabolism	transcription factor	.	Protein ariadne-1 homolog; ARI-1; Ubiquitin-conjugating enzyme E2-binding protein 1; UbcH7-binding protein; UbcM4-interacting protein; HHARI; H7-AP2; Monocyte protein 6; MOP-6	Os03g0233500 [Oryza sativa (japonica cultivar-group)] IBR domain containing protein, expressed [Oryza sativa (japonica cultivar-group)] Os03g0233500 [Oryza sativa (japonica cultivar-group)]	up (1.4700,1.6300)			1	0	0	1
SCJFRZ2014D06.g	GS1_AS_12278_15378	DNA metabolism	transcription factor	Stress	Water stress	induced		up (3.7500,3.0200)		1	0	0	1
SCJFLR1013E10.g	GS1_SS_03567_07008	DNA metabolism	transcription factor	.	RING finger and CHY zinc finger domain-containing protein 1; Zinc finger protein 363; CH-rich-interacting match with PLAG1; Androgen receptor N-terminal-interacting protein; p53-induced RING-H2 protein; hPirh2; RING finger protein 199	unknown [Zea mays]			up (3.9200,5.3100)	1	0	0	1
SCEPRT2048G05.g	GS1_SS_12965_16894	DNA metabolism	transcription factor	Transcription Factor	NAM	NAC		up (2.7000,6.2900)	up (2.9300,6.3600)	2	0	0	2
SCSGFL1078F07.g	GS1_SS_20499_17914	Flavonoid and anthocyanin metabolism	Reductase	Secondary metabolism	TPA: putative anthocyanidin reductase	hypothetical protein LOC100191338 [Zea mays] unknown [Zea mays]		down (-4.0500,-4.0700)		0	1	0	1
SCBFRZ2017F11.g	GS1_SS_14694_03476	Flavonoid and anthocyanin metabolism	Reductase	.	Putative dihydroflavonol-4-reductase; DFR; Dihydrokaempferol 4-reductase	unknown [Zea mays]		up (2.5700,4.1800)	up (5.0600,6.2700)	2	0	0	2

SCACFL5031E06.g	GS1_SS_12320_13147	Hormone biosynthesis	monooxygenase	.	Thiol-specific monooxygenase (Flavin-dependent monooxygenase)	unknown [Zea mays]			down (-4.0000,-4.8000)	0	1	0	1
SCSFRT2070A10.g	GS1_SS_13887_16812	Hormone biosynthesis	oxidoreductase	Hormone biosynthesis	Giberellin	oxidases			down (-3.0300,-2.8500)	0	1	0	1
SCCCCL7C03G05.g	GS1_SS_21236_03669	Hormone biosynthesis	oxygenase	.	Gibberellin 2-beta-dioxygenase; Gibberellin 2-beta-hydroxylase; Gibberellin 2-oxidase; GA 2-oxidase	hypothetical protein OsI_003721 [Oryza sativa (indica cultivar-group)]		up (5.9000,3.3200)	up (3.9500,4.3100)	2	0	0	2
SCCCCL7C03G05.g	GS1_AS_21236_03670	Hormone biosynthesis	oxygenase	.	Gibberellin 2-beta-dioxygenase; Gibberellin 2-beta-hydroxylase; Gibberellin 2-oxidase; GA 2-oxidase	hypothetical protein OsI_003721 [Oryza sativa (indica cultivar-group)]		up (3.0800,3.5000)		1	0	0	1
SCVPRZ2041G12.g	GS1_SS_06726_11141	Hormone biosynthesis	synthase	.	Probable indole-3-acetic acid-amido synthetase GH3.8; Auxin-responsive GH3-like protein 8; OsGH3-8 Probable indole-3-acetic acid-amido synthetase GH3.8; Auxin-responsive GH3-like protein 8; OsGH3-8	indole-3-acetic acid amido synthetase [Zea mays]		up (5.5300,3.2200)		1	0	0	1
SCAGFL8013F03.g	GS1_AS_25999_17044	Hormone biosynthesis	synthase	Hormone biosynthesis	Giberellin	synthases			down (-3.3600,-3.9200)	0	1	0	1
SCAGFL8013F03.g	GS1_SS_25999_17043	Hormone biosynthesis	synthase	Hormone biosynthesis	Giberellin	synthases			down (-3.2200,-3.6400)	0	1	0	1
SCCCCL3080B09.b	GS1_SS_05864_20064	Hormone biosynthesis	synthase	Transporters	ABC transporter (ABC_tran)	unknown [Zea mays]			up (8.1400,7.9100)	1	0	0	1
SCCCCL3002B05.b	GS1_SS_05762_18529	Hormone biosynthesis	synthase	Hormone related	auxin	auxin-responsive GH3 family protein			up (3.7900,4.2000)	1	0	0	1
SCCCST1002F06.g	GS1_SS_24272_20208	Light harvesting	chlorophyll binding	Carbohydrate metabolism	chlorophyll a/b binding protein (CAB)	unknown [Zea mays]			down (-4.4200,-6.6400)	0	1	0	1
SCEZLR1031G02.g	GS1_SS_10424_20078	Light harvesting	chlorophyll binding	Carbohydrate metabolism	chlorophyll a/b binding protein (CAB)	unknown [Zea mays]			down (-4.6800,-5.9100)	0	1	0	1
SCJLST1022G06.g	GS1_SS_17806_20038	Light harvesting	chlorophyll binding	Carbohydrate metabolism	chlorophyll a/b binding protein (CAB)	photosystem II subunit29 [Zea mays] Chlorophyll a/b-binding protein CP29 precursor [Zea mays] unknown [Zea mays]			down (-4.4700,-5.5300)	0	1	0	1
SCBGLR1114G09.g	GS1_SS_08436_17742	Light harvesting	others	Photosynthesis	(Q41048) Oxygen-evolving enhancer protein 3-1, chloroplast precursor (OEE3) (16 kDa subunit of oxyge	Water stress repressed			down (-4.6700,-6.9700)	0	1	0	1
SCJFAD1013H02.g	GS1_SS_17135_14149	Light harvesting	photosystem I subunit	.	.	unknown [Zea mays] unknown [Zea mays]		down (-2.5900,-4.5900)	down (-4.3300,-5.8800)	0	2	0	2
SCQGLR1085G01.g	GS1_SS_25636_20097	Light harvesting	photosystem I subunit	Photosynthesis	(Q00327) Photosystem I reaction center subunit V, chloroplast precursor (PSI-G) (Photosystem I 9 kDa	unknown [Zea mays]			down (-5.2400,-6.8900)	0	1	0	1
SCQGLR1085G01.g	GS1_AS_25636_20096	Light harvesting	photosystem I subunit	Photosynthesis	(Q00327) Photosystem I reaction center subunit V, chloroplast precursor (PSI-G) (Photosystem I 9 kDa	unknown [Zea mays]			down (-4.2400,-3.9900)	0	1	0	1
SCQGLR2025B12.g	GS1_SS_07917_21846	Light harvesting	PSI reaction center	Photosynthesis	(P23993) Photosystem I reaction center subunit XI, chloroplast precursor (PSI-L) (PSI subunit V)	Water stress repressed			down (-4.2100,-4.8800)	0	1	0	1
SCUTLR2008B06.g	GS1_SS_25626_19632	Light harvesting	PSI reaction center	Photosynthesis	(Q84PB5) Photosystem I protein-like protein	Water stress repressed			down (-3.8100,-4.3900)	0	1	0	1
SCSBSD2029H02.g	GS1_SS_26118_19348	Light harvesting	rubredoxin	Bioenergetics	Ferredoxin-1, chloroplastic; Ferredoxin I; Fd I;	sp|P27787|FER1_MAIZE Ferredoxin I, chloroplast precursor (Fd I) pir||T03286 ferredoxin [2Fe-2S] - maize gb|AAA33459.1| ferredoxin gb|AAA33460.1| ferredoxin prf||1907324B ferredoxin:ISOTYPE=I			down (-3.6900,-7.4400)	0	1	0	1
SCSGAM2078C07.g	GS1_AS_05162_06150	Light harvesting	rubredoxin	.	Ferredoxin	putative ferredoxin [Zea mays] unknown [Zea mays]		down (-2.9100,-2.9400)	down (-5.9300,-6.4400)	0	2	0	2
SCRFHR1006G03.g	GS1_SS_04426_12879	Light harvesting	uncharacterized	.	Chemocyanin precursor (Basic blue protein) (Plantacyanin)	unknown [Zea mays]			up (4.2100,3.6400)	1	0	0	1
SCSFLR2031F07.g	GS1_AS_15904_05746	Light harvesting	Uncharacterized	.	PsbP-like protein 1, chloroplastic; PsbP-related thylakoid lumenal protein 2; OEC23-like protein 4;	unknown [Zea mays]		up (2.8900,2.6900)	up (3.7200,3.8100)	2	0	0	2
SCUTSB1076D12.g	GS1_SS_17469_11499	Light harvesting	uncharacterized	.	Thylakoid lumenal protein At1g03610, chloroplastic;	unknown [Zea mays]			down (-3.9900,-5.4900)	0	1	0	1
SCRFFL5043E04.g	GS1_SS_23199_16513	Light harvesting	Uncharacterized	Photosynthesis	(Q7YJV0) PSII T protein	PSII T-protein [Vitis vinifera]			down (-2.9700,-3.5200)	0	1	0	1

SCVPLR2005B02.g	GS1_SS_09568_10302	Light harvesting	Uncharacterized	.	PsbP domain-containing protein 1, chloroplastic; PsbP-related thylakoid lumenal protein 1; OEC23-like protein 3;	unknown [Zea mays]		down (-2.5600,-2.6200)	down (-3.1600,-3.0200)	0	2	0	2
SCRLAM1006E01.g	GS1_SS_03023_14908	Light harvesting	Uncharacterized	Stress	.	repressed		down (-3.7400,-3.0900)	down (-4.5300,-5.1700)	0	2	0	2
SCEZSD1083H01.g	GS1_SS_25828_08824	Lipid metabolism	acyltransferase	.	3-ketoacyl-CoA synthase 11; KCS-11; Very long-chain fatty acid condensing enzyme 11; VLCFA condensing enzyme 11	unknown [Zea mays]			up (3.1600,3.8400)	1	0	0	1
SCCCCL7038A10.g	GS1_AS_26014_04520	Lipid metabolism	acyltransferase	.	1-acylglycerophosphocholine O-acyltransferase 1; Acyltransferase-like 2	unknown [Zea mays]			up (3.6000,3.7000)	1	0	0	1
SCEPSB1136F09.g	GS1_SS_21332_12694	Lipid metabolism	acyltransferase	.	Glycerol-3-phosphate acyltransferase 1; AtGPAT1	hypothetical protein OsI_002910 [Oryza sativa (indica cultivar-group)]			up (6.5600,4.6000)	1	0	0	1
SCRLFL1011A02.g	GS1_SS_19918_18280	Lipid metabolism	acyltransferase	Lipid, fatty-acid and isoprenoid metabolism	.	unknown [Zea mays]		up (5.0200,4.2700)	up (5.4600,4.5900)	2	0	0	2
SCEZAM2058C09.g	GS1_SS_04238_10821	Lipid metabolism	acyltransferase	.	2-acylglycerol O-acyltransferase 1; Monoacylglycerol O-acyltransferase 1; Acyl CoA:monoacylglycerol acyltransferase 1; MGAT1	hypothetical protein OsI_021982 [Oryza sativa (indica cultivar-group)]			up (5.2000,4.2000)	1	0	0	1
SCEZFL5089C05.g	GS1_SS_23691_13712	Lipid metabolism	Acyltransferases	.	Glycerol-3-phosphate acyltransferase 1; AtGPAT1	hypothetical protein OsI_001483 [Oryza sativa (indica cultivar-group)] hypothetical protein OsJ_001387 [Oryza sativa (japonica cultivar-group)]			up (5.9600,5.3000)	1	0	0	1
SCVPAM1059B02.g	GS1_SS_03228_16743	Lipid metabolism	dehydrogenase	Hormone biosynthesis	jasmonic acid	Omega-6 fatty acid desaturase			down (-3.1000,-4.0400)	0	1	0	1
SCJFRT1005C01.g	GS1_SS_19690_19234	Lipid metabolism	dehydrogenase	Lipid, fatty-acid and isoprenoid metabolism	Putative 3-hydroxybutyryl-CoA dehydrogenase	unknown [Zea mays]			up (2.9100,3.5100)	1	0	0	1
SCSGHR1072E12.g	GS1_SS_20554_02815	Lipid metabolism	hydrolase	.	Abhydrolase domain-containing protein 6-A	unknown [Zea mays]			up (4.5700,5.0500)	1	0	0	1
SCJFRT1009B01.g	GS1_SS_12614_15929	Lipid metabolism	hydrolase	Carbohydrate metabolism	patatin-like protein	unknown [Zea mays]		down (-2.6100,-3.8800)		0	1	0	1
SCQSRT1035D04.g	GS1_SS_12740_19911	Lipid metabolism	ligase	Lipid, fatty-acid and isoprenoid metabolism	Long-chain-fatty-acid-CoA ligase 5 (EC 6.2.1.3) (Long-chain acyl-CoA synthetase 5) (LACS 5	unknown [Zea mays]			up (4.0700,3.4600)	1	0	0	1
SCCCLR2004B05.g	GS1_SS_11262_13810	Lipid metabolism	lipase	.	Non-specific lipid-transfer protein 2; nsLTP2; 7 kDa lipid transfer protein;	unknown [Zea mays]			down (-3.5500,-3.8500)	0	1	0	1
SCCCRZ2C03B01.g	GS1_SS_22871_13852	Lipid metabolism	lipase	.	Anther-specific proline-rich protein APG; Protein CEX	unknown [Zea mays]			up (3.2300,4.5300)	1	0	0	1
SCJFRT1007H07.g	GS1_SS_20498_19027	Lipid metabolism	lipoxygenase	Hormone biosynthesis	jasmonic acid	Lipoxygenase			down (-3.1100,-6.3100)	0	1	0	1
SCBFRZ2045A12.g	GS1_SS_14710_13413	Lipid metabolism	methyltransferase	.	Phosphoethanolamine N-methyltransferase 1; PEAMT 1; AtNMT1	S-adenosyl-L-methionine: phosphoethanolamine N-methyltransferase [Zea mays]			down (-4.7200,-5.2100)	0	1	0	1
SCCCRT1002E01.g	GS1_SS_08457_17376	Lipid metabolism	others	Lipid, fatty-acid and isoprenoid metabolism	Putative stearoyl-acyl carrier protein desaturase	unknown [Zea mays]			up (2.9100,6.2700)	1	0	0	1
SCCCRT1001E01.g	GS1_SS_21695_20802	Lipid metabolism	others	Hormone biosynthesis	jasmonic acid	Lipoxygenase		down (-4.2700,-6.7100)		0	1	0	1
SCCCRT1001E01.g	GS1_AS_21695_20801	Lipid metabolism	others	Hormone biosynthesis	jasmonic acid	Lipoxygenase		down (-1.9300,-2.8500)		0	1	0	1
SCSBAM1085B06.g	GS1_SS_15769_16971	Lipid metabolism	oxidoreductase	lipid metabolism	Omega-3 fatty acid desaturase, chloroplastic;	Omega-3 fatty acid desaturase		down (-2.6600,-4.3600)		0	1	0	1
SCCCLR1024H09.g	GS1_SS_05820_20315	Lipid metabolism	Reductase	Lipid, fatty-acid and isoprenoid metabolism	Enoyl-[acyl-carrier protein] reductase [NADH], chloroplast precursor (EC 1.3.1.9) (NADH-dep	unknown [Zea mays]		down (-2.8600,-4.1000)		0	1	0	1
SCACRZ3109F05.g	GS1_SS_15295_16602	Lipid metabolism	synthase	Lipid, fatty-acid and isoprenoid metabolism	.	unknown [Zea mays]			down (-3.6500,-4.2400)	0	1	0	1
SCRFAM2070B12.g	GS1_SS_04825_11625	Lipid metabolism	synthase	.	Cyclopropane-fatty-acyl-phospholipid synthase; Cyclopropane fatty acid synthase; CFA synthase Cyclopropane-fatty-acyl-phospholipid synthase; Cyclopropane fatty acid synthase; CFA synthase	Os12g0267200 [Oryza sativa (japonica cultivar-group)] cyclopropane fatty acid synthase, putative, expressed [Oryza sativa (japonica cultivar-group)] Os12g0267200 [Oryza sativa (japonica cultivar-group)]		up (3.3900,6.9200)	up (5.1800,5.8600)	2	0	0	2
SCAGAM2016B02.g	GS1_SS_02699_02548	Lipid metabolism	synthase	.	Dehydrodolichyl diphosphate synthase 2 (Dedol-PP synthase 2)	unknown [Zea mays]		down (-2.9900,-4.1900)		0	1	0	1
SCVPLR2005H09.g	GS1_SS_11666_17875	Lipid metabolism	synthase	Lipid, fatty-acid and isoprenoid metabolism	3-oxoacyl-[acyl-carrier-protein] synthase I	hypothetical protein LOC100193451 [Zea mays] unknown [Zea mays]			down (-4.9900,-4.7700)	0	1	0	1

SCCCLR1C07E01.g	GS1_SS_00496_16485	Lipid metabolism	transferase	Lipid, fatty-acid and isoprenoid metabolism	putative sulfolipid synthase	unknown [Zea mays]	down (-2.9200,-1.6700)			0	1	0	1
SCMCLR1123C11.g	GS1_SS_10812_04628	Lipid metabolism	translocon	.	Transmembrane protein 56-B	hypothetical protein LOC100194195 [Zea mays] unknown [Zea mays]			up (3.1800,3.9600)	1	0	0	1
SCUTLR1037G02.g	GS1_SS_24077_16084	Maintenance of genetic material	DNA methylase	Chromatin regulation	chromomethylase CMT3 (cytosine methyltransferase)	DNA (cytosine-5)-methyltransferase 3; Chromomethylase 3; DNA methyltransferase 105 DNA methyltransferase 105 [Zea mays]			down (-5.5700,-5.6900)	0	1	0	1
SCQGAM1048F02.g	GS1_SS_02913_06742	Maintenance of genetic material	Helicase	.	DEAD-box ATP-dependent RNA helicase 25	Os01g0618400 [Oryza sativa (japonica cultivar-group)] DEAD-box ATP-dependent RNA helicase 25 putative RNA helicase [Oryza sativa Japonica Group] Os01g0618400 [Oryza sativa (japonica cultivar-group)]		down (-4.6100,-4.4000)	down (-4.8400,-4.1900)	0	2	0	2
SCVPRT2075A05.g	GS1_SS_05292_13126	Maintenance of genetic material	Replication / Helicase	.	.	hypothetical protein OsJ_032804 [Oryza sativa (japonica cultivar-group)]		down (-2.8100,-5.0300)		0	1	0	1
SCACLR1036A01.g	GS1_SS_25218_11290	Maintenance of genetic material	Ribossomal Protein	.	H/ACA ribonucleoprotein complex subunit 2-like protein; Nhp2-like protein	unknown [Zea mays]		down (-2.5300,-3.3200)		0	1	0	1
SCEQLR1007G09.g	GS1_SS_09074_09344	Maintenance of genetic material	SSB	.	Single-stranded DNA-binding protein; SSB; Helix-destabilizing protein	unknown [Zea mays]		down (-2.8200,-3.8500)		0	1	0	1
SCCCCL5004A02.g	GS1_SS_05548_12185	Maintenance of genetic material		.	Transcription factor bHLH47 OS=Arabidopsis thaliana GN=BHLH47 PE=2 SV=1	unknown [Zea mays]		up (4.0700,3.4300)		1	0	0	1
SCCCST1008B08.g	GS1_SS_03268_18427	Maintenance of genetic material		Nucleic acid metabolism	mRNA processing	Probable splicing factor 3B subunit 5 (SF3b5) (Pre-mRNA splicing factor SF3b 10 kDa subunit		down (-4.7600,-7.7500)		0	1	0	1
SCBFRZ2050A05.g	GS1_SS_23457_16388	Maintenance of genetic material		Nucleic acid metabolism	regulation of transcription, DNA-dependent	Probable WRKY transcription factor 19 (WRKY DNA-binding protein 19)		down (-2.2100,-3.6400)		0	1	0	1
SCMCRT2087B02.g	GS1_SS_07198_13237	Modification and protein degradation	cysteine protease inhibitors	.	Cysteine proteinase inhibitor 8; Oryzacystatin-8; Oryzacystatin VIII; OC-VIII;	putative cystatin [Zea mays] TPA: putative cystatin [Zea mays] putative cystatin [Zea mays] unknown [Zea mays]			up (6.1200,5.2400)	1	0	0	1
SCCCCL4005G05.g	GS1_SS_18344_01636	Modification and protein degradation	histone	.	Small heat shock protein, chloroplastic; Heat shock protein 26.6;	heat shock protein26 [Zea mays] heat shock protein 26 [Zea mays] unknown [Zea mays] unknown [Zea mays] heat shock protein 26			up (2.8700,6.3900)	1	0	0	1
SCCCLB1023F09.g	GS1_SS_23196_15971	Modification and protein degradation	peptidase	Protein metabolism	Cathepsin B (Fragment)	unknown [Zea mays]			up (4.0900,4.9300)	1	0	0	1
SCMCRZ3068D09.g	GS1_SS_16077_11938	Modification and protein degradation	ubiquitin	.	FAS-associated factor 2-A; UBX domain-containing protein 8-A	unknown [Zea mays]			down (-4.5200,-3.7500)	0	1	0	1
SCACST3159D04.g	GS1_SS_18393_15001	Nucleotide metabolism	dehydrogenase	Nucleic acid metabolism	Nucleotide metabolism	Inosine monophosphate dehydrogenase			up (3.4600,4.7700)	1	0	0	1
SCSGLV1007A08.g	GS1_SS_26112_06519	Nucleotide metabolism	hydrolase	.	Guanosine-3',5'-bis(diphosphate) 3'-pyrophosphohydrolase; Penta-phosphate guanosine-3'-pyrophosphohydrolase; (ppGpp)ase	unknown [Zea mays]		up (2.3900,3.0000)		1	0	0	1
SCCCCL4003D09.g	GS1_SS_05986_00413	Nucleotide metabolism	hydrolase	.	Probable AMP deaminase	hypothetical protein OsI_026471 [Oryza sativa (indica cultivar-group)]			up (3.6900,3.3100)	1	0	0	1
SCJFFL3C07G04.g	GS1_SS_22696_12722	Nucleotide metabolism	kinase/phosphatase	.	Uridylate kinase; UK; Uridine monophosphate kinase; UMP kinase; UMP/CMP kinase	unknown [Zea mays]		up (2.5300,3.0200)	up (3.2700,3.2000)	2	0	0	2
SCCCRZ2C03C09.g	GS1_SS_03914_01548	Nucleotide metabolism	others	.	Probable AMP deaminase	Os05g0349200 [Oryza sativa (japonica cultivar-group)] Os05g0349200 [Oryza sativa (japonica cultivar-group)]			down (-3.7100,-3.9800)	0	1	0	1
SCJFHR1C05B12.b	GS1_SS_08020_12067	Nucleotide metabolism	Reductase	.	.	.		up (7.1400,6.9300)	up (5.9500,6.9600)	2	0	0	2
SCSFLR2024H10.g	GS1_SS_11606_14655	Nucleotide metabolism	Transferase family	Nucleic acid metabolism	regulation of transcription, DNA-dependent	Transcription factor IIIC102		down (-3.2600,-3.3400)		0	1	0	1
SCQGST1034G07.g	GS1_SS_23697_16335	Nucleotide metabolism	Transporters	Nucleic acid metabolism	regulation of transcription, DNA-dependent	Glycine-rich protein 2		down (-2.2900,-3.3200)		0	1	0	1

SCEZRT2015F08.g	GS1_SS_19147_05948	Others	acetyl-CoA synthetase	.	Fatty acyl-CoA synthetase; Long-chain-fatty-acid--CoA synthetase 1; Long chain fatty acyl coenzyme A-synthetase 1; LC-FACS 1	Os01g0655800 [Oryza sativa (japonica cultivar-group)] Os01g0655800 [Oryza sativa (japonica cultivar-group)]		up (2.1200,2.3900)		1	0	0	1
SCCCST2003C12.g	GS1_SS_08764_21659	Others	Alcohol dehydrogenaseCell wall	Carbohydrate metabolism	Pentose and Glucuronate interconversions	Alcohol dehydrogenase(NADP+) EC 1.1.1.2			up (3.6500,3.4500)	1	0	0	1
SCCCCL2001H01.b	GS1_SS_05710_02918	Others	amidotransferase	.	Stem-specific protein TSJT1	expressed protein [Oryza sativa (japonica cultivar-group)] Stem-specific protein TSJT1, putative, expressed [Oryza sativa (japonica cultivar-group)]			up (2.8600,6.2500)	1	0	0	1
SCCCLR1075B06.g	GS1_SS_10234_14797	Others	bacteria	Photosynthesis	(Q40070) Photosystem II 10 kDa polypeptide, chloroplast precursor	putative Photosystem II 10 kDa polypeptide, chloroplast precursor [Oryza sativa Japonica Group]		up (8.2300,5.4000)		1	0	0	1
SCCCLR1075B06.g	GS1_AS_10234_14796	Others	bacteria	Photosynthesis	(Q40070) Photosystem II 10 kDa polypeptide, chloroplast precursor	putative Photosystem II 10 kDa polypeptide, chloroplast precursor [Oryza sativa Japonica Group]		up (3.1500,3.7500)		1	0	0	1
SCAGAM2126B01.g	GS1_SS_03766_02179	Others	chaperon	.	Probable mitochondrial chaperone bcs1 (BCS1-like protein)	putative AAA-type ATPase [Oryza sativa (japonica cultivar-group)] hypothetical protein OsJ_018939 [Oryza sativa (japonica cultivar-group)]			up (4.6300,4.3400)	1	0	0	1
SCQGHR1013B08.g	GS1_SS_07579_19807	Others	copper ion binding	Bioenergetics	Blue copper protein;	dbj|BAD03078.1| putative blue copper binding protein [Oryza sativa (japonica cultivar-group)]		down (-2.5900,-4.0500)		0	1	0	1
SCEPRZ1008H10.g	GS1_SS_21614_21828	Others	cytochrome	Stress	Cytochrome P450	Cyp only			up (3.0800,3.3300)	1	0	0	1
SCJLRT1022A05.g	GS1_SS_31358_16707	Others	cytochrome P450 family protein	Stress	Cytochrome P450	Cyp only		down (-2.4400,-3.1700)		0	1	0	1
SCEQRT1025C04.g	GS1_SS_11924_18787	Others	cytochrome P450 family protein	Stress	Cytochrome P450	Cyp only			up (6.2600,5.8700)	1	0	0	1
SCRFAD1024F09.g	GS1_SS_11822_15203	Others	cytochrome P450 family protein	Stress	Cytochrome P450	Cyp only			up (3.6700,5.1100)	1	0	0	1
SCEPSD1071E01.g	GS1_SS_25562_18811	Others	cytochrome P450 family protein	Stress	cytochrome P450	CYP81B1			up (4.1700,3.1200)	1	0	0	1
SCSBSD2029E08.g	GS1_SS_25567_05330	Others	cytochrome P450 family protein	.	Tyrosine N-monooxygenase; Cytochrome P450Tyr; Cytochrome P450 79A1	unknown [Zea mays]			up (6.2800,5.4600)	1	0	0	1
SCCCLB1C06G11.g	GS1_SS_08946_07668	Others	cytochrome P450 family protein	.	O-methyltransferase ZRP4 (OMT)	hypothetical protein OsI_035164 [Oryza sativa (indica cultivar-group)]		down (-2.9300,-3.2800)		0	1	0	1
SCJFRZ2030D03.g	GS1_SS_14939_21284	Others	cytochrome P450 family proteinCell wall	Stress	Cytochrome P450	Cyp only			up (4.7300,4.9000)	1	0	0	1
SCVPRZ3030D01.g	GS1_SS_18238_16749	Others	cytochrome P450 family proteinCell wall	Stress	Cytochrome P450	Cyp only		down (-3.2300,-3.2600)		0	1	0	1
SCCCLR2003C05.g	GS1_SS_10887_09804	Others	cytochrome P450 family proteinCell wall	.	Cytochrome P450 87A3	OSIGBa0111L12.4 [Oryza sativa (indica cultivar-group)]		down (-6.8400,-6.0600)		0	1	0	1
SCSFRT2067G11.g	GS1_SS_13860_18733	Others	cytochrome P450 family proteinCell wall	Development	ripening	Cytochrome P450 71A1 (EC 1.14.-.-) (CYPLXXIA1) (ARP-2)			down (-3.7500,-3.8200)	0	1	0	1
SCBFLR1005H06.g	GS1_SS_16572_16695	Others	cytochrome P450 family proteinCell wall	Stress	Cytochrome P450	Cyp only		down (-5.8300,-4.6500)		0	1	0	1
SCCCCL3003H04.b	GS1_SS_05809_16045	Others	cytochrome P450 family proteinCell wall	Stress	Cytochrome P450	Cyp only			up (3.7100,4.2500)	1	0	0	1
SCQGLR1087A09.g	GS1_SS_10900_18569	Others	dehydrogenase	Secondary metabolism	steroid 5 alpha-reductase 2-like; H5AR gene; steroid 5 alpha-reductase 2 like dbj|BAC30537.1| unnamed protein product dbj|BAC35871.1| unnamed protein product	Os04g0576800 [Oryza sativa (japonica cultivar-group)] OSJNBa0020J04.7 [Oryza sativa (japonica cultivar-group)] Os04g0576800 [Oryza sativa (japonica cultivar-group)] hypothetical protein OsI_016479 [Oryza sativa (indica cultivar-group)] hypothetical p			up (3.9600,3.8600)	1	0	0	1

SCQGAM2108C06.g	GS1_SS_04628_10900	Others	dehydrogenase	.	Lipoamide acyltransferase component of branched-chain alpha-keto acid dehydrogenase complex, mitochondrial; Dihydrolipoyllysine-residue (2-methylpropanoyl)transferase; Dihydrolipoamide branched chain transacylase; BCKAD E2 subunit; E2;	unknown [Zea mays]			up (5.5800,4.9800)	1	0	0	1
SCEQLR1092B06.g	GS1_SS_00037_14514	Others	electron carrier	.	Rubredoxin 1; Rd-1	unknown [Zea mays]			down (-4.0100,-5.1200)	0	1	0	1
SCJFRT2054F10.b	GS1_SS_13329_08859	Others	Flavonoides	.	3-ketoacyl-CoA synthase 11; KCS-11; Very long-chain fatty acid condensing enzyme 11; VLCFA condensing enzyme 11	unknown [Zea mays]			up (3.9500,5.1000)	1	0	0	1
SCSGAD1006B03.g	GS1_SS_21331_10483	Others	Glicosil-transferase	Cell wall	glucosyltransferase	unknown [Zea mays]		down (-2.7200,-4.9800)		0	1	0	1
SCJLST1020H07.g	GS1_SS_01085_16738	Others	Glycosyltransferase	Cell wall	Xyloglucan endotransglycosylase/hydrolase (XTH)	xyloglucan endo-transglycosylase/hydrolase [Zea mays] xyloglucan endo-transglycosylase/hydrolase [Zea mays]		down (-2.3100,-5.4300)	down (-3.8200,-5.9600)	0	2	0	2
SCRUFL3067H08.g	GS1_SS_22685_20741	Others	glycosyltransferases	Secondary metabolism	glucosyltransferase-9	unknown [Zea mays]			down (-6.1200,-7.0200)	0	1	0	1
SCCCLR2004G11.g	GS1_SS_02400_12430	Others	hydrolase	.	Putative lipase ROG1; Revertant of glycogen synthase kinase mutation protein 1	unknown [Zea mays]		up (1.9200,2.7100)		1	0	0	1
SCRURT3063D02.g	GS1_SS_25008_05086	Others	hydrolase	.	.	hypothetical protein OsJ_017815 [Oryza sativa (japonica cultivar-group)]			down (-3.2500,-3.8600)	0	1	0	1
SCACLR1057A09.g	GS1_SS_21649_10221	Others	hydrolase	.	Putative copper-transporting ATPase 3	putative copper-exporting ATPase [Sorghum bicolor]		up (2.2600,5.1600)		1	0	0	1
SCCCSD1003E06.g	GS1_SS_25375_14237	Others	inhibitor	Pathogenicity	Protease inhibitor	thaumatin			up (7.4900,4.1700)	1	0	0	1
SCCCSD2001E05.g	GS1_SS_25669_19204	Others	inhibitor	Pathogenicity	protease inhibitor	Pathogenesis-related protein 5/thaumatin related			up (3.1600,5.9900)	1	0	0	1
SCJLLR1102C07.g	GS1_SS_10657_10822	Others	ion binding	.	PsbP-like protein 2, chloroplastic;	hypothetical protein OsJ_009954 [Oryza sativa (japonica cultivar-group)]			down (-4.2700,-6.5100)	0	1	0	1
SCEQLB1064H09.g	GS1_SS_09110_10359	Others	isomerase	.	Probable FKBP-type peptidyl-prolyl cis-trans isomerase 2, chloroplast precursor (PPIase) (Rotamase)	hypothetical protein OsI_029064 [Oryza sativa (indica cultivar-group)]		down (-3.2700,-7.3300)	down (-7.4400,-8.6100)	0	2	0	2
SCJLST1021E11.g	GS1_SS_02949_13718	Others	isomerase	Transcription Factor	AP2/EREBP	Tiny			down (-4.1800,-4.6100)	0	1	0	1
SCMCSB1115D10.g	GS1_AS_24110_17334	Others	kinase/phosphatase	Transporters	ABC1 family (ABC1)	Os09g0250700 [Oryza sativa (japonica cultivar-group)] ABC1 family protein-like [Oryza sativa Japonica Group] Os09g0250700 [Oryza sativa (japonica cultivar-group)]		up (3.6200,3.3200)		1	0	0	1
SCEPSD2008F06.g	GS1_SS_25775_17841	Others	kinase/phosphatase	Carbohydrate metabolism	Carbon fixation	Phosphoribulokinase EC 2.7.1.19		down (-3.2400,-4.3200)	down (-5.4600,-8.1100)	0	2	0	2
SCCCLR1C01D07.g	GS1_SS_00808_05136	Others	kinase/phosphatase	.	Soluble inorganic pyrophosphatase (Pyrophosphate phospho-hydrolase) (PPase) Soluble inorganic pyrophosphatase (Pyrophosphate phospho-hydrolase) (PPase)	Os02g0704900 [Oryza sativa (japonica cultivar-group)] Soluble inorganic pyrophosphatase (Pyrophosphate phospho-hydrolase) (PPase) Soluble inorganic pyrophosphatase (Pyrophosphate phospho-hydrolase) (PPase) inorganic pyrophosphatase [Oryza sativa Japo			down (-2.8700,-4.2300)	0	1	0	1
SCQGRZ3010C10.g	GS1_SS_08191_08901	Others	kinase/phosphatase	.	Protein phosphatase PTC7 homolog	unknown [Zea mays]		down (-2.5400,-2.5500)	down (-3.8100,-4.3400)	0	2	0	2
SCQSHR1024G09.g	GS1_SS_08308_10681	Others	kinase/phosphatase	Receptors	Receptor Ser/Thr kinase	cane S-receptor-43			down (-3.2000,-5.9000)	0	1	0	1
SCJLRT1017C01.g	GS1_SS_12493_03509	Others	kinase/phosphatase	.	CTD small phosphatase-like protein 2; CTDSP-like 2	hypothetical protein OsI_019593 [Oryza sativa (indica cultivar-group)]		up (2.6600,2.7500)		1	0	0	1
SCMCSD2061D05.g	GS1_SS_25565_17459	Others	kinase/phosphatase	Protein kinase	undefined unclassified	caneUPK-46 (CBL-interacting protein kinase - CIPK)			up (3.2400,7.4800)	1	0	0	1
SCMCST1051F08.g	GS1_SS_15048_17158	Others	kinase/phosphatase	Protein phosphatase	.	Tyrosine Specific Protein Phosphatases (PTP)		down (-2.6800,-4.3100)		0	1	0	1
SCCCLB1001E04.g	GS1_SS_03025_20848	Others	kinase/phosphatase	Carbohydrate metabolism	vegetative storage protein (acid phosphatase)	unknown [Zea mays]		down (-3.1400,-5.8600)	down (-4.9900,-4.6300)	0	2	0	2

SCMCSB1115D10.g	GS1_SS_24110_17333	Others	kinase/phosphatase	Transporters	ABC1 family (ABC1)	Os09g0250700 [Oryza sativa (japonica cultivar-group)] ABC1 family protein-like [Oryza sativa Japonica Group] Os09g0250700 [Oryza sativa (japonica cultivar-group)]		up (2.2000,3.0300)		1	0	0	1
SCJLHR1028E06.g	GS1_SS_08176_19578	Others	methyltransferaseCell wall	Hormone biosynthesis	Jasmonate	carboxyl methyltransferase		up (3.1900,4.5500)	up (4.9100,4.9200)	2	0	0	2
SCAGSB1089E04.g	GS1_AS_21758_03851	Others	NAD dependent epimerase/dehydratase familyputative dihydroflavonol-4-reductase or Cinnamoyl-coA reductase (CCR)Cell wall	.	Dihydroflavonol-4-reductase; DFR; Dihydrokaempferol 4-reductase	unknown [Zea mays]			down (-2.8000,-3.6700)	0	1	0	1
SCAGSB1089E04.g	GS1_SS_21758_03852	Others	NAD dependent epimerase/dehydratase familyputative dihydroflavonol-4-reductase or Cinnamoyl-coA reductase (CCR)Cell wall	.	Dihydroflavonol-4-reductase; DFR; Dihydrokaempferol 4-reductase	unknown [Zea mays]			down (-3.3500,-4.0400)	0	1	0	1
SCCCAM1C01F03.g	GS1_SS_01828_19341	Others	O-methyltransferase (OMT)Cell wall	Inositol	others	inositol o-methyltransferase		down (-5.2200,-5.6900)	down (-4.8000,-6.5500)	0	2	0	2
SCAGAM2018H09.g	GS1_SS_03687_06940	Others	others	.	Senescence-associated protein DIN1	unknown [Zea mays]			down (-5.7800,-3.6800)	0	1	0	1
SCCCRT2C08E12.g	GS1_SS_07813_09729	Others	others	.	UPF0497 membrane protein At4g03540	unknown [Zea mays]			up (4.6800,4.4800)	1	0	0	1
SCBFSD1037G05.g	GS1_SS_25364_10919	Others	others	.	Desiccation-related protein PCC13-62;	unknown [Zea mays]			up (8.0400,7.3100)	1	0	0	1
SCCCCL3004A08.b	GS1_SS_05812_10057	Others	others	.	Early nodulin 93; N-93	unknown [Zea mays]			up (4.9700,4.0600)	1	0	0	1
SCBFSD1034C06.g	GS1_SS_26225_05201	Others	oxidase	.	Polyphenol oxidase, chloroplastic; PPO; Catechol oxidase;	polyphenol oxidase [Triticum aestivum]		up (2.2700,5.5400)	up (7.0300,6.4000)	2	0	0	2
SCQGLR2025H03.g	GS1_SS_08026_15032	Others	oxidoreductase	Photosynthesis	(Q55087) Geranylgeranyl hydrogenase	unknown [Zea mays]			down (-4.1900,-5.3100)	0	1	0	1
SCEQRT1033E11.g	GS1_SS_00870_06146	Others	oxygenase	.	2-nitropropane dioxygenase; 2-NPD; Nitroalkane oxidase;	hypothetical protein LOC100191301 [Zea mays] unknown [Zea mays]		up (2.4400,2.8600)		1	0	0	1
SCEPSD1005H05.g	GS1_SS_26217_01541	Others	oxygenase	.	Hyoscyamine 6-dioxygenase; Hyoscyamine 6-beta-hydroxylase	ids2 [Hordeum vulgare subsp. vulgare]			down (-4.2300,-5.4500)	0	1	0	1
SCJFRT1010D11.g	GS1_SS_12203_08593	Others	oxygenase	.	.	unknown [Zea mays]			up (3.2200,4.0200)	1	0	0	1
SCQGRT3044B11.g	GS1_SS_26250_03614	Others	peptidase	.	Serine carboxypeptidase-like 18;	unknown [Zea mays]		up (2.5400,3.6700)		1	0	0	1
SCQGLV1018H09.g	GS1_SS_26173_02086	Others	protease	.	Aspartic proteinase-like protein 2;	unknown [Zea mays]			up (3.2800,3.4300)	1	0	0	1
SCEPAM1051H04.g	GS1_SS_02069_13254	Others	protein-protein interaction	.	Thylakoid membrane phosphoprotein 14 kDa, chloroplastic;	Os01g0761000 [Oryza sativa (japonica cultivar-group)] Os01g0761000 [Oryza sativa (japonica cultivar-group)]			down (-2.7300,-3.4900)	0	1	0	1
SCQSRT2034D09.g	GS1_SS_06805_06778	Others	Redutase	.	Short-chain dehydrogenase/reductase 2; AtSDR2	unknown [Zea mays] unknown [Zea mays]			up (3.9600,3.9600)	1	0	0	1
SCJFHR1C01B11.g	GS1_SS_07914_11991	Others	Ribosomal protein	.	30S ribosomal protein S5, chloroplastic;	unknown [Zea mays]		down (-2.7900,-2.7700)	down (-3.3300,-3.1700)	0	2	0	2
SCQSLR1018D09.g	GS1_SS_03133_00292	Others	serine and sphingolipid biosynthesis	.	Probable serine incorporator	unknown [Zea mays]			down (-3.6200,-2.8100)	0	1	0	1
SCCCSD2003H04.g	GS1_SS_25824_10702	Others	storage protein	Library-specific	By domain	Tryp_alpha_amyl		down (-3.6700,-3.1700)		0	1	0	1
SCCCLB1025A11.g	GS1_SS_03575_06174	Others	Structural protein	.	Remorin; DNA-binding protein	hypothetical protein LOC100191495 [Zea mays] unknown [Zea mays]			down (-4.8300,-4.6900)	0	1	0	1
SCEPRZ3047C10.g	GS1_SS_15387_20175	Others	synthase	Carbohydrate metabolism	Photosynthesis	ATP synthase delta chain (atp-delta-thylakoid ATPase)			down (-3.8600,-4.1000)	0	1	0	1
SCQGFL4071E04.g	GS1_SS_19281_20818	Others	synthase	Carbohydrate metabolism	N metabolism	glutamine synthetase			down (-4.0700,-7.3100)	0	1	0	1
SCCCFL6002C02.g	GS1_SS_22161_12137	Others	synthase	.	Bifunctional 3' phosphoadenosine 5-phosphosulfate synthetase 2; PAPS synthetase 2; PAPSS 2; Sulfurylase kinase 2; SK 2; SK2; Includes: Sulfate adenylyltransferase; Sulfate adenylate transferase; SAT; ATP-sulfurylase; Includes: Adenylyl-sulfate kinas	unknown [Zea mays]		down (-2.1600,-2.7500)	down (-3.1600,-4.3900)	0	2	0	2

SCCCAM1C09G11.g	GS1_SS_22185_09919	Others	transcription factor	.	Homeobox-leucine zipper protein HOX29; Homeodomain transcription factor HOX29; HD-ZIP protein HOX29; OsHox29	TPA: TPA_inf: class III HD-Zip III protein HB8 [Zea mays]		up (4.3400,3.1100)		1	0	0	1
SCQSAD1056E03.b	GS1_SS_00803_18820	Others	Transferase family	Secondary metabolism	hydroxycinnamoyl CoA quinate transferase	unknown [Zea mays]		up (2.5800,4.2700)	up (3.5000,4.9200)	2	0	0	2
SCBFSD2037A07.g	GS1_SS_25527_20012	Others	Transferase family	Photosynthesis	(P31171) Prenyl transferase (EC 2.5.1.-)	Os05g0582300 [Oryza sativa (japonica cultivar-group)] putative polyprenyl diphosphate synthase [Oryza sativa (japonica cultivar-group)] putative polyprenyl diphosphate synthase [Oryza sativa (japonica cultivar-group)] Os05g0582300 [Oryza sativa (japo			down (-4.5800,-5.0600)	0	1	0	1
SCSBHR1052F05.g	GS1_SS_08432_01884	Others	Transferase family proteinCell wall	.	Uncharacterized acetyltransferase At3g50280	unknown [Zea mays]			down (-4.1400,-4.3700)	0	1	0	1
SCVPRZ2044C07.g	GS1_SS_15182_02683	Others	Transporters	.	Putative copper-transporting ATPase 3	unknown [Zea mays]			up (4.3400,4.7000)	1	0	0	1
SCQSAD1055C01.g	GS1_SS_24242_09754	Others	ubiquitination	.	Probable ubiquitin carrier protein E2 25	hypothetical protein LOC100194284 [Zea mays] unknown [Zea mays]		up (2.3500,3.9500)	up (4.0700,3.7800)	2	0	0	2
SCQGRT1041D10.g	GS1_SS_31422_20960	Others	Uncharacterized	Protein metabolism	ClpC ATPase	ATP-dependent Clp protease ATP-binding subunit precursor [Oryza sativa (indica cultivar-group)]			up (5.0500,5.0500)	1	0	0	1
SCCCRZ1003C02.g	GS1_SS_11060_08972	Others	Uncharacterized	.	.	unknown [Zea mays]		down (-1.9800,-3.0100)		0	1	0	1
SCRFLR1012G08.g	GS1_SS_10963_08117	Others	uncharacterized	.	Pentatricopeptide repeat-containing protein At1g31920	hypothetical protein OsJ_007760 [Oryza sativa (japonica cultivar-group)]			down (-6.2400,-7.5900)	0	1	0	1
SCRUFL1114B10.b	GS1_SS_23953_10139	Others	uncharacterized	.	Uncharacterized protein ycf39; ORF319	unknown [Zea mays]			down (-3.6700,-5.9800)	0	1	0	1
SCQGRT1041G07.g	GS1_SS_12676_06000	Others	Uncharacterized	.	F-box/Kelch-repeat protein At2g02870	hypothetical protein OsJ_033090 [Oryza sativa (japonica cultivar-group)]			up (3.6400,4.9500)	1	0	0	1
SCRFLR1012G08.g	GS1_AS_10963_08118	Others	uncharacterized	.	Pentatricopeptide repeat-containing protein At1g31920	hypothetical protein OsJ_007760 [Oryza sativa (japonica cultivar-group)]			down (-4.9200,-5.0500)	0	1	0	1
SCCCLR1066D07.g	GS1_SS_10164_00356	Others	Uncharacterized	.	.	unknown [Zea mays]			down (-2.9400,-5.8500)	0	1	0	1
SCRUFL1016D08.g	GS1_SS_22085_02163	Others	Uncharacterized	.	Uncharacterized protein At4g01050	unknown [Zea mays]		down (-2.0500,-2.8900)	down (-3.3900,-3.6400)	0	2	0	2
SCJFAM1065H10.g	GS1_SS_02355_01484	Others	uncharacterized	.	Protein brittle-1, chloroplastic/amyloplastic;	Os08g0520000 [Oryza sativa (japonica cultivar-group)] putative mitochondrial energy transfer protein [Oryza sativa Japonica Group] putative mitochondrial energy transfer protein [Oryza sativa Japonica Group] Os08g0520000 [Oryza sativa (japonica culti			up (3.6700,3.6200)	1	0	0	1
SCRFLR1055G04.g	GS1_SS_03206_04746	Others	zinc-binding protein	.	E3 ubiquitin-protein ligase CHFR; Checkpoint with forkhead and RING finger domains protein	Os11g0175500 [Oryza sativa (japonica cultivar-group)] At1g47570 [Oryza sativa (japonica cultivar-group)] Zinc finger, C3HC4 type family protein, expressed [Oryza sativa (japonica cultivar-group)] Os11g0175500 [Oryza sativa (japonica cultivar-group)]		up (2.6500,2.7800)	up (4.6800,5.2900)	2	0	0	2
SCEPLR1051B04.g	GS1_SS_13317_12515	Others		Cell wall	Fasciclin-like arabinogalactan protein	unknown [Zea mays]		down (-2.3100,-4.1700)		0	1	0	1
SCRFLB1056D05.g	GS1_AS_24218_01518	Others		.	E3 ubiquitin-protein ligase MARCH8; Membrane-associated RING finger protein 8; Membrane-associated RING-CH protein VIII; MARCH-VIII; RING finger protein 178; Cellular modulator of immune recognition; c-MIR	unknown [Zea mays]		up (6.9300,2.7900)	up (3.7300,6.2000)	2	0	0	2
SCCCST1003D04.g	GS1_SS_04384_10182	Others		Stress	drought and cold response	early-responsive to dehydration stress protein (erd3-like)			up (3.0500,3.2100)	1	0	0	1

SCCCRZ1004G06.g	GS1_SS_14493_11369	Others		.	Probable protein arginine N-methyltransferase 4.2	Os06g0142800 [Oryza sativa (japonica cultivar-group)] Probable protein arginine N-methyltransferase 4.2 protein arginine N-methyltransferase protein -like [Oryza sativa Japonica Group] Os06g0142800 [Oryza sativa (japonica cultivar-group)] hypothetica		down (-2.9300,-4.2800)		0	1	0	1
SCRLSB1044E01.g	GS1_SS_21526_15823	Others		Nucleic acid metabolism	Nucleotide metabolism	AAA-type ATPase family protein		up (3.3300,3.0400)	up (2.7400,4.0600)	2	0	0	2
SCQSLR1089D04.g	GS1_SS_07456_16851	Others		Carbohydrate metabolism	.	Isocitrate dehydrogenase EC 1.1.1.42			down (-6.6900,-5.8300)	0	1	0	1
SCEZLB1008G11.g	GS1_SS_17362_08695	Others		.	Protein IQ-DOMAIN 31	unknown [Zea mays]		down (-3.8900,-4.3800)	down (-4.7000,-5.8000)	0	2	0	2
SCCCLB1C06D11.g	GS1_SS_04112_13280	Others		.	FK506-binding protein 39 kDa (Peptidyl-prolyl cis-trans isomerase) (PPIase) (Rotamase)	unknown [Zea mays]		down (-2.8100,-3.4800)		0	1	0	1
SCEZLB1013B06.g	GS1_SS_17150_17496	Others		Nitrogen metabolism	Myosin heavy chain	With LRR domain		down (-2.7900,-4.4300)		0	1	0	1
SCEZRZ1012E01.g	GS1_SS_23177_13976	Others		.	UPF0557 protein C10orf119 homolog	Os01g0166800 [Oryza sativa (japonica cultivar-group)] unknown protein [Oryza sativa Japonica Group] Os01g0166800 [Oryza sativa (japonica cultivar-group)]			down (-3.6000,-4.6700)	0	1	0	1
SCAGLR1021F10.g	GS1_SS_07364_07335	Others		.	.	unknown [Zea mays]		down (-3.6900,-5.5200)	down (-5.9800,-4.8300)	0	2	0	2
SCAGLR2011E12.g	GS1_SS_11370_11622	Others		.	Uncharacterized protein At2g39795, mitochondrial precursor	unknown [Zea mays]	down (-1.5000,-1.2100)	down (-3.6100,-5.0100)		0	2	0	2
SCACLR1128D05.g	GS1_SS_09754_10442	Others		.	.	unknown [Zea mays]			down (-5.1400,-3.9100)	0	1	0	1
SCRURT2013H02.g	GS1_SS_13840_03777	Others		.	UPF0301 protein CT0663	unknown [Zea mays]		down (-2.7800,-4.7100)		0	1	0	1
SCSBFL1039F08.g	GS1_SS_20403_19091	Others		Transcription Factor	NAM (no apical meristem)	YZ1 (CBS domain-containing protein)		up (7.2400,4.5800)	up (3.9900,5.2300)	2	0	0	2
SCCCCL3001C02.b	GS1_SS_05720_06578	Others		.	Oleosin Zm-I (Oleosin 16 kDa) (Lipid body-associated major protein) (Lipid body-associated protein L3)	unknown [Zea mays] unknown [Zea mays] unknown [Zea mays]		up (6.5700,3.2000)		1	0	0	1
SCBGSB1025F02.g	GS1_SS_24192_03990	Others		Cell wall	beta-1,3-glucanase Glycosyl hydrolases family 17	unknown [Zea mays]		down (-4.4300,-5.1200)	down (-4.8800,-5.0100)	0	2	0	2
SCCCCL5004D07.g	GS1_SS_24563_04818	Others		.	.	unknown [Zea mays]			down (-5.8200,-5.2100)	0	1	0	1
SCEQAM1044G07.g	GS1_SS_24070_09926	Others		.	Pentatricopeptide repeat-containing protein At1g74750	hypothetical protein OsI_027103 [Oryza sativa (indica cultivar-group)]		down (-2.1900,-5.0200)		0	1	0	1
SCAGAM2126G09.g	GS1_SS_20067_01031	Oxidative phosphorylation	dehydrogenase	.	.	unknown [Zea mays]		down (-2.4000,-3.8300)	down (-4.7300,-5.8700)	0	2	0	2
SCRLFL3007A08.g	GS1_SS_22596_08519	Oxidative phosphorylation	dehydrogenase	.	NAD(P)H-quinone oxidoreductase subunit L; NAD(P)H dehydrogenase I subunit L; NDH-1 subunit L; NDH-L	unknown [Zea mays]			down (-3.6600,-5.5800)	0	1	0	1
SCAGLR2018C03.g	GS1_SS_08201_08606	Oxidative phosphorylation	others	.	Blue copper protein;	unknown [Zea mays]	down (-2.3900,-1.6400)		down (-2.7300,-3.6900)	0	2	0	2
SCBFSD1037F06.g	GS1_SS_25361_09160	Oxidative phosphorylation	others	.	.	unknown [Zea mays]			down (-4.1000,-5.2900)	0	1	0	1
SCEPSD2005D11.g	GS1_SS_25416_00588	Oxidative phosphorylation	oxidoreductase	.	NAD(P)H-quinone oxidoreductase subunit M, chloroplastic; NAD(P)H dehydrogenase I subunit M; NDH-1 subunit M; NDH-M; NAD(P)H-quinone oxidoreductase subunit M, chloroplastic; NAD(P)H dehydrogenase I subunit M; NDH-1 subunit M; NDH-M;	unknown [Zea mays]			down (-3.6100,-5.1700)	0	1	0	1
SCSGAD1009E08.g	GS1_SS_01426_21879	Oxidative phosphorylation	synthase	Carbohydrate metabolism	Photosynthesis	ATP synthase delta chain (atp-delta-thylakoid ATPase)			down (-2.7400,-5.5100)	0	1	0	1
SCSGAD1009E08.b	GS1_SS_01448_08558	Oxidative phosphorylation	synthase	.	ATP synthase delta chain, chloroplastic; F-ATPase delta chain;	ATP synthase delta chain, chloroplastic; F-ATPase delta chain; H(+)-transporting ATP synthase [Sorghum bicolor]			down (-3.7000,-6.1000)	0	1	0	1
SCJLRT2051G07.g	GS1_SS_25693_18364	Pathogen Resistance	antimicrobial	Pathogenicity	Protease inhibitors	Thaumatin		up (2.5400,4.6200)	up (6.8900,4.5800)	2	0	0	2
SCVPLR1028H01.g	GS1_SS_08400_14365	Pathogen Resistance	hydrolase	Pathogenicity	Protease inhibitor	thaumatin		up (5.6000,4.4900)		1	0	0	1
SCCCST1004F07.g	GS1_SS_17517_11707	Pathogen Resistance	induced by biotic and abiotic stress and hormone	Cell wall	chitinase	hypothetical protein OsI_028911 [Oryza sativa (indica cultivar-group)]		down (-2.0000,-4.1100)		0	1	0	1
SCCCCL3003E07.b	GS1_SS_06601_18767	Pathogen Resistance	induced by biotic and abiotic stress and hormone	Cell wall	chitinase	chitinase B [Saccharum officinarum]		up (2.5400,4.7200)	up (7.0700,7.8300)	2	0	0	2

SCQSRT1036B10.g	GS1_SS_25808_17599	Pathogen Resistance	induzida por stress biotico e abiotico e por hormonios	Cell wall	chitinase	Chitinase EC 3.2.1.14			up (5.0500,3.5200)	1	0	0	1
SCCCRT3001A02.g	GS1_SS_24414_20650	Pathogen Resistance	induzida por stress biotico e abiotico e por hormonios	Cell wall	chitinase	Endochitinase A precursor (Seed chitinase A)			up (7.7700,6.3400)	1	0	0	1
SCBGRT1050A08.g	GS1_SS_25394_21770	Pathogen Resistance	induzida por stress biotico e abiotico e por hormonios	Cell wall	chitinase	hypothetical protein OsI_033349 [Oryza sativa (indica cultivar-group)]			up (5.0500,4.2300)	1	0	0	1
SCCCLR1079G06.g	GS1_SS_14231_14239	Pathogen Resistance	kinase/phosphatase	Protein kinase	putative RLCK	caneRLCK-AI2			down (-2.9300,-6.0900)	0	1	0	1
SCCCLR1024E01.g	GS1_SS_18865_10717	Pathogen Resistance	others	.	.	unknown [Zea mays]			down (-3.3900,-4.7300)	0	1	0	1
SCRFSD2023E07.g	GS1_SS_41756_17921	Pathogen Resistance	peptidase	Pathogenicity	R-genes transduction	PR		up (3.5200,4.1800)	up (4.5800,4.7600)	2	0	0	2
SCEPSD1007C11.g	GS1_SS_25637_17940	Pathogen Resistance	uncharacterized	Pathogenicity	R-gene transduction	PR			up (6.9300,6.6400)	1	0	0	1
SCVPRT2073B08.g	GS1_SS_11812_14889	Pathogen Resistance	Uncharacterized	Pathogenicity	R-genes transduction	PR			up (7.9200,6.1400)	1	0	0	1
SCUTFL3074E04.g	GS1_SS_38621_17997	Pathogen Resistance	Uncharacterized	Carbohydrate metabolism	.	beta-glucosidase aggregating factor [Sorghum bicolor]			down (-3.6900,-4.3300)	0	1	0	1
SCEQRT1024H10.g	GS1_SS_13487_20028	Pathogen Resistance	uncharacterized	Carbohydrate metabolism	Glyoxylate cycle	malate synthase			up (7.9800,7.3200)	1	0	0	1
SCQSRT1035D12.g	GS1_SS_24797_18959	Pathogen Resistance	uncharacterized	Pathogenicity	Protease inhibitors	Thaumatin		up (2.6000,4.3300)	up (8.3600,6.7000)	2	0	0	2
SCSFST3077H01.g	GS1_SS_25786_18600	Photosynthesis	Clorofila	Carbohydrate metabolism	chlorophyll a/b binding protein (CAB)	hypothetical protein LOC100191715 [Zea mays] unknown [Zea mays]			down (-3.7800,-6.6700)	0	1	0	1
SCEQHR1082D05.g	GS1_SS_07606_16073	Porphyrin and chlorophyll metabolism	chelatase	Secondary metabolism	protoporphyrin IX magnesium chelatase-like protein	hypothetical protein OsI_011049 [Oryza sativa (indica cultivar-group)]			down (-3.5600,-4.6300)	0	1	0	1
SCSFCL6068B09.g	GS1_SS_24292_16622	Porphyrin and chlorophyll metabolism	decarboxylase	Secondary metabolism	Uroporphyrinogen-III decarboxylase	Os01g0622300 [Oryza sativa (japonica cultivar-group)] putative uroporphyrinogen decarboxylase [Oryza sativa Japonica Group] Os01g0622300 [Oryza sativa (japonica cultivar-group)]		down (-2.8700,-3.0800)		0	1	0	1
SCEQHR1080F03.g	GS1_SS_24189_12326	Porphyrin and chlorophyll metabolism	decarboxylase	.	Uroporphyrinogen decarboxylase, chloroplastic; URO-D; UPD;	unknown [Zea mays]		down (-2.4800,-3.8100)		0	1	0	1
SCACLR2014H06.g	GS1_SS_03006_13423	Porphyrin and chlorophyll metabolism	kinase/phosphatase	.	Probable NAD kinase 2, chloroplastic;	hypothetical protein OsJ_031917 [Oryza sativa (japonica cultivar-group)]		up (2.0600,3.1800)		1	0	0	1
SCEZSB1090G04.g	GS1_SS_19469_21692	Porphyrin and chlorophyll metabolism	ligase	Photosynthesis	(P72772) Magnesium-chelatase subunit chlD (Mg-protoporphyrin IX chelatase) (Mg-chelatase subunit D)	Os03g0811100 [Oryza sativa (japonica cultivar-group)] putative magnesium chelatase [Oryza sativa (japonica cultivar-group)] magnesium chelatase ATPase subunit D family protein, expressed [Oryza sativa (japonica cultivar-group)] Os03g0811100 [Oryza sa		down (-2.7400,-3.7700)	down (-3.5000,-4.8100)	0	2	0	2
SCACLR1057H07.g	GS1_SS_04477_20876	Porphyrin and chlorophyll metabolism	others	Photosynthesis	(Q94H64) Putative chelatase subunit	unknown [Zea mays]		down (-3.9200,-7.3600)	down (-7.6200,-8.4000)	0	2	0	2
SCACLR1057H07.g	GS1_AS_04477_20877	Porphyrin and chlorophyll metabolism	others	Photosynthesis	(Q94H64) Putative chelatase subunit	unknown [Zea mays]		down (-1.3200,-3.2800)		0	1	0	1
SCACSB1118D08.g	GS1_SS_20850_18571	Porphyrin and chlorophyll metabolism	others	Photosynthesis	(Q7XZG7) Putative magnesium chelatase subunit chlD	hypothetical protein OsI_013532 [Oryza sativa (indica cultivar-group)] hypothetical protein OsJ_012501 [Oryza sativa (japonica cultivar-group)]		down (-2.8000,-3.9700)		0	1	0	1
SCCCLB1021E12.g	GS1_SS_16038_21347	Porphyrin and chlorophyll metabolism	oxidase	Protein metabolism	Protoporphyrinogen IX oxidase (EC 1.3.3.4)	unknown [Zea mays]		down (-3.4700,-4.0800)	down (-4.4100,-4.8200)	0	2	0	2
SCCCLR1022F01.g	GS1_SS_20645_01924	Porphyrin and chlorophyll metabolism	Reductase	.	Carbonyl reductase 4	unknown [Zea mays]			up (3.1200,3.5600)	1	0	0	1
SCCCCL4005E12.g	GS1_SS_09119_19261	Protein metabolism	chaperon	Calcium metabolism	calmodulin-binding proteins	Chaperonin 10		down (-3.4400,-4.9400)		0	1	0	1
SCEQLB1068F08.g	GS1_SS_19091_11764	Protein metabolism	chaperon	.	Chaperone protein dnaJ 11, chloroplastic; AtJ11; AtDjC11;	hypothetical protein OsI_029130 [Oryza sativa (indica cultivar-group)]		up (2.7000,3.6200)		1	0	0	1
SCCCLR1070E06.g	GS1_SS_17503_14606	Protein metabolism	elongation factor	Protein metabolism	Eukaryotic translation initiation factor 5A-1/2 (eIF-5A 1/2) (eIF-4D)	hypothetical protein LOC100192991 [Zea mays] unknown [Zea mays]		down (-2.1900,-3.2800)		0	1	0	1
SCJFRT1060E10.g	GS1_SS_12310_06740	Protein metabolism	F-box	.	F-box/Kelch-repeat protein At1g74510	hypothetical protein OsJ_008102 [Oryza sativa (japonica cultivar-group)]			up (7.1000,5.7300)	1	0	0	1
SCEZLB1006D01.g	GS1_SS_09165_11095	Protein metabolism	F-box	.	F-box protein At2g26160	unnamed protein product [Vitis vinifera]			down (-4.2300,-6.5500)	0	1	0	1

SCSBAD1052A06.g	GS1_SS_01122_02959	Protein metabolism	isomerase	.	FKBP-type peptidyl-prolyl cis-trans isomerase 6, chloroplastic; FKBP20-2; PPIase; Rotamase;	unknown [Zea mays]		down (-3.8500,-3.5600)	down (-5.2800,-3.9700)	0	2	0	2
SCCCLB1002A09.g	GS1_SS_08831_18370	Protein metabolism	others	Ubiquitination	E2	unknown [Zea mays]		up (2.7600,3.2700)		1	0	0	1
SCCCRT1004E02.g	GS1_SS_18669_08783	Protein metabolism	others	.	.	unknown [Zea mays]		up (3.8900,4.3000)		1	0	0	1
SCACLR1126A09.g	GS1_SS_09611_07688	Protein metabolism	others	.	Translation initiation factor IF-3	hypothetical protein LOC100193325 [Zea mays] unknown [Zea mays]			down (-3.5500,-4.8900)	0	1	0	1
SCCCRZ2C04C11.g	GS1_SS_01867_08709	Protein metabolism	others	.	.	unknown [Zea mays]		up (3.2400,3.2000)		1	0	0	1
SCRFLB2060E04.g	GS1_SS_25725_08917	Protein metabolism	oxidoreductase	.	Nucleoredoxin	hypothetical protein LOC100192725 [Zea mays] unknown [Zea mays]		up (7.2400,2.9600)	up (4.9600,5.0200)	2	0	0	2
SCQSLB1049F06.g	GS1_SS_23959_20720	Protein metabolism	peptidase	Protein metabolism	Subtilisin-like serine protease	hypothetical protein OsI_028433 [Oryza sativa (indica cultivar-group)]			down (-3.2100,-4.1500)	0	1	0	1
SCCCRT2004D09.g	GS1_SS_12619_17993	Protein metabolism	peptidase	Protein metabolism	Putative serine carboxypeptidase	Serine carboxypeptidase family protein, expressed [Oryza sativa (japonica cultivar-group)]			down (-3.5500,-4.6600)	0	1	0	1
SCEZAM2033B11.g	GS1_SS_04193_03059	Protein metabolism	peptidase	.	Serine carboxypeptidase-like 26;	unknown [Zea mays]	down (-2.3100,-3.1500)			0	1	0	1
SCQGLR1062F08.g	GS1_AS_10809_04863	Protein metabolism	peptidase	.	ATP-dependent Clp protease ATP-binding subunit clpA homolog CD4B, chloroplastic;	hypothetical protein OsJ_014066 [Oryza sativa (japonica cultivar-group)]			up (3.3800,3.9200)	1	0	0	1
SCRFLR2038B01.g	GS1_SS_11907_11959	Protein metabolism	protease	.	Aspartic proteinase nepenthesin-2; Nepenthesin-II;	unknown [Zea mays]		up (2.8900,3.5100)	up (2.9500,3.3500)	2	0	0	2
SCJFRZ2009E12.g	GS1_SS_24924_16478	Protein metabolism	protease	Protein metabolism	subtilase family protein	hypothetical protein OsI_030760 [Oryza sativa (indica cultivar-group)]			down (-5.1200,-6.6600)	0	1	0	1
SCEPRZ3129G09.g	GS1_SS_19353_07185	Protein metabolism	release factor activity	.	Eukaryotic peptide chain release factor subunit 1-1 (Eukaryotic release factor 1-1) (eRF1-1) (Omnipotent suppressor protein 1 homolog 1) (SUP1 homolog 1)	unknown [Zea mays]		up (3.9000,2.9100)	up (4.2000,5.1900)	2	0	0	2
SCRUHR1076E02.g	GS1_SS_08097_18431	Protein metabolism	Ribosomal protein	Protein metabolism	50S ribosomal protein L24, chloroplast precursor (CL24)	unknown [Zea mays]		down (-3.6700,-3.4400)	down (-3.9200,-3.3600)	0	2	0	2
SCSGHR1066A02.g	GS1_SS_03000_19495	Protein metabolism	Ribosomal protein	Protein metabolism	50S ribosomal protein L15, chloroplast precursor (CL15) (Fragment)	unknown [Zea mays]		down (-3.4800,-3.9900)	down (-5.3800,-5.5300)	0	2	0	2
SCRFLR1034H12.g	GS1_SS_22510_03436	Protein metabolism	ribosomal protein	.	50S ribosomal protein L6, chloroplastic; Protein EMBRYO DEFECTIVE 2394;	unknown [Zea mays]			down (-2.8900,-3.3600)	0	1	0	1
SCCCCL5001D04.g	GS1_SS_07223_04063	Protein metabolism	RNA-binding protein	.	50S ribosomal protein L4, chloroplastic; CL4; R-protein L4;	unknown [Zea mays]			down (-3.8800,-4.1500)	0	1	0	1
SCCCLR1C01A11.g	GS1_SS_10566_01416	Protein metabolism	synthase	.	Tyrosyl-tRNA synthetase, cytoplasmic; Tyrosyl--tRNA ligase; TyrRS	unknown [Zea mays]	up (2.3300,1.9100)			1	0	0	1
SCEZLR1052E03.g	GS1_SS_10436_11125	Redox metabolism	electron carrier	.	Thioredoxin F-type 2, chloroplastic; Trx-F2;	unknown [Zea mays]			down (-3.8000,-3.3100)	0	1	0	1
SCQGST1034C05.g	GS1_SS_06748_18960	Redox metabolism	Multicopper oxidase	Carbohydrate metabolism	Cell wall	Pectinesterase EC 3.1.1.11		down (-2.2300,-4.1100)		0	1	0	1
SCUTST3084C11.g	GS1_SS_18949_03741	Redox metabolism	Multicopper oxidase	Cell wall	laccase-like protein	hypothetical protein LOC100193025 [Zea mays] unknown [Zea mays]			down (-4.6500,-4.8100)	0	1	0	1
SCQGLR2025B01.g	GS1_SS_03563_13355	Redox metabolism	Multicopper oxidase	Cell wall	laccase-like protein	unknown [Zea mays]		down (-3.7000,-5.9400)	down (-3.4200,-6.4300)	0	2	0	2
SCBGLR1096C09.g	GS1_SS_12233_00222	Redox metabolism	others	.	Peroxiredoxin Q, chloroplastic; Thioredoxin reductase;	unknown [Zea mays]		down (-2.6500,-4.5200)	down (-4.3100,-5.0100)	0	2	0	2
SCCCCL4011A06.g	GS1_SS_07348_08693	Redox metabolism	oxidoreductase	.	Probable aldehyde oxidase 2; AO-2	aldehyde oxidase-2 [Zea mays] aldehyde oxidase-2 [Zea mays]			down (-4.0300,-3.3900)	0	1	0	1
SCCCLR1076H02.g	GS1_SS_18560_04913	Redox metabolism	oxidoreductase	.	Probable NADP-dependent oxidoreductase P2	2-alkenal reductase [Hordeum vulgare subsp. vulgare]			down (-4.2000,-5.3700)	0	1	0	1
SCCCCL3120A03.g	GS1_SS_06792_07689	Redox metabolism	oxidoreductase	.	Probable NADP-dependent oxidoreductase P1	hypothetical protein OsI_034490 [Oryza sativa (indica cultivar-group)]		down (-6.7600,-3.9200)		0	1	0	1
SCQGSB1142G01.g	GS1_SS_21273_17968	Redox metabolism	oxidoreductase	Stress	Water stress	induced		down (-3.9900,-4.6000)		0	1	0	1
SCJLRT2050G12.g	GS1_SS_13486_05014	Redox metabolism	oxygenase	.	Uricase-2; Uricase II; Urate oxidase; Nodule-specific uricase	urate oxidase [Sorghum bicolor]		up (2.0100,5.2600)	up (6.0700,5.6500)	2	0	0	2
SCCCST3005E08.g	GS1_SS_04718_20859	Redox metabolism	peroxidase	Stress	response to oxidative stress	Peroxidase			down (-9.0600,-8.7500)	0	1	0	1
SCCCCL4008H04.g	GS1_SS_05420_14205	Redox metabolism	peroxidase	.	Peroxidase 56; Atperox P56; ATP33;	unknown [Zea mays]			up (3.5000,3.9200)	1	0	0	1

SCEQRT1028D12.g	GS1_SS_11752_04522	Redox metabolism	peroxidase	Library-specific	By domain	Peroxidases		down (-3.1400,-4.9800)		0	1	0	1
SCJFSB1012D05.g	GS1_SS_16908_14285	Redox metabolism	peroxidase	.	Putative L-ascorbate peroxidase 6; AtAPx08	hypothetical protein OsJ_026860 [Oryza sativa (japonica cultivar-group)]		down (-2.1100,-3.3800)		0	1	0	1
SCBFLR1083H12.g	GS1_SS_17326_20059	Redox metabolism	peroxidase	Stress	response to oxidative stress	Bacterial-induced peroxidase precursor			down (-3.5900,-4.6500)	0	1	0	1
SCCCCL3002F08.b	GS1_SS_12334_19734	Redox metabolism	peroxidaseCell wall	Stress	response to oxidative stress	Peroxidase		down (-4.7200,-6.9400)		0	1	0	1
SCBGHR1058E08.g	GS1_SS_07477_19258	Redox metabolism	peroxidaseCell wall	Stress	response to oxidative stress	Peroxidase precursor			down (-3.9000,-6.1200)	0	1	0	1
SCVPLB1020D03.g	GS1_SS_07881_07151	Redox metabolism	peroxidaseCell wall	.	Peroxidase 12; Atperox P12; PRXR6; ATP4a;	unknown [Zea mays]		up (2.3400,3.0800)	up (5.0800,3.1900)	2	0	0	2
SCSBSD1033E10.g	GS1_SS_25423_10062	Redox metabolism	peroxidaseCell wall	Library-specific	By domain	Peroxidases		up (6.8600,5.6300)	up (6.0800,7.1200)	2	0	0	2
SCCCAD1001C08.g	GS1_AS_00007_14860	Redox metabolism	peroxidaseCell wall	Stress	peroxidase	P7X			down (-3.0100,-3.3500)	0	1	0	1
SCCCAD1001C08.g	GS1_SS_00007_14861	Redox metabolism	peroxidaseCell wall	Stress	peroxidase	P7X			down (-3.9600,-4.0000)	0	1	0	1
SCEZRT2019F10.g	GS1_SS_06839_14194	Redox metabolism	peroxidaseCell wall	.	Peroxidase 1;	unknown [Zea mays]			down (-4.2400,-4.9900)	0	1	0	1
SCCCCL7037A10.g	GS1_SS_25248_14705	Redox metabolism	peroxidaseCell wall	Stress	Water stress	repressed		down (-3.1100,-3.6500)		0	1	0	1
SCBFRT1071H10.g	GS1_SS_00190_10768	Redox metabolism	Reductase	.	Putative tropinone reductase homolog At1g07440	unknown [Zea mays]		up (2.4900,3.1500)		1	0	0	1
SCBGLR1118D04.g	GS1_SS_01434_11119	Redox metabolism	synthase	.	Putative nitric oxide synthase	hypothetical protein OsJ_004896 [Oryza sativa (japonica cultivar-group)]		down (-2.8600,-4.3300)		0	1	0	1
SCJFRT1059B02.g	GS1_SS_12283_09183	Redox metabolism	transferase	.	Probable glutathione S-transferase GSTF1; GST-I	glutathione-S-transferase 19E50 [Triticum aestivum]			down (-3.0700,-3.9100)	0	1	0	1
SCCCCL4007F05.g	GS1_SS_12928_14208	Redox metabolism	transferase	.	Probable glutathione S-transferase GSTU6; 28 kDa cold-induced protein	unknown [Zea mays]			up (3.5900,4.0500)	1	0	0	1
SCEZRZ3098F04.g	GS1_SS_24818_03826	Redox metabolism	Transferase family	.	Probable glutathione S-transferase BZ2; Protein bronze-2	bronze-2 protein [Zea mays]			up (3.7900,4.1300)	1	0	0	1
SCQSRT1034G09.g	GS1_SS_18176_12363	Redox metabolism		.	Probable sarcosine oxidase	unknown [Zea mays]			up (3.6900,5.3100)	1	0	0	1
SCJFAM1067G04.b	GS1_SS_02449_14302	RNA metabolism	helicase	.	Putative helicase mug81 (Meiotically up-regulated gene 81 protein)	hypothetical protein OsJ_009528 [Oryza sativa (japonica cultivar-group)]		up (3.4900,3.8000)	up (4.2700,5.1800)	2	0	0	2
SCCCRZ1002D07.g	GS1_SS_14183_14981	RNA metabolism	hydrolase	Protein metabolism	putative CRS2-associated factor 1	unknown [Zea mays]		down (-2.0900,-2.5000)		0	1	0	1
SCJLRT1016G06.g	GS1_AS_12487_19040	RNA metabolism	nuclease	Stress	wound-induced	Ribonuclease		up (2.9900,3.2200)		1	0	0	1
SCJLRT1016G06.g	GS1_SS_12487_19039	RNA metabolism	nuclease	Stress	wound-induced	Ribonuclease		up (5.6500,3.5400)		1	0	0	1
SCSGRT2065E10.g	GS1_SS_07126_19446	RNA metabolism	nuclease	Nucleic acid metabolism	DNA catabolism	Histone-lysine N-methyltransferase, H3 lysine-9 specific 5 (EC 2.1.1.43) (Histone H3-K9 met		up (2.2100,3.9700)	up (6.5800,4.6100)	2	0	0	2
SCAGLR1043B12.g	GS1_SS_18184_19625	RNA metabolism	regulatory protein	Nucleic acid metabolism	mRNA processing	Putative histone H2 protein		down (-2.4800,-4.3500)		0	1	0	1
SCEZAM1081G05.g	GS1_SS_24478_05144	RNA metabolism	Ribosomal protein	.	U6 snRNA-associated Sm-like protein LSm6 U6 snRNA-associated Sm-like protein LSm6	Os04g0388900 [Oryza sativa (japonica cultivar-group)] Os04g0388900 [Oryza sativa (japonica cultivar-group)]		down (-3.8300,-3.2900)		0	1	0	1
SCCCCL5003D05.g	GS1_SS_08869_19374	RNA metabolism	rna binding protein	Nucleic acid metabolism	regulation of transcription, DNA-dependent	Small nuclear ribonucleoprotein F (snRNP-F) (Sm protein F) (Sm-F) (SmF)		down (-2.8600,-4.0500)		0	1	0	1
SCCCLR1022A08.g	GS1_SS_07068_09635	RNA metabolism	rna binding protein	.	H/ACA ribonucleoprotein complex subunit 4; Nucleolar protein NAP57 homolog; Nopp-140-associated protein of 57 kDa homolog; AtNAP57	Os07g0636000 [Oryza sativa (japonica cultivar-group)] putative centromere/microtubule binding protein [Oryza sativa Japonica Group] Os07g0636000 [Oryza sativa (japonica cultivar-group)] hypothetical protein OsI_026066 [Oryza sativa (indica cultivar-g		down (-2.7000,-4.8800)		0	1	0	1
SCBFLR1026B07.g	GS1_SS_24393_15654	RNA metabolism	rna binding protein	Nucleic acid metabolism	regulation of transcription, DNA-dependent	Transcriptional co-repressor LEUNIG		down (-2.6700,-4.4800)		0	1	0	1
SCEPLR1051G01.g	GS1_SS_14616_02256	RNA metabolism	rna binding protein	.	Glycine-rich RNA-binding protein 2, mitochondrial; AtGRP2;	unknown [Zea mays]		down (-3.3000,-5.3700)		0	1	0	1
SCBFLR1026B07.g	GS1_AS_24393_15653	RNA metabolism	rna binding protein	Nucleic acid metabolism	regulation of transcription, DNA-dependent	Transcriptional co-repressor LEUNIG		down (-2.8300,-3.4500)		0	1	0	1
SCEPRZ1010F12.g	GS1_SS_09748_13823	RNA metabolism	rna binding protein	.	Protein gar2	H0714H04.9 [Oryza sativa (indica cultivar-group)]		down (-2.3800,-4.5800)		0	1	0	1
SCCCFL5096E07.g	GS1_SS_23812_05352	RNA metabolism	rna binding protein	.	Chloroplastic group IIA intron splicing facilitator CRS1, chloroplastic; Protein CHLOROPLAST RNA SPLICING 1; Chloroplastic RNA splicing factor 1;	CRM family member 3 [Zea mays] CRM family member 3 [Zea mays]		down (-2.0900,-5.0700)		0	1	0	1
SCSBRZ3123G10.g	GS1_SS_22095_13084	RNA metabolism	RNA polymerase	.	RNA-dependent RNA polymerase 1 (Protein rdp1)	RNA-directed RNA polymerase 2 [Hordeum vulgare subsp. vulgare]			down (-4.7600,-4.2600)	0	1	0	1

SCCCLR1080D05.g	GS1_SS_10282_13917	RNA metabolism	RNA-binding protein	.	UDP-N-acetylglucosamine pyrophosphorylase	hypothetical protein OsJ_018784 [Oryza sativa (japonica cultivar-group)]		down (-1.9900,-3.3700)		0	1	0	1
SCCCRZ3002E05.g	GS1_SS_15205_03914	RNA metabolism	RNA-binding protein	.	Protein argonaute	Os04g0615700 [Oryza sativa (japonica cultivar-group)] OSJNBa0008M17.11 [Oryza sativa (japonica cultivar-group)] Os04g0615700 [Oryza sativa (japonica cultivar-group)]		up (1.9600,2.8400)		1	0	0	1
SCCCCL4009D08.g	GS1_SS_06081_19373	RNA metabolism	RNA-binding protein	G protein	beta	U3 small nucleolar ribonucleoprotein complex-associated protein		down (-2.3400,-4.3000)		0	1	0	1
SCBFRZ2049F01.g	GS1_SS_14740_20275	RNA metabolism	RNA-binding protein	Protein metabolism	Plastid ribosomal protein L11	unknown [Zea mays]			down (-3.1700,-3.9700)	0	1	0	1
SCBFRZ2017B05.g	GS1_SS_14687_04038	RNA metabolism	RNA-binding protein	.	30S ribosomal protein S17	unknown [Zea mays]		down (-2.0200,-3.6900)		0	1	0	1
SCJFLR1074E06.g	GS1_SS_04955_06554	RNA metabolism	RNA-binding protein	.	31 kDa ribonucleoprotein, chloroplastic;	hypothetical protein LOC100191381 [Zea mays] unknown [Zea mays]		down (-2.3000,-4.4000)		0	1	0	1
SCCCCL3080B11.g	GS1_SS_05851_17594	RNA metabolism	RNA-binding protein	Protein metabolism	50S ribosomal protein L28, chloroplast precursor	unknown [Zea mays]			down (-2.9400,-4.1300)	0	1	0	1
SCJFRZ2015C10.g	GS1_AS_04373_20546	RNA metabolism	transcription factor	Protein metabolism	Eukaryotic translation initiation factor 4E-1 (eIF4E-1) (eIF-4E-1) (mRNA cap-binding protein)	unknown [Zea mays]		up (3.2700,4.9300)	up (4.5100,4.3000)	2	0	0	2
SCEZHR1088C09.g	GS1_AS_06761_04380	RNA metabolism	Uncharacterized	.	Nucleolar complex protein 3 homolog; NOC3 protein homolog; NOC3-like protein; Nucleolar complex-associated protein 3-like protein	hypothetical protein OsI_022289 [Oryza sativa (indica cultivar-group)]			up (4.0100,3.0500)	1	0	0	1
SCJFLR2036D12.g	GS1_SS_23119_08244	RNA metabolism	Uncharacterized	.	50S ribosomal protein L35, chloroplastic; CL35;	prpl35-1 protein [Zea mays] putative plastid ribosomal protein L35 [Zea mays] putative plastid ribosomal protein L35 [Zea mays] unknown [Zea mays]			down (-4.0200,-5.1100)	0	1	0	1
SCCCCL6002B03.g	GS1_SS_06306_15240	Senescence and cell death	oxidoreductase	Secondary metabolism	ethylene-forming-enzyme-like dioxygenase-like protein	Os06g0178700 [Oryza sativa (japonica cultivar-group)] putative iron/ascorbate-dependent oxidoreductase [Oryza sativa Japonica Group] Os06g0178700 [Oryza sativa (japonica cultivar-group)] hypothetical protein OsI_021128 [Oryza sativa (indica cultivar-			up (5.3800,3.9600)	1	0	0	1
SCQGLR1085F11.g	GS1_SS_11161_19643	Signal Transduction	dehydrin	Stress	drought-induced	Dehydrin		up (7.0500,2.7900)	up (4.3600,5.6900)	2	0	0	2
SCCCRZ2002A07.g	GS1_SS_14585_14039	Signal Transduction	GTPase	Small GTPases	Rab	unknown [Zea mays]			down (-3.3400,-4.3900)	0	1	0	1
SCEPAM1024G02.g	GS1_SS_02026_18921	Signal Transduction	GTPase	Protein metabolism	GTP-binding protein typA (Tyrosine phosphorylated protein A)	hypothetical protein OsJ_006092 [Oryza sativa (japonica cultivar-group)]			down (-3.7100,-4.4800)	0	1	0	1
SCAGCL6013C08.g	GS1_SS_24814_09036	Signal Transduction	hydrolase	.	Barwin	unknown [Zea mays]	up (2.1600,2.5800)		up (5.0600,3.2900)	2	0	0	2
SCCCLR1001A03.g	GS1_SS_20259_21669	Signal Transduction	Internal signal transduction	Cell wall	LRR receptor-like serine/threonine-protein kinase ERECTA	caneErecta-1			down (-3.5300,-5.2400)	0	1	0	1
SCBGRT3012C05.g	GS1_SS_24459_15161	Signal Transduction	kinase receptor	Receptors	receptor Ser/Thr kinase	caneRLK lectin domain-8			up (3.3800,4.7700)	1	0	0	1
SCCCRZ1C01H05.g	GS1_SS_20070_19627	Signal Transduction	kinase receptor	Receptors	receptor Ser/Thr kinase	caneRLK-DVI8 (LRR)		down (-2.6400,-5.1200)	down (-3.3300,-5.1100)	0	2	0	2
SCCCAM1002H12.g	GS1_SS_26896_08853	Signal Transduction	kinase receptor	Receptors	receptor Ser/Thr kinase	caneLr10-1 (putative rust resistance kinase)			up (4.6700,4.9100)	1	0	0	1
SCCCLR2C02A04.g	GS1_SS_08002_15649	Signal Transduction	kinase receptor	Receptors	receptor Ser/Thr kinase	caneS receptor-19			down (-4.5600,-6.6300)	0	1	0	1
SCQGSB1083A03.g	GS1_SS_17025_15294	Signal Transduction	kinase/phosphatase	Protein kinase	Undefined- unclassified	caneUPK-53		up (3.8200,2.7800)		1	0	0	1
SCAGRT2038H05.g	GS1_SS_12827_15904	Signal Transduction	kinase/phosphatase	Receptors	Receptor Ser/Thr kinase	caneLRK10-1			up (5.0900,5.1200)	1	0	0	1
SCEZRZ1014A03.g	GS1_SS_20028_01063	Signal Transduction	kinase/phosphatase	.	Pantothenate kinase 2 (Pantothenic acid kinase 2)	unknown [Zea mays]		up (1.8300,3.3300)	up (3.6300,3.2700)	2	0	0	2
SCJFRZ2027C05.g	GS1_SS_14966_17859	Signal Transduction	kinase/phosphatase	Receptors	receptor Ser/Thr kinase	caneCLV1-7		down (-2.4400,-4.3500)	down (-3.9800,-5.3500)	0	2	0	2
SCJLLR1104D05.g	GS1_SS_10683_10646	Signal Transduction	kinase/phosphatase	.	Carboxymethylenebutenolidase homolog	unknown [Zea mays]			down (-3.1700,-4.0100)	0	1	0	1
SCJFRZ2011E02.g	GS1_SS_14834_04894	Signal Transduction	kinase/phosphatase	.	Inositol-tetrakisphosphate 1-kinase 2 (Inositol-triphosphate 5/6-kinase 2) (Inositol 1,3,4-trisphosphate 5/6-kinase 2) (Ins(1,3,4)P(3) 5/6-kinase 2) (AtItpk-2)	hypothetical protein LOC100194132 [Zea mays] unknown [Zea mays]			up (4.1000,4.8700)	1	0	0	1
SCJLRT2049F12.g	GS1_SS_07861_16087	Signal Transduction	kinase/phosphatase	Protein kinase	MAPK/MAPKK/MAPKKK	caneMAPK-21			up (4.1300,3.5600)	1	0	0	1
SCJFRZ3C07E06.g	GS1_SS_15918_20293	Signal Transduction	kinase/phosphatase	Receptors	receptor Ser/Thr kinase	caneRLK-DXV2			down (-3.9800,-3.7400)	0	1	0	1
SCCCLR2C01G05.g	GS1_SS_11287_03238	Signal Transduction	kinase/phosphatase	.	Putative receptor protein kinase TMK1;	SHR5-receptor-like kinase [Saccharum hybrid cultivar]			up (2.5600,3.3600)	1	0	0	1

SCCCST1003H06.g	GS1_SS_07789_21514	Signal Transduction	kinase/phosphatase	Receptors	receptor Ser/Thr kinase	caneRLK-DI2			up (4.3600,5.7000)	1	0	0	1
SCVPST1059E02.g	GS1_SS_18260_17408	Signal Transduction	kinase/phosphatase	Protein kinase	cdpk	Family 4.2.1 - Calcium Dependent Protein Kinase (CPK)			down (-2.9100,-4.7400)	0	1	0	1
SCBFRZ2048D04.g	GS1_SS_03974_02428	Signal Transduction	kinase/phosphatase	.	CBL-interacting protein kinase 14; OsCIPK14	unknown [Zea mays]	up (1.1100,2.5400)			1	0	0	1
SCEPRZ1011A11.g	GS1_SS_14297_03846	Signal Transduction	kinase/phosphatase	.	Protein phosphatase PTC7 homolog	Protein phosphatase 2C containing protein, expressed [Oryza sativa (japonica cultivar-group)]			down (-3.4300,-3.2200)	0	1	0	1
SCBFAM2025E08.g	GS1_SS_03837_15912	Signal Transduction	kinase/phosphatase	Protein kinase	RLCK	NAK		down (-2.7900,-3.8600)	down (-3.6200,-3.9800)	0	2	0	2
SCCCLR1C06C01.g	GS1_SS_16218_15589	Signal Transduction	kinase/phosphatase	Receptors	receptor Ser/Thr kinase	caneCLV1-1			up (5.0900,3.2900)	1	0	0	1
SCEQRT1029G10.g	GS1_SS_15800_21210	Signal Transduction	kinase/phosphatase	Receptors	receptor Ser/Thr kinase	caneRLK lectin domain-4		down (-2.5100,-4.9700)		0	1	0	1
SCEQRT3C04D05.g	GS1_SS_22120_10198	Signal Transduction	kinase/phosphatase	.	.	gene X-like protein [Sorghum bicolor]		up (4.9200,3.9600)	up (3.3000,3.7400)	2	0	0	2
SCCCHR1004D03.g	GS1_SS_15645_16608	Signal Transduction	kinase/phosphatase	Receptors	receptor Ser/Thr kinase	caneRLK-CII1		down (-2.5400,-3.2900)	down (-3.5500,-3.2700)	0	2	0	2
SCCCRZ2C04B09.g	GS1_SS_14990_16798	Signal Transduction	kinase/phosphatase	Protein phosphatase	serine/threonine PPM family	PP2A/Subunit B			down (-3.2600,-3.8500)	0	1	0	1
SCEZLB1009D04.g	GS1_SS_13925_19329	Signal Transduction	kinase/phosphatase	Cell cycle	.	hypothetical protein [Sorghum bicolor]		up (1.8600,3.8300)	up (3.8600,4.1400)	2	0	0	2
SCJFRT1010C12.g	GS1_SS_24135_18369	Signal Transduction	kinase/phosphatase	Protein kinase	putative RLCK	caneSIK putative protein-1		up (2.5400,3.3900)		1	0	0	1
SCJLRT1014G05.g	GS1_SS_12458_08039	Signal Transduction	kinase/phosphatase	.	Probable tyrosine-protein phosphatase At1g05000	unknown [Zea mays]	down (-1.1000,-2.2000)			0	1	0	1
SCBFRZ2050G04.g	GS1_SS_08070_01762	Signal Transduction	kinase/phosphatase	.	Mitogen-activated protein kinase 14 (MAP kinase 14)	mitogen activated protein kinase 6 [Zea mays] mitogen activated protein kinase 6 [Zea mays]			down (-2.8400,-5.1700)	0	1	0	1
SCEZRT2019B08.g	GS1_SS_13212_09134	Signal Transduction	kinase/phosphatase	.	SAC domain-containing protein 3; FIG4 homolog	hypothetical protein OsJ_009310 [Oryza sativa (japonica cultivar-group)]		up (2.9800,3.1300)		1	0	0	1
SCEQHR1081F09.g	GS1_SS_07593_16571	Signal Transduction	kinase/phosphatase	Receptors	Receptor Ser/Thr kinase-unclassified	caneURLK-22		down (-3.2100,-4.5100)		0	1	0	1
SCJLLR1103H01.g	GS1_SS_24575_18337	Signal Transduction	kinase/phosphatase	Carbohydrate metabolism	Galactose metabolism	Galactokinase EC 2.7.1.6			up (5.6300,5.0900)	1	0	0	1
SCQGSB1140C12.g	GS1_SS_21263_19003	Signal Transduction	kinase/phosphatase	Receptors	receptor Ser/Thr kinase	caneCR4-3		down (-2.5300,-3.6400)	down (-3.5300,-4.5600)	0	2	0	2
SCBFSB1045E02.g	GS1_SS_16720_16653	Signal Transduction	ligase	Lipid, fatty-acid and isoprenoid metabolism	putative RING zinc finger protein	unknown [Zea mays]		down (-2.7900,-5.0400)	down (-3.2600,-4.9300)	0	2	0	2
SCCCRZ2002B04.g	GS1_SS_08713_09224	Signal Transduction	lipase	.	.	unknown [Zea mays]			down (-3.0100,-3.1000)	0	1	0	1
SCEPAM1053C09.g	GS1_SS_20360_09819	Signal Transduction	metalloprotease	.	Speckle-type POZ protein; HIB homolog 1; Roadkill homolog 1 Speckle-type POZ protein; HIB homolog 1; PDX-1 C-terminal-interacting factor 1 Speckle-type POZ protein Speckle-type POZ protein	unknown [Zea mays]	down (-1.6400,-2.2800)			0	1	0	1
SCCCLR2C01F06.g	GS1_SS_11286_19433	Signal Transduction	others	Stress	wound-induced	wound-responsive family protein	down (-2.0200,-1.9300)		up (2.7400,6.3500)	1	1	0	2
SCCCCL4010F07.g	GS1_SS_06094_10836	Signal Transduction	others	.	Rop guanine nucleotide exchange factor 1 (RopGEF1) (Kinase partner protein-like) (KPP-like)	unknown [Zea mays]			down (-4.1000,-4.0600)	0	1	0	1
SCBGLR1118B02.g	GS1_SS_08110_16284	Signal Transduction	others	Two component	response regulator (ARR-like)	Response regulator 7		down (-3.1800,-3.3300)	down (-4.8300,-4.9100)	0	2	0	2
SCCCRZ2003H05.g	GS1_SS_01098_15058	Signal Transduction	protein-protein interaction	Transcription Factor	hormone-related/auxin/Aux/IAA	Auxin-responsive protein IAA9		down (-2.4500,-2.6400)		0	1	0	1
SCQSRT2031H06.g	GS1_SS_13683_17635	Signal Transduction	protein-protein interaction	Stress	Water stress	induced	down (-2.2400,-1.7400)	up (10.0000,5.2600)	up (5.3300,7.9300)	2	1	0	3
SCSGHR1067C04.g	GS1_SS_01814_00696	Signal Transduction	protein-protein interaction	Receptors	receptor Ser/Thr kinase	caneRLK-DV3 (LRR)		down (-3.4100,-5.3700)	down (-6.6600,-6.7500)	0	2	0	2
SCEPRZ3045E10.g	GS1_SS_20157_14564	Signal Transduction	quinase	Protein kinase	MAPK/MAPKK/MAPKKK	caneNRK-1			up (4.9900,3.5800)	1	0	0	1
SCBGLR1117G09.g	GS1_SS_18272_13256	Signal Transduction	quinase	Protein kinase	Putative RLCK	caneRLCK-AV2			down (-2.9200,-3.2300)	0	1	0	1
SCJLRT2049D02.g	GS1_AS_01296_18211	Signal Transduction	quinase	Protein kinase	other	caneTousled-1			up (6.2600,6.5100)	1	0	0	1
SCSGAM2075E08.g	GS1_AS_05087_21469	Signal Transduction	quinase	Protein kinase	calcium-related	caneCDPK-20		up (2.8900,3.6300)		1	0	0	1
SCRUFL3065A06.g	GS1_SS_22866_10088	Signal Transduction	quinase	.	Uncharacterized protein sll0005	Os02g0816600 [Oryza sativa (japonica cultivar-group)] Os02g0816600 [Oryza sativa (japonica cultivar-group)]			down (-2.8100,-4.4300)	0	1	0	1
SCEQRT2101A04.g	GS1_SS_07546_00754	Signal Transduction	receptor	.	Xenotropic and polytropic retrovirus receptor 1 homolog	unknown [Zea mays]	down (-1.8300,-1.8200)			0	1	0	1
SCCCCL3004D07.b	GS1_SS_05819_07196	Signal Transduction	receptor	.	Probable gibberellin receptor GID1L1; GID1-like protein 1	unknown [Zea mays]			down (-4.0700,-4.9700)	0	1	0	1
SCJFRT1060C03.g	GS1_SS_12307_02901	Signal Transduction	ribosomal protein	.	Putative ribonuclease H protein At1g65750	unknown [Zea mays]			up (3.5500,4.9000)	1	0	0	1
SCVPRZ3029B05.g	GS1_SS_33378_21782	Signal Transduction	targeting protein	Transcription Factor	Aux/IAA	unknown [Zea mays]		up (4.2800,4.1300)	up (3.2800,4.8800)	2	0	0	2
SCAGLR1064B08.g	GS1_SS_22576_17541	Signal Transduction	transcription factor	Transcription Factor	hormone-related/ethylene/AP2/EREBP	AP2 domain-containing protein RAP2.3			up (3.7100,6.5400)	1	0	0	1
SCQGAM1046F11.g	GS1_SS_02800_21499	Signal Transduction	transcription factor	Protein metabolism	Early auxin-induced protein 22	Auxin response factor 16 hypothetical protein OsJ_019622 [Oryza sativa (japonica cultivar-group)]			up (2.7300,3.0200)	1	0	0	1
SCEPRT2048E07.g	GS1_SS_06752_18913	Signal Transduction	transcription factor	Two component	response regulator (ARR-like)	Response regulator 6		down (-2.6600,-3.5800)		0	1	0	1

SCCCCL4002B07.g	GS1_SS_06324_18757	Signal Transduction	transcription factor	Transcription Factor	AP2/EREBP	EREBP			up (5.6200,6.4400)	1	0	0	1
SCVPLR2005H03.g	GS1_SS_02369_18630	Signal Transduction	transcription factor	Transcription Factor	hormone-related/auxin/Aux/IAA	Auxin-responsive protein IAA16		down (-3.4700,-3.6100)	down (-4.3400,-5.1400)	0	2	0	2
SCJLRZ1019A02.g	GS1_SS_14454_19561	Signal Transduction	transcription factor	Transcription Factor	MYB	unknown [Zea mays]			down (-3.8900,-6.1800)	0	1	0	1
SCJFRZ2015F02.g	GS1_SS_14894_19741	Signal Transduction	transcription factor	Transcription Factor	MYB	unknown [Zea mays]			down (-4.2800,-5.0000)	0	1	0	1
SCCCLR1C04F11.g	GS1_SS_16337_17285	Signal Transduction	transcription factor	Transcription Factor	WRKY	zinc finger transcription factor WRKY1			up (2.6500,3.2600)	1	0	0	1
SCCCCL3140E02.g	GS1_SS_08163_00789	Signal Transduction	transcription factor	.	NAC domain-containing protein 77; ONAC077; ONAC300	unknown [Zea mays]			up (3.8400,3.4400)	1	0	0	1
SCEZRT2019E05.g	GS1_SS_07121_18103	Signal Transduction	transcription factor	Transcription Factor	NAM (no apical meristem)	NAC domain-containing protein 77			up (3.5700,3.1500)	1	0	0	1
SCEQRT2093D08.g	GS1_SS_07042_18864	Signal Transduction	transcription factor	Transcription Factor	hormone-related/auxin/Aux/IAA	Auxin-responsive protein IAA12		down (-3.3100,-5.0500)	down (-6.5500,-7.1800)	0	2	0	2
SCCCLB1002G02.g	GS1_AS_00796_02724	Signal Transduction	transcription factor	.	Long-chain-fatty-acid--CoA ligase 1; Long-chain acyl-CoA synthetase 1; LACS 1; Palmitoyl-CoA ligase	hypothetical protein OsJ_004433 [Oryza sativa (japonica cultivar-group)]		up (1.9300,2.7400)		1	0	0	1
SCMCCL6049H11.g	GS1_SS_06667_04784	Signal Transduction	transcription factor	.	.	WRKY DNA-binding protein [Oryza sativa (indica cultivar-group)]		down (-2.3900,-3.6800)		0	1	0	1
SCEPAM2015F01.g	GS1_SS_04013_18978	Signal Transduction	transcription factor	Transcription Factor	hormone-related/auxin	B3 and AUX/IAA domains/Auxin response factor 1 (ARF1)			down (-2.6200,-4.4600)	0	1	0	1
SCEZHR1083B10.g	GS1_SS_25265_20642	Signal Transduction	transcription factor	Transcription Factor	HLH	unknown [Zea mays]			down (-3.8300,-4.4600)	0	1	0	1
SCSBHR1056H09.g	GS1_SS_19678_20080	Signal Transduction	transcription factor	Transcription Factor	MYB	unknown [Zea mays]		up (2.1200,3.4500)	down (-3.8000,-4.8500)	1	1	0	2
SCEZRT2015G07.g	GS1_SS_02549_10966	Signal Transduction	transcription factor	.	AP2-like ethylene-responsive transcription factor At2g41710	unknown [Zea mays]			down (-3.3700,-3.8200)	0	1	0	1
SCRURT2010A10.g	GS1_SS_19708_19813	Signal Transduction	transcription factor	Transcription Factor	MYB	Transcription factor (myb)		down (-3.5900,-4.1900)	down (-4.2000,-6.3000)	0	2	0	2
SCEPLR1008A10.g	GS1_SS_23522_14785	Signal Transduction	transcription factor	Transcription Factor	hormone-related/auxin/Aux/IAA	Auxin-responsive protein IAA31		down (-2.4200,-4.2300)	down (-6.0400,-6.4700)	0	2	0	2
SCEQRT1024G12.g	GS1_SS_00606_15578	Signal Transduction	transcription factor	Transcription Factor	MYB	hypothetical protein LOC100194332 [Zea mays] unknown [Zea mays]			down (-3.3500,-5.9000)	0	1	0	1
SCJFRZ2009F04.g	GS1_SS_18640_19368	Signal Transduction	transcription factor	Transcription Factor	hormone-related/auxin/Aux/IAA	Auxin-responsive protein IAA30			down (-3.1400,-4.5000)	0	1	0	1
SCAGAD1073C08.g	GS1_SS_19747_12612	Signal Transduction	transcription factor	.	.	hypothetical protein LOC100193483 [Zea mays] unknown [Zea mays]		down (-4.8600,-7.4400)	down (-6.9200,-8.3000)	0	2	0	2
SCSBAD1085B04.g	GS1_SS_19582_21271	Signal Transduction	transcription factor	Transcription Factor	MYB	hypothetical protein OsI_037518 [Oryza sativa (indica cultivar-group)]			down (-4.0000,-3.6200)	0	1	0	1
SCJLRZ1024E12.g	GS1_SS_21320_04282	Signal Transduction	transcription factor	Transcription Factor	GARP transfactor	Os06g0703900 [Oryza sativa (japonica cultivar-group)] putative transfactor [Oryza sativa Japonica Group] Os06g0703900 [Oryza sativa (japonica cultivar-group)]			down (-5.7400,-6.9100)	0	1	0	1
SCMCSB1116C07.g	GS1_SS_21714_03585	Signal Transduction	transcription factor	.	.	hypothetical protein OsJ_028987 [Oryza sativa (japonica cultivar-group)]		up (2.7800,2.6900)	up (3.8400,3.5100)	2	0	0	2
SCVPRZ3030F10.g	GS1_SS_16613_19521	Signal Transduction	transcription factor	Transcription Factor	Trihelix	Os04g0541100 [Oryza sativa (japonica cultivar-group)] Os04g0541100 [Oryza sativa (japonica cultivar-group)]		up (2.1200,2.8700)	up (4.9500,3.2000)	2	0	0	2
SCEQLR1050A01.g	GS1_SS_07747_16950	Signal Transduction	transcription factor	Nucleic acid metabolism	regulation of transcription, DNA-dependent	DEAD box protein		down (-4.0700,-6.6000)	down (-4.6500,-7.8500)	0	2	0	2
SCCCCL6003A11.g	GS1_SS_17744_16222	Signal Transduction	transcription factor	Transcription Factor	Aux/IAA	unknown [Zea mays]			down (-2.9400,-3.3700)	0	1	0	1
SCQGST1032D08.g	GS1_SS_17182_20815	Signal Transduction	transcription factor	Transcription Factor	MYB	unknown [Zea mays]			down (-4.5000,-4.8100)	0	1	0	1
SCMCRT2105A02.g	GS1_SS_16948_21060	Signal Transduction	transcription factor	Transcription Factor	MYB	hypothetical protein LOC100193196 [Zea mays] unknown [Zea mays] unknown [Zea mays]		down (-4.0100,-5.6000)	down (-4.4100,-6.8100)	0	2	0	2
SCEZLB1008F08.g	GS1_SS_20825_16504	Signal Transduction	transcription factor	Transcription Factor	hormone-related/auxin	B3 and AUX/IAA domains/Auxin response factor 6 (ARF6)			down (-2.7400,-4.0800)	0	1	0	1
SCEQLR1007F11.g	GS1_SS_11642_17166	Signal Transduction	transcription factor	Transcription Factor	Homeobox	unknown [Zea mays]			down (-4.6700,-3.7800)	0	1	0	1
SCEQRT2027F03.g	GS1_SS_24101_09842	Signal Transduction	transcription factor	Transcription Factor	Myb	myb family transcription factor-like [Oryza sativa Japonica Group]			down (-3.4500,-4.5800)	0	1	0	1
SCEPRZ3086F09.g	GS1_SS_17350_07825	Signal Transduction	Transcriptional Factor	.	JmjC domain-containing protein 5; Jumonji domain-containing protein 5	unknown [Zea mays]		down (-3.5900,-7.1000)		0	1	0	1
SCRFAM2128A12.g	GS1_SS_04922_18207	Signal Transduction	Transcriptional Factor	Transcription Factor	MADS	caneCKI-17			down (-4.5900,-5.2900)	0	1	0	1
SCSFHR1042G07.g	GS1_SS_08458_14656	Signal Transduction	Transcriptional Factor	Transcription Factor	WRKY	unknown [Zea mays]			up (3.2700,3.2500)	1	0	0	1
SCCCLR1079C08.g	GS1_SS_21356_20703	Signal Transduction	Transcriptional Factor	Transcription Factor	Others	HGM			down (-4.3600,-5.0800)	0	1	0	1

SCEQAM2039A10.g	GS1_SS_24094_21208	Signal Transduction	Transcriptional Factor	Transcription Factor	hormone-related/gibberellin	Gibberellin response modulator			down (-3.7800,-4.7300)	0	1	0	1
SCCCST3006E12.g	GS1_SS_18371_02905	Signal Transduction	Uncharacterized	.	Auxin-induced in root cultures protein 12;	hypothetical protein OsI_030866 [Oryza sativa (indica cultivar-group)]			down (-3.8100,-4.1000)	0	1	0	1
SCVPLB1017D05.g	GS1_SS_09530_20091	Signal Transduction	Uncharacterized	Stress	.	induced			up (5.9200,5.1400)	1	0	0	1
SCEZLB1008D09.g	GS1_SS_24236_20445	Signal Transduction	Uncharacterized	Transcription Factor	SBP (squamosa promoter binding protein)	SBP-domain protein 5			down (-3.9400,-6.1300)	0	1	0	1
SCCCCL5071H01.g	GS1_SS_03343_11640	Signal Transduction	Uncharacterized	.	Coleoptile phototropism protein 1 (Non-phototropic hypocotyl 3-like protein) (NPH3-like protein)	unknown [Zea mays]		up (2.4900,2.7400)		1	0	0	1
SCMCCL6053D03.g	GS1_SS_28758_18327	Signal Transduction	zinc-binding protein	Transcription Factor	Zinc finger proteins	C2C2		up (5.0000,3.8700)		1	0	0	1
SCEPLB1042B08.g	GS1_SS_00283_19610	Signal Transduction		Carbohydrate metabolism	Starch synthase	pseudo-response regulator 1 [Arabidopsis thaliana]		down (-3.8900,-3.6300)		0	1	0	1
SCAGLR1021F02.g	GS1_SS_20432_08284	Signal Transduction		.	SAC domain-containing protein 3; FIG4 homolog	hypothetical protein OsI_010031 [Oryza sativa (indica cultivar-group)]		up (2.7600,2.8300)		1	0	0	1
SCAGFL3024G12.g	GS1_SS_22570_11616	Sulphur metabolism	transporter	.	Probable sulfate transporter 3.3; AST91	hypothetical protein OsJ_021458 [Oryza sativa (japonica cultivar-group)]			down (-3.3300,-5.3100)	0	1	0	1
SCRURT2012H06.g	GS1_SS_13826_14124	Transporters	channel	.	Ras-related protein RABH1B	unknown [Zea mays]			up (4.8400,3.5200)	1	0	0	1
SCBGRT1050B11.g	GS1_SS_11936_13398	Transporters	hydrolase	.	Calcium-transporting ATPase 1, endoplasmic reticulum-type	hypothetical protein OsI_017571 [Oryza sativa (indica cultivar-group)]			up (3.1500,3.9300)	1	0	0	1
SCEQRT1024C07.g	GS1_SS_12011_14550	Transporters	kinase/phosphatase	.	Pyrophosphate-energized vacuolar membrane proton pump; Pyrophosphate-energized inorganic pyrophosphatase; H(+)-PPase	proton translocating pyrophosphatase [Oryza sativa]			up (4.1700,3.6500)	1	0	0	1
SCQSHR1024F04.g	GS1_SS_07499_06860	Transporters	Membrane transporters ABC transporter	.	Probable white-brown complex homolog protein 16	unknown [Zea mays]			up (5.2800,4.5200)	1	0	0	1
SCEZRT3070D05.g	GS1_SS_25332_01944	Transporters	Membrane transporters ABC transporter	.	Multidrug resistance protein 9 (P-glycoprotein 9)	hypothetical protein OsI_003330 [Oryza sativa (indica cultivar-group)]			up (5.4500,4.8900)	1	0	0	1
SCEQRT1027D08.g	GS1_SS_01904_02882	Transporters	Membrane transporters ABC transporter	.	Pleiotropic drug resistance protein 3; OsPDR9	Pleiotropic drug resistance protein 3; OsPDR9 hypothetical protein OsI_002763 [Oryza sativa (indica cultivar-group)]			up (3.3800,3.5200)	1	0	0	1
SCQGHR1014E03.g	GS1_SS_07879_15885	Transporters	Membrane transporters Nodulin-like protein	Stress	infected library	Nodulin (MtN21)		down (-5.7900,-6.2100)	down (-7.8700,-6.7500)	0	2	0	2
SCJFRZ2014D05.g	GS1_SS_01342_10250	Transporters	Membrane transporters Nodulin-like protein	.	Auxin-induced protein 5NG4	unknown [Zea mays]	down (-3.0300,-1.4100)	up (1.8900,3.4100)		1	1	0	2
SCJFRT1059C11.g	GS1_SS_12288_21519	Transporters	MIP	Stress	Others	Drought and cold response		up (6.0400,2.9200)		1	0	0	1
SCBFFL4113C07.g	GS1_SS_24221_00216	Transporters	others	.	Peptide transporter PTR2; Histidine-transporting protein	unknown [Zea mays]		down (-5.4900,-6.8300)	down (-5.7200,-6.1800)	0	2	0	2
SCCCLR1C04G11.g	GS1_AS_19109_01090	Transporters	translocator	.	.	unknown [Zea mays]		up (3.6500,3.5700)	up (3.5200,3.2800)	2	0	0	2
SCQGRT1042A05.g	GS1_SS_12681_15506	Transporters	translocator	Protein metabolism	Signal recognition particle 54 kDa protein, chloroplast precursor (SRP54) (54 chloroplast p	unknown [Zea mays]			down (-3.0600,-2.8400)	0	1	0	1
SCCCLR1C04G11.g	GS1_SS_19109_01089	Transporters	translocator	.	.	unknown [Zea mays]		up (5.9600,3.9000)	up (4.2800,5.3300)	2	0	0	2
SCJFAM1067F03.b	GS1_SS_25840_13009	Transporters	transporter	.	Boron transporter-like protein 2	unknown [Zea mays]			down (-4.2800,-6.2500)	0	1	0	1
SCJFAM1067F03.g	GS1_SS_02429_06002	Transporters	transporter	.	Boron transporter-like protein 2	unknown [Zea mays]			down (-4.3100,-4.9100)	0	1	0	1
SCMCCL6053C09.g	GS1_SS_06720_17873	Transporters	transporter	Cell cycle	peptide transport protein homolog	Os01g0871500 [Oryza sativa (japonica cultivar-group)] Os01g0871500 [Oryza sativa (japonica cultivar-group)]			down (-3.4000,-3.4000)	0	1	0	1
SCEZRZ3016G06.g	GS1_SS_18922_18909	Transporters	transporter	Transporters	Sugar transporters	putative sugar transporter [Oryza sativa]			down (-4.7600,-5.1800)	0	1	0	1
SCJLRZ3076A04.b	GS1_SS_15996_18583	Transporters	transporter	Protein metabolism	Arsenite transport protein (ARSB)	unknown [Zea mays]			down (-3.2600,-3.0800)	0	1	0	1
SCEPFL3081B06.g	GS1_SS_17846_12980	Transporters	transporter	.	.	unknown [Zea mays]			down (-5.5100,-5.4200)	0	1	0	1
SCJFHR1C03B08.g	GS1_SS_07825_01043	Transporters	transporter	.	Peptide transporter PTR2; Histidine-transporting protein	hypothetical protein OsI_036617 [Oryza sativa (indica cultivar-group)]		down (-3.8100,-5.0000)	down (-7.7200,-9.2200)	0	2	0	2
SCEQRT1030B05.g	GS1_SS_17430_19886	Transporters	transporter	Transporters	Transmembrane amino acid transporter protein (Aa_trans)	unknown [Zea mays]		up (2.1200,2.9600)		1	0	0	1
SCEZAM2031A08.g	GS1_SS_04157_17040	Transporters	transporter	No matches	.	.		down (-4.0200,-5.9400)	up (5.0800,6.0200)	1	1	0	2
SCAGLR2018G04.g	GS1_SS_09989_06827	Transporters	transporter	.	RING-H2 finger protein ATL1B	unknown [Zea mays]			down (-4.0700,-4.8100)	0	1	0	1

SCSGAD1008G12.g	GS1_SS_01414_11953	Transporters	transporter	.	Multidrug resistance-associated protein 4; Glutathione S-conjugate-transporting ATPase 4; ATP-energized glutathione S-conjugate pump 4	hypothetical protein OsI_014322 [Oryza sativa (indica cultivar-group)]	up (1.2300,1.5900)			1	0	0	1
SCEZLB1008D12.g	GS1_SS_25529_14609	Transporters	transporter	Transporters	Major intrinsic protein (MIP)	unknown [Zea mays]			up (4.7300,5.5800)	1	0	0	1
SCAGAD1077C06.g	GS1_SS_19784_10342	Transporters	transporter	.	.	hypothetical protein OsJ_031543 [Oryza sativa (japonica cultivar-group)]		up (2.2300,2.5800)		1	0	0	1
SCQSHR1022H06.g	GS1_SS_22805_08816	Transporters	transporter	.	Probable sulfate transporter 3.4	unknown [Zea mays]		up (2.1100,4.5500)	up (3.6000,4.6400)	2	0	0	2
SCEZHR1048H09.g	GS1_SS_07642_16466	Transporters	transporter	Stress	infected library	CLC-d chloride channel/anion channel protein	up (1.3700,1.7300)			1	0	0	1
SCCCSD2C03E07.g	GS1_SS_25499_08928	Transporters	Transporters	.	White-brown complex homolog protein 2	hypothetical protein OsI_017568 [Oryza sativa (indica cultivar-group)]			up (3.3200,3.3600)	1	0	0	1
SCJFRT1059B01.g	GS1_SS_14034_18210	Transporters	Transporters	Transporters	(xan_ur_permease)	permease 1 [Zea mays] Nucleobase-ascorbate transporter LPE1; Leaf permease protein 1 permease 1 [Zea mays]			up (5.6700,4.1800)	1	0	0	1
SCBGLR1047G11.g	GS1_SS_07822_20133	Transporters	Transporters	Transporters	Transmembrane amino acid transporter protein (Aa_trans)	unknown [Zea mays]			up (4.1800,3.9300)	1	0	0	1
SCCCCL6005D07.g	GS1_SS_06508_05364	Transporters	Transporters	.	Multidrug resistance-associated protein 5; Glutathione S-conjugate-transporting ATPase 5; ATP-energized glutathione S-conjugate pump 5	low phytic acid 1 [Zea mays] low phytic acid 1 [Zea mays]		up (2.8400,2.7800)		1	0	0	1
SCAGLR2026C05.g	GS1_SS_08080_12307	Transporters	Transporters	.	.	unknown [Zea mays]		down (-2.7600,-3.5400)	down (-3.6200,-3.8800)	0	2	0	2
SCMCRT2104G02.g	GS1_SS_07178_19847	Transporters	Transporters	Transporters	Transmembrane amino acid transporter protein (Aa_trans)	unknown [Zea mays]			up (2.9700,3.9900)	1	0	0	1
SCSGFL4C08B10.g	GS1_SS_22942_15410	Transporters	Transporters	Transporters	POT family (PTR2)	hypothetical protein LOC100191709 [Zea mays] unknown [Zea mays]			down (-7.1700,-4.2400)	0	1	0	1
SCRLAM1008F10.g	GS1_SS_03051_10950	Transporters	Transporters	.	Oligopeptide transporter 7; AtOPT7	Os06g0125400 [Oryza sativa (japonica cultivar-group)] putative sexual differentiation process protein isp4 [Oryza sativa Japonica Group] Os06g0125400 [Oryza sativa (japonica cultivar-group)] hypothetical protein OsI_020708 [Oryza sativa (indica culti			up (3.5700,3.6100)	1	0	0	1
SCACLR1126C04.g	GS1_SS_20075_02432	Transporters		.	Protein TRANSPARENT TESTA 12	hypothetical protein LOC100191901 [Zea mays] unknown [Zea mays]		up (4.5000,2.9200)		1	0	0	1
SCJFRZ2005F08.g	GS1_SS_01259_09895	Transporters		.	Peptide transporter PTR2; Histidine-transporting protein	unknown [Zea mays]			down (-4.0300,-5.5700)	0	1	0	1
SCCCCL3001B06.g	GS1_SS_05409_16585	Transporters		Transporters	Transmembrane amino acid transporter protein (Aa_trans)	unknown [Zea mays]	up (1.8800,1.1900)			1	0	0	1
SCEPRZ1009H11.g	GS1_SS_16533_19194	Transporters		Transporters	Transmembrane amino acid transporter protein (Aa_trans)	Os05g0424000 [Oryza sativa (japonica cultivar-group)] putative amino acid transporter [Oryza sativa (japonica cultivar-group)] Os05g0424000 [Oryza sativa (japonica cultivar-group)] hypothetical protein OsI_019340 [Oryza sativa (indica cultivar-group)			down (-5.1500,-4.0700)	0	1	0	1
SCEPSD2073G05.g	GS1_SS_25947_09753	Transposable element related	nucleic acid binding	.	.	putative MURAZC [Zea mays]			up (3.1500,3.3700)	1	0	0	1
SCJLST1020F05.g	GS1_SS_07880_06470	Unknown	ATP synthase	.	.	hypothetical protein LOC100194276 [Zea mays] unknown [Zea mays]		down (-4.3000,-6.8000)		0	1	0	1
SCRFHR1006H10.g	GS1_AS_07908_17435	Unknown	bacteria	No matches	.	.		down (-3.8900,-5.3900)		0	1	0	1
SCJFRZ1006B05.g	GS1_SS_07772_00349	Unknown	dehydrogenase	.	Probable aldehyde dehydrogenase; Flax-inducible sequence 1	putative aldehyde dehydrogenase MIS1 [Zea mays]			up (3.7300,3.2900)	1	0	0	1

SCSGST1068B03.g	GS1_SS_01714_12885	Unknown	GTPase	.	Spo0B-associated GTP-binding protein	hypothetical protein OsJ_024509 [Oryza sativa (japonica cultivar-group)]			down (-3.2900,-3.7300)	0	1	0	1
SCMCLR1053E06.g	GS1_AS_21705_18834	Unknown	hydrolase	lipid metabolism	MLO-like protein 4; AtMlo4	Putative lysophospholipase		up (4.2500,5.1300)		1	0	0	1
SCCCSD2001B07.g	GS1_SS_25461_00978	Unknown	inhibitor	.	Subtilisin-chymotrypsin inhibitor-2A (CI-2A)	MPI [Zea diploperennis] MPI [Zea perennis] MPI [Zea perennis]			up (8.2700,7.0300)	1	0	0	1
SCJFRT1061G09.g	GS1_SS_12346_13960	Unknown	kinase/phosphatase	.	Nucleotide pyrophosphatase/phosphodiesterase	hypothetical protein LOC100191759 [Zea mays] unknown [Zea mays]		down (-5.3900,-6.4000)	down (-3.4900,-4.1400)	0	2	0	2
SCJLST1025F01.g	GS1_SS_03716_02296	Unknown	kinase/phosphatase	.	.	.		down (-2.9800,-6.0200)		0	1	0	1
SCVPRZ3029B10.g	GS1_SS_33379_17011	Unknown	kinase/phosphatase	Receptors	Receptor Ser/Thr kinase	caneWAK-14 (wall-associated kinase)			down (-5.7400,-5.9000)	0	1	0	1
SCUTSB1031H05.g	GS1_SS_17391_14928	Unknown	kinase/phosphatase	Protein kinase	Cell cycle-related	caneCDC2-7		down (-3.1500,-3.1600)		0	1	0	1
SCVPFL3047F02.b	GS1_SS_22369_08794	Unknown	kinase/phosphatase	.	Bifunctional purple acid phosphatase 26; Includes: Acid phosphatase; Includes: Peroxidase;	unknown [Zea mays]			up (3.9900,3.4200)	1	0	0	1
SCEQSD2078G04.g	GS1_SS_42314_18381	Unknown	kinase/phosphatase	Pathogenicity	R-genes	caneXa21-20		up (2.6300,3.0400)		1	0	0	1
SCCCST1005H10.g	GS1_SS_08877_21083	Unknown	methyltransferase	Stress	drought and cold response	early-responsive to dehydration stress protein (erd3-like)			up (3.6800,4.0400)	1	0	0	1
SCEQRT1031D05.g	GS1_SS_17169_12484	Unknown	methyltransferase	.	Phosphoethanolamine N-methyltransferase 1; PEAMT 1; AtNMT1	unknown [Zea mays]		up (2.5400,2.9100)		1	0	0	1
SCEPLB1041E10.g	GS1_SS_05313_18090	Unknown	methyltransferase	Stress	drought and cold response	early-responsive to dehydration stress protein (erd3-like)			down (-4.7800,-6.5700)	0	1	0	1
SCRLAM1008E06.g	GS1_SS_19310_08151	Unknown	motor protein	.	Ribosome-binding protein 1; Ribosome receptor protein; 180 kDa ribosome receptor homolog; ES/130-related protein	hypothetical protein OsI_008955 [Oryza sativa (indica cultivar-group)]			down (-3.2700,-5.4200)	0	1	0	1
SCCCAM2C06A08.g	GS1_SS_05619_13903	Unknown	no matches	.	.	.		up (4.8400,3.0100)	up (3.7300,4.8500)	2	0	0	2
SCBGLR1002B09.g	GS1_SS_16279_00786	Unknown	no matches	.	.	.			down (-4.4500,-3.7500)	0	1	0	1
SCAGFL3022F05.g	GS1_SS_22556_11250	Unknown	no matches	.	.	.		up (2.5900,2.6200)	up (5.1600,5.5200)	2	0	0	2
SCACSD2018G06.g	GS1_SS_25493_01997	Unknown	no matches	No matches	.	.			up (3.8700,7.0400)	1	0	0	1
SCACLR1126E09.g	GS1_SS_09869_21044	Unknown	no matches	No matches	.	.			down (-4.6400,-4.9600)	0	1	0	1
SCCCLB1004B04.g	GS1_AS_08853_01039	Unknown	no matches	.	.	.		up (2.0500,3.7700)		1	0	0	1
SCCCLR1075G06.g	GS1_SS_08894_12329	Unknown	no matches	.	.	.			down (-4.2400,-5.4200)	0	1	0	1
SCBGFL5082D09.g	GS1_AS_23288_07140	Unknown	no matches	.	.	.		down (-2.2200,-4.0900)		0	1	0	1
SCBGLR1097F08.g	GS1_SS_09904_02249	Unknown	no matches	.	.	.		up (4.2700,3.0500)		1	0	0	1
SCACRZ3036E03.g	GS1_SS_15261_16985	Unknown	no matches	No matches	.	.			down (-4.3800,-5.4200)	0	1	0	1
SCCCLR2C01C08.g	GS1_SS_11280_04357	Unknown	no matches	.	.	.	up (1.3200,1.5100)			1	0	0	1
SCCCCL4007D09.g	GS1_SS_06055_10447	Unknown	no matches	.	.	.		up (2.3200,4.3000)	up (6.4100,6.5400)	2	0	0	2
SCBGLR1115D10.g	GS1_SS_18145_17897	Unknown	no matches	No matches	non-coding	.		up (3.7100,3.4900)		1	0	0	1
SCCCLR2C02F09.g	GS1_SS_11309_07707	Unknown	no matches	.	.	.		down (-2.4700,-3.3100)	down (-3.4100,-6.5900)	0	2	0	2
SCBFRT1064H06.g	GS1_SS_11768_01958	Unknown	no matches	.	.	.		down (-2.1400,-3.2500)		0	1	0	1
SCACSD2014F07.g	GS1_SS_26292_06600	Unknown	no matches	.	.	.			up (3.5700,3.5000)	1	0	0	1
SCACRZ3036E03.g	GS1_AS_15261_16984	Unknown	no matches	No matches	.	.			down (-3.6600,-4.3200)	0	1	0	1
SCBFLB2095D11.g	GS1_SS_25852_13258	Unknown	no matches	.	.	.		down (-2.2400,-5.2300)	down (-3.0700,-3.4400)	0	2	0	2
SCACLR1126G08.g	GS1_SS_09620_01141	Unknown	no matches	.	.	.		down (-2.9000,-2.7300)	down (-3.2000,-4.2300)	0	2	0	2
SCCCLR1C11G04.g	GS1_SS_10833_13109	Unknown	no matches	.	.	.			up (3.1100,4.9200)	1	0	0	1
SCCCLR1072H12.g	GS1_AS_11447_00378	Unknown	no matches	.	.	.		up (2.9000,2.9500)		1	0	0	1
SCACSB1036E06.g	GS1_SS_03359_12105	Unknown	no matches	.	.	.			up (3.0800,4.3000)	1	0	0	1
SCCCCL4017D01.g	GS1_SS_06414_05448	Unknown	others	.	.	hypothetical protein SORBIDRAFT_05g023750 [Sorghum bicolor] hypothetical protein SORBIDRAFT_05g023750 [Sorghum bicolor]			up (2.9100,4.2400)	1	0	0	1
SCCCLR2004G12.g	GS1_SS_18856_00961	Unknown	others	.	.	unknown [Zea mays]		up (5.1200,3.2900)		1	0	0	1
SCBGAD1026G02.g	GS1_SS_08893_06172	Unknown	others	.	.	Lipase, putative, expressed [Oryza sativa (japonica cultivar-group)] hypothetical protein OsI_037622 [Oryza sativa (indica cultivar-group)]		down (-3.3200,-4.4800)	down (-6.0100,-6.4100)	0	2	0	2
SCBGST3107A01.g	GS1_SS_21249_09170	Unknown	others	.	.	unknown [Zea mays]			down (-3.6700,-5.6600)	0	1	0	1
SCCCRZ2003H01.g	GS1_SS_03682_02989	Unknown	others	.	.	unknown [Zea mays]		down (-6.7000,-5.4300)	up (3.9000,3.3900)	1	1	0	2
SCACLB1046B10.g	GS1_SS_08871_08840	Unknown	others	.	.	hypothetical protein SORBIDRAFT_03g045310 [Sorghum bicolor] hypothetical protein SORBIDRAFT_03g045310 [Sorghum bicolor]		down (-2.1900,-2.3200)	down (-2.6800,-4.5100)	0	2	0	2
SCCCRZ2C04F06.g	GS1_SS_15870_02299	Unknown	others	.	.	unknown [Zea mays]			down (-5.9900,-5.3900)	0	1	0	1

SCCCLR1078G03.g	GS1_SS_10546_12685	Unknown	others	.	.	Os06g0502800 [Oryza sativa (japonica cultivar-group)] Os06g0502800 [Oryza sativa (japonica cultivar-group)]		up (6.6100,4.4600)	up (4.4100,5.9500)	2	0	0	2
SCBGLR1096E09.g	GS1_SS_15900_08023	Unknown	others	.	.	unknown [Zea mays]			down (-3.8200,-3.8200)	0	1	0	1
SCAGAM2122A09.g	GS1_SS_17829_12391	Unknown	others	.	.	hypothetical protein OsJ_021686 [Oryza sativa (japonica cultivar-group)]			down (-4.4000,-4.1500)	0	1	0	1
SCBFLR1083G03.g	GS1_SS_01635_09392	Unknown	others	.	.	hypothetical protein LOC100191296 [Zea mays] unknown [Zea mays]			down (-3.7100,-4.1900)	0	1	0	1
SCBFLR1005H01.g	GS1_SS_16661_12921	Unknown	others	.	.	unknown [Zea mays]			up (5.1400,6.1100)	1	0	0	1
SCCCLR1022B02.g	GS1_SS_02510_05883	Unknown	others	.	.	unknown [Zea mays]		down (-3.0200,-4.1800)	down (-3.6100,-7.0600)	0	2	0	2
SCBFSB1045A05.g	GS1_SS_16713_12056	Unknown	others	.	.	hypothetical protein OsJ_012928 [Oryza sativa (japonica cultivar-group)]			down (-1.7600,-4.8000)	0	1	0	1
SCCCRZ3003E03.g	GS1_SS_15381_08260	Unknown	others	.	.	unknown [Zea mays]		down (-2.2100,-2.8400)		0	1	0	1
SCCCAM1001G06.g	GS1_SS_01599_02839	Unknown	others	.	.	unknown [Zea mays] unknown [Zea mays]			down (-3.7600,-4.2700)	0	1	0	1
SCCCLR1C02C06.g	GS1_SS_01595_19615	Unknown	others	Nucleic acid metabolism	.	DnaJ homologue (At5g06910)			down (-4.7100,-5.0400)	0	1	0	1
SCCCRZ2C03B10.g	GS1_SS_10041_10553	Unknown	others	.	.	unknown [Zea mays]			down (-5.5900,-4.1000)	0	1	0	1
SCBGLR1114D06.g	GS1_SS_00137_14312	Unknown	others	.	.	unknown [Zea mays]			down (-4.1500,-2.8700)	0	1	0	1
SCCCFL1001H12.g	GS1_SS_01299_08633	Unknown	others	.	.	unknown [Zea mays]		up (2.7200,3.7000)	up (4.8700,5.4300)	2	0	0	2
SCCCFL8C03H05.g	GS1_SS_26254_07396	Unknown	others	.	.	hypothetical protein OsJ_018438 [Oryza sativa (japonica cultivar-group)]		down (-2.9100,-3.4300)		0	1	0	1
SCAGLR1021G12.g	GS1_SS_19297_07941	Unknown	others	.	.	unknown [Zea mays]			down (-5.5200,-4.4900)	0	1	0	1
SCCCRT1002B11.g	GS1_SS_11712_18967	Unknown	others	Carbohydrate metabolism	(Q40007) Putative 32.7 kDa jasmonate-induced protein	hypothetical protein OsI_035081 [Oryza sativa (indica cultivar-group)]		down (-2.7200,-4.2100)	down (-3.8700,-4.1100)	0	2	0	2
SCCCLR1072G11.g	GS1_SS_10129_08956	Unknown	others	.	.	unknown [Zea mays]			down (-5.1100,-5.9900)	0	1	0	1
SCACLR1126B12.g	GS1_SS_09614_09565	Unknown	others	.	.	.		down (-3.6300,-5.1100)	down (-6.0500,-6.6300)	0	2	0	2
SCCCLR1065C08.g	GS1_SS_10150_13292	Unknown	others	.	.	unknown [Zea mays]		up (3.4900,4.4000)		1	0	0	1
SCACLR1129A10.g	GS1_SS_09637_01035	Unknown	others	.	.	unknown [Zea mays]			down (-4.5800,-4.5800)	0	1	0	1
SCAGLR1064F09.g	GS1_SS_09716_00596	Unknown	others	.	.	.		down (-3.1000,-3.9700)		0	1	0	1
SCAGLR2018E01.g	GS1_SS_11377_00526	Unknown	others	.	.	unknown [Zea mays]			up (3.5400,4.9600)	1	0	0	1
SCBGLR1003C06.g	GS1_SS_10718_11560	Unknown	others	.	.	unknown [Zea mays]			up (4.5200,5.5900)	1	0	0	1
SCCCLB1004E03.g	GS1_SS_20718_10339	Unknown	others	.	2,3-cyclic-nucleotide 2-phosphodiesterase	unknown [Zea mays]		down (-2.8400,-3.9200)		0	1	0	1
SCCCCL2001H12.b	GS1_SS_05715_13689	Unknown	others	.	.	hypothetical protein OsJ_010070 [Oryza sativa (japonica cultivar-group)]			up (4.1700,7.0300)	1	0	0	1
SCBGLR1082G01.g	GS1_SS_13186_14071	Unknown	others	.	.	unknown [Zea mays]		down (-2.9900,-7.3600)	down (-5.6400,-8.5000)	0	2	0	2
SCBGFL5079D09.g	GS1_SS_13196_07823	Unknown	others	.	.	unknown [Zea mays]		up (1.8300,2.7200)		1	0	0	1
SCACST3160H08.g	GS1_SS_01439_02319	Unknown	others	.	.	.		down (-3.7300,-5.5700)		0	1	0	1
SCAGAM2125E03.g	GS1_SS_03760_10041	Unknown	others	.	.	hypothetical protein OsI_024392 [Oryza sativa (indica cultivar-group)]			down (-5.2200,-5.4600)	0	1	0	1
SCCCAM1073A05.g	GS1_SS_01858_07474	Unknown	others	.	.	unknown [Zea mays]			down (-3.6000,-4.5700)	0	1	0	1
SCCCST2004E05.g	GS1_SS_17839_02986	Unknown	oxidoreductase	.	Probable flavin-containing monooxygenase 1	unknown [Zea mays]		down (-2.3600,-4.2300)	down (-4.8500,-4.9300)	0	2	0	2
SCVPLB1018G04.g	GS1_SS_09546_13291	Unknown	oxidoreductase	.	Electron transfer flavoprotein-ubiquinone oxidoreductase, mitochondrial; ETF-ubiquinone oxidoreductase; ETF-QO; Electron-transferring-flavoprotein dehydrogenase; ETF dehydrogenase;	Electron transfer flavoprotein-ubiquinone oxidoreductase,mitochondrial precursor, putative, expressed [Oryza sativa (japonica cultivar-group)]		up (1.9500,2.5700)	up (3.6900,3.7200)	2	0	0	2
SCQSLR1018A06.g	GS1_SS_10920_08940	Unknown	protease	.	Mitochondrial inner membrane protease ATP23	unknown [Zea mays]		down (-2.3700,-3.1700)		0	1	0	1
SCEPLB1043F06.g	GS1_SS_18547_10976	Unknown	protease	.	Aspartic proteinase nepenthesin-2; Nepenthesin-II;	hypothetical protein OsJ_018716 [Oryza sativa (japonica cultivar-group)]		up (6.7300,3.4600)	up (3.8300,6.9100)	2	0	0	2
SCMCAM1100B05.g	GS1_SS_20511_10943	Unknown	protein-protein interaction	.	Nodulation receptor kinase;	Os01g0140400 [Oryza sativa (japonica cultivar-group)] senescence-induced receptor-like serine/threonine kinase -like [Oryza sativa Japonica Group] Os01g0140400 [Oryza sativa (japonica cultivar-group)]			down (-3.7100,-4.4200)	0	1	0	1

SCVPRT2081D11.g	GS1_SS_18735_01249	Unknown	Reductase	.	Flavoprotein wrbA (Trp repressor-binding protein)	Os05g0501300 [Oryza sativa (japonica cultivar-group)] putative 1,4-benzoquinone reductase [Oryza sativa (japonica cultivar-group)] Os05g0501300 [Oryza sativa (japonica cultivar-group)] hypothetical protein OsI_019826 [Oryza sativa (indica cultivar-	down (-1.4400,-2.6200)			0	1	0	1
SCJLRZ1021F11.g	GS1_SS_02406_12748	Unknown	ribosomal protein	.	50S ribosomal protein L18, chloroplastic; CL18;	unknown [Zea mays]		down (-4.0200,-4.6900)	down (-5.4800,-5.5300)	0	2	0	2
SCEZHR1049C07.g	GS1_SS_07647_21820	Unknown	Ribosomal protein	Stress	Water stress	repressed		down (-2.4300,-3.1200)	down (-4.4100,-4.8100)	0	2	0	2
SCRLRZ3039A08.g	GS1_SS_15820_19419	Unknown	RNA-binding protein	Stress	Water stress	induced		down (-3.0100,-2.6400)		0	1	0	1
SCCCST1002H06.g	GS1_SS_15854_18993	Unknown	synthase	Secondary metabolism	flavonol synthase	unknown [Zea mays]	down (-1.2300,-2.0000)			0	1	0	1
SCEPRT2047F02.g	GS1_SS_12953_15288	Unknown	synthase	Lipid, fatty-acid and isoprenoid metabolism	Phytoene synthase 2, chloroplast precursor (EC 2.5.1.-)	phytoene synthase 2 [Zea mays]			down (-5.1100,-5.7300)	0	1	0	1
SCJLRZ1027E04.g	GS1_SS_12905_02854	Unknown	Transferase family	.	Sulfotransferase 18; AtSOT18; Desulfoglucosinolate sulfotransferase B; AtST5b	unknown [Zea mays]		down (-6.2400,-5.9700)	down (-5.4800,-6.3300)	0	2	0	2
SCJFRZ3C04A07.g	GS1_AS_15809_15643	Unknown	transporter	Protein metabolism	GDP-mannose transporter	GONST2 Golgi Nucleotide sugar transporter, putative [Oryza sativa (japonica cultivar-group)] hypothetical protein OsJ_012615 [Oryza sativa (japonica cultivar-group)]			down (-4.2000,-5.2500)	0	1	0	1
SCCCST2002A02.g	GS1_SS_18309_11412	Unknown	transporter	.	Uncharacterized membrane protein At1g63010	SPX (SYG1/Pho81/XPR1) domain-containing protein-like [Oryza sativa Japonica Group] SPX (SYG1/Pho81/XPR1) domain-containing protein-like [Oryza sativa Japonica Group]	down (-1.4400,-2.8300)			0	1	0	1
SCSFAM1076F04.g	GS1_SS_03238_02452	Unknown	transporter	.	Probable potassium transporter 16; OsHAK16	hypothetical protein OsJ_011049 [Oryza sativa (japonica cultivar-group)]			down (-3.3000,-4.3500)	0	1	0	1
SCJLRT1016A01.g	GS1_SS_12474_06120	Unknown	ubiquitination	.	Autophagy-related protein 7; Autophagy-related E1-like-activating enzyme ATG7; ATG12-activating E2-like enzyme; Protein autophagy APG7; AtAPG7	hypothetical protein OsI_002807 [Oryza sativa (indica cultivar-group)]			up (3.9000,4.0200)	1	0	0	1
SCEZAD1C01D10.g	GS1_SS_00508_12015	Unknown	Uncharacterized	.	.	.			up (4.1600,6.9400)	1	0	0	1
SCEPAM1019B10.g	GS1_SS_01941_08126	Unknown	uncharacterized	.	DnaJ homolog subfamily C member 2; Zuotin-related factor 1; Mouse Id associate 1; MIDA1	hypothetical protein OsI_008455 [Oryza sativa (indica cultivar-group)]	down (-3.3000,-5.3200)			0	1	0	1
SCQGAM2028B12.g	GS1_SS_16315_13266	Unknown	uncharacterized	.	.	Os01g0194000 [Oryza sativa (japonica cultivar-group)] Os01g0194000 [Oryza sativa (japonica cultivar-group)]		down (-2.0100,-4.0600)		0	1	0	1
SCSFAD1069H10.g	GS1_SS_01211_13210	Unknown	uncharacterized	.	.	unknown [Zea mays]		down (-3.7200,-2.7700)	down (-4.7400,-4.3400)	0	2	0	2
SCSFSB1105C04.g	GS1_SS_26164_00448	Unknown	Uncharacterized	.	.	unknown [Zea mays] unknown [Zea mays]		up (3.0300,2.8300)		1	0	0	1
SCQSRT1034D09.g	GS1_SS_12729_12754	Unknown	Uncharacterized	.	Sodium-coupled neutral amino acid transporter 1; Amino acid transporter A1; System A amino acid transporter 1; System N amino acid transporter 1; N-system amino acid transporter 2; Solute carrier family 38 member 1	Os02g0191300 [Oryza sativa (japonica cultivar-group)] putative amino acid transporter A1 [Oryza sativa Japonica Group] putative amino acid transporter A1 [Oryza sativa Japonica Group] Os02g0191300 [Oryza sativa (japonica cultivar-group)] hypothetical			up (5.5100,5.6100)	1	0	0	1
SCEPAM1020B08.g	GS1_SS_01956_02298	Unknown	uncharacterized	.	Probable 125 kDa kinesin-related protein	kinesin heavy chain [Zea mays]		down (-3.0000,-4.2300)		0	1	0	1
SCJFRZ2027A05.g	GS1_SS_11557_08583	Unknown	Uncharacterized	.	.	unknown [Zea mays]		down (-3.3100,-3.5100)	down (-4.3900,-5.7500)	0	2	0	2
SCUTLR2023A03.g	GS1_SS_04374_14495	Unknown	Uncharacterized	.	Esterase precursor (Early nodule-specific protein homolog) (Latex allergen Hev b 13)	unknown [Zea mays]		down (-3.2700,-4.2200)		0	1	0	1
SCVPRZ3029F03.g	GS1_SS_00314_13853	Unknown	Uncharacterized	.	.	unknown [Zea mays]		down (-6.7200,-6.8700)	down (-6.8600,-8.2300)	0	2	0	2
SCRLRZ3114C04.g	GS1_SS_16282_12967	Unknown	uncharacterized	.	.	unknown [Zea mays]	down (-1.1000,-1.7700)			0	1	0	1

SCJLLR1033A12.g	GS1_AS_01627_05729	Unknown	Uncharacterized	.	Transmembrane protein 147	Os03g0766000 [Oryza sativa (japonica cultivar-group)] Os03g0766000 [Oryza sativa (japonica cultivar-group)]			down (-4.5400,-4.6200)	0	1	0	1
SCMCAM2082H05.g	GS1_SS_02418_07359	Unknown	Uncharacterized	.	.	unknown [Zea mays]			up (4.5400,3.6600)	1	0	0	1
SCSBSD2029B08.g	GS1_SS_25484_04225	Unknown	uncharacterized	.	.	.		up (3.3400,3.1800)		1	0	0	1
SCCCST3005G09.g	GS1_SS_18360_05918	Unknown	Uncharacterized	.	.	unknown [Zea mays]		down (-2.7500,-4.2000)		0	1	0	1
SCVPRT2073H08.g	GS1_SS_14056_12468	Unknown	Uncharacterized	.	.	Os10g0559400 [Oryza sativa (japonica cultivar-group)] retrotransposon protein, putative, unclassified, expressed [Oryza sativa (japonica cultivar-group)] Os10g0559400 [Oryza sativa (japonica cultivar-group)]		up (2.9200,5.4300)		1	0	0	1
SCVPRZ3030G09.g	GS1_SS_22543_20618	Unknown	uncharacterized	Stress	Water stress	induced		up (4.6800,3.8300)	up (5.7100,6.7700)	2	0	0	2
SCEQAD1018E12.g	GS1_SS_00479_12977	Unknown	uncharacterized	.	.	.			up (2.9400,3.3000)	1	0	0	1
SCSBSD2030A01.g	GS1_SS_25489_01178	Unknown	Uncharacterized	.	Glutelin type-B 5 precursor [Glutelin type-B 5 acidic chain; Glutelin type-B 5 basic chain]	legumin 1 [Zea mays] unknown [Zea mays]		down (-3.1900,-3.0700)	down (-2.2200,-3.9300)	0	2	0	2
SCCCLR1070H01.g	GS1_SS_10230_06550	Unknown	uncharacterized	.	.	unknown [Zea mays]		down (-3.0400,-3.5400)		0	1	0	1
SCEZLB1005B03.g	GS1_SS_02909_02112	Unknown	Uncharacterized	.	Nucleolar complex protein 2 homolog; Protein NOC2 homolog	Os12g0163200 [Oryza sativa (japonica cultivar-group)] Uncharacterised protein family containing protein, expressed [Oryza sativa (japonica cultivar-group)] Os12g0163200 [Oryza sativa (japonica cultivar-group)]		down (-2.0300,-3.5200)		0	1	0	1
SCVPLR2012A07.g	GS1_SS_12036_12107	Unknown	Uncharacterized	.	.	Os04g0564700 [Oryza sativa (japonica cultivar-group)] Os04g0564700 [Oryza sativa (japonica cultivar-group)]		up (2.0500,3.8800)		1	0	0	1
SCJFRZ2025E01.g	GS1_AS_16600_21831	Unknown	uncharacterized	Unknown protein	.	.			up (4.8200,5.2800)	1	0	0	1
SCUTST3087E03.g	GS1_SS_18963_10075	Unknown	uncharacterized	.	.	hypothetical protein OsI_018089 [Oryza sativa (indica cultivar-group)]		up (2.5500,4.8300)		1	0	0	1
SCSBSD1058E10.g	GS1_SS_41919_16256	Unknown	uncharacterized	Receptors	Receptor Ser/Thr kinase-unclassified	caneURLK-120 (with LRR)		down (-2.8800,-3.6500)		0	1	0	1
SCRFST1041H05.g	GS1_SS_05103_03622	Unknown	uncharacterized	.	.	unknown [Zea mays]			down (-3.1500,-3.0600)	0	1	0	1
SCBFST3133G05.g	GS1_SS_18453_03878	Unknown	uncharacterized	.	.	unknown [Zea mays]			down (-3.2900,-6.7200)	0	1	0	1
SCRLLR1038F07.g	GS1_SS_12299_12089	Unknown	uncharacterized	.	.	unknown [Zea mays]	down (-1.1700,-1.2800)			0	1	0	1
SCQGFL4079D05.g	GS1_SS_14500_13207	Unknown	Uncharacterized	.	.	Os01g0120500 [Oryza sativa (japonica cultivar-group)] unknown protein [Oryza sativa Japonica Group] unknown protein [Oryza sativa Japonica Group] Os01g0120500 [Oryza sativa (japonica cultivar-group)]			down (-2.9400,-2.8800)	0	1	0	1
SCAGRT2039D11.g	GS1_SS_12834_13883	Unknown	Uncharacterized	.	.	hypothetical protein SORBIDRAFT_10g006790 [Sorghum bicolor] hypothetical protein SORBIDRAFT_10g006790 [Sorghum bicolor]		up (2.8300,2.9000)		1	0	0	1
SCJFRZ2006C07.g	GS1_SS_05098_11319	Unknown	uncharacterized	.	.	unknown [Zea mays]		down (-2.4400,-7.2100)		0	1	0	1
SCMCRZ3065E11.g	GS1_SS_00834_11349	Unknown	Uncharacterized	.	.	hypothetical protein OsJ_028750 [Oryza sativa (japonica cultivar-group)]		down (-4.0400,-4.1200)		0	1	0	1
SCBFRZ2048B09.g	GS1_SS_18430_09463	Unknown	Uncharacterized	.	DUF246 domain-containing protein At1g04910	unknown [Zea mays]			down (-3.4400,-3.7200)	0	1	0	1
SCEQLB1067F01.g	GS1_SS_12045_19144	Unknown	uncharacterized	Carbohydrate metabolism	vegetative storage protein	provA¡vel relaA§A£o com ferimentos e assimilaÃ§Ã£o de nitrogÃªnio		down (-3.6900,-7.0800)		0	1	0	1
SCJLRZ1020H11.g	GS1_SS_14486_01492	Unknown	uncharacterized	.	.	hypothetical protein OsJ_030943 [Oryza sativa (japonica cultivar-group)]		down (-2.6300,-4.4900)		0	1	0	1

SCJFRT2060A01.g	GS1_SS_13426_13153	Unknown	Uncharacterized	.	.	Os06g0235200 [Oryza sativa (japonica cultivar-group)] leaf senescence protein-like [Oryza sativa Japonica Group] leaf senescence protein-like [Oryza sativa Japonica Group] Os06g0235200 [Oryza sativa (japonica cultivar-group)] hypothetical protein OsI		down (-3.7000,-5.0800)	down (-5.0000,-5.6600)	0	2	0	2
SCEPAM1018G02.g	GS1_SS_18912_05375	Unknown	uncharacterized	.	.	hypothetical protein OsJ_010041 [Oryza sativa (japonica cultivar-group)]		down (-4.1200,-6.1500)	down (-4.1700,-6.2200)	0	2	0	2
SCMCRT2085F06.g	GS1_SS_08580_00148	Unknown	uncharacterized	.	.	unknown [Zea mays]			down (-5.0100,-4.3600)	0	1	0	1
SCJLLR1101D08.g	GS1_SS_10629_00361	Unknown	uncharacterized	.	.	unknown [Zea mays]			down (-3.1900,-5.9900)	0	1	0	1
SCACAD1039H11.g	GS1_SS_00187_09901	Unknown	Uncharacterized	.	Uncharacterized protein At1g14870	unknown [Zea mays]			down (-3.8700,-4.4700)	0	1	0	1
SCRFLB1053C11.g	GS1_SS_18012_14443	Unknown	Uncharacterized	.	SWI/SNF complex subunit SWI3D; AtSWI3D; Transcription regulatory protein SWI3D	unknown [Zea mays]		down (-2.5300,-4.7600)		0	1	0	1
SCMCRT2087B07.g	GS1_SS_13555_17889	Unknown	Uncharacterized	Receptors	receptor Ser/Thr kinase	caneRLK-DVI4 (LRR)		down (-2.0900,-3.7300)		0	1	0	1
SCMCRT2087A05.g	GS1_SS_13553_01236	Unknown	Uncharacterized	.	.	unknown [Zea mays]			up (4.3800,4.2800)	1	0	0	1
SCSGHR1070F11.g	GS1_SS_08598_06625	Unknown	Uncharacterized	.	.	unknown [Zea mays]		down (-3.1800,-3.2300)		0	1	0	1
SCSGLR1045B04.g	GS1_SS_09893_02559	Unknown	Uncharacterized	.	.	unknown [Zea mays]		down (-4.2300,-5.8400)	down (-7.1200,-6.8100)	0	2	0	2
SCBGLR1118F01.g	GS1_SS_09897_12713	Unknown	Uncharacterized	.	.	Os10g0503600 [Oryza sativa (japonica cultivar-group)] putative senescence-associated protein [Oryza sativa (japonica cultivar-group)] senescence-associated family protein, putative, expressed [Oryza sativa (japonica cultivar-group)] Os10g0503600 [Ory			down (-2.9500,-3.1400)	0	1	0	1
SCVPLR1049E07.g	GS1_SS_11214_00800	Unknown	uncharacterized	.	.	hypothetical protein OsJ_011924 [Oryza sativa (japonica cultivar-group)]		up (2.8500,2.9700)		1	0	0	1
SCEPAM2056G08.g	GS1_SS_16429_09681	Unknown	Uncharacterized	.	Uncharacterized transporter YBR287W	unknown [Zea mays]		up (4.9400,2.6800)		1	0	0	1
SCBSBD1056F06.g	GS1_SS_25729_14516	Unknown	uncharacterized	.	.	.			down (-3.4300,-3.6000)	0	1	0	1
SCUTLR1037C05.g	GS1_SS_11172_07917	Unknown	uncharacterized	.	Nectarin-1; Superoxide dismutase [Mn];	germin-like protein 6a [Hordeum vulgare subsp. vulgare]		down (-4.5800,-6.0300)		0	1	0	1
SCEZRZ1017F11.g	GS1_SS_09859_02722	Unknown	Uncharacterized	.	.	unknown [Zea mays]		down (-1.9700,-3.0100)		0	1	0	1
SCUTLR1037G04.g	GS1_SS_11178_12825	Unknown	uncharacterized	.	.			up (3.2200,4.5700)	up (3.2600,4.2000)	2	0	0	2
SCEQSB1C02G10.g	GS1_SS_19403_14533	Unknown	uncharacterized	.	Uncharacterized membrane protein At1g63010	hypothetical protein LOC100193704 [Zea mays] unknown [Zea mays]		up (3.2500,3.3900)		1	0	0	1
SCCCLR1C09A04.g	GS1_SS_09943_11731	Unknown	Uncharacterized	.	.	.		down (-2.5700,-3.3700)		0	1	0	1
SCACLB1045B09.g	GS1_SS_08860_12706	Unknown	Uncharacterized	.	F-box/WD repeat-containing protein pof10; Skp1-binding protein 2	unknown [Zea mays]		up (2.3300,2.6000)		1	0	0	1
SCJFRZ2013G01.g	GS1_SS_14876_01578	Unknown	uncharacterized	.	.	unknown [Zea mays]	down (-3.4100,-2.8900)			0	1	0	1
SCBGFL4056D06.g	GS1_SS_22045_14342	Unknown	Uncharacterized	.	Probable E3 ubiquitin-protein ligase HERC1; HECT domain and RCC1-like domain-containing protein 1; p532; p619	unknown [Zea mays]			down (-3.5400,-5.0500)	0	1	0	1
SCSGLV1006E08.g	GS1_SS_26109_08722	Unknown	Uncharacterized	.	.	Os01g0138400 [Oryza sativa (japonica cultivar-group)] putative receptor serine/threonine kinase PR5K [Oryza sativa Japonica Group] Os01g0138400 [Oryza sativa (japonica cultivar-group)] stress-induced receptor-like kinase 2 [Oryza sativa (indica culti			up (7.4200,3.9000)	1	0	0	1
SCEQLR1050B01.g	GS1_SS_10359_08785	Unknown	uncharacterized	.	.	unknown [Zea mays]			up (3.0000,3.4700)	1	0	0	1
SCSBAD1053B05.g	GS1_SS_01146_09019	Unknown	Uncharacterized	Cell cycle	Protein bimA	unknown [Zea mays]			up (2.8700,3.5100)	1	0	0	1
SCJLST1024H09.g	GS1_SS_17586_12600	Unknown	Uncharacterized	.	Cysteine-rich repeat secretory protein 12;	hypothetical protein OsI_021616 [Oryza sativa (indica cultivar-group)]		down (-4.1300,-5.2900)		0	1	0	1
SCJFST1048C08.g	GS1_SS_16005_09886	Unknown	uncharacterized	.	.	unknown [Zea mays]			down (-5.8200,-5.2200)	0	1	0	1
SCJFRT2060A10.g	GS1_SS_12427_17238	Unknown	Uncharacterized	No matches	.	unknown [Zea mays]			up (5.5600,6.0500)	1	0	0	1
SCVPRT2078D10.g	GS1_SS_14106_09883	Unknown	uncharacterized	.	.	.			up (3.5800,5.5200)	1	0	0	1

SCACLB1046G06.g	GS1_SS_08878_11602	Unknown	Uncharacterized	.	.	hypothetical protein OsI_024565 [Oryza sativa (indica cultivar-group)] hypothetical protein OsJ_022720 [Oryza sativa (japonica cultivar-group)]			up (2.7000,3.4800)	1	0	0	1
SCJLRZ3076G11.g	GS1_SS_15994_12790	Unknown	Uncharacterized	.	.	hypothetical protein OsJ_032609 [Oryza sativa (japonica cultivar-group)]			up (2.8600,3.8800)	1	0	0	1
SCEPLR1008G07.g	GS1_SS_10294_13665	Unknown	Uncharacterized	.	.	.		up (3.8600,3.0100)	up (3.9300,4.1500)	2	0	0	2
SCQSFL1121B01.g	GS1_AS_24042_14863	Unknown	Uncharacterized	No matches	.	.		up (2.5600,3.4200)		1	0	0	1
SCSGLV1008B03.g	GS1_SS_26120_13402	Unknown	uncharacterized	.	.	unknown [Zea mays]			up (5.4600,5.2200)	1	0	0	1
SCRLLR1059C02.g	GS1_SS_14164_13949	Unknown	Uncharacterized	.	.	unknown [Zea mays]		up (4.7400,5.0200)	up (7.2700,6.7900)	2	0	0	2
SCCCCL4007A12.g	GS1_SS_19395_03758	Unknown	Uncharacterized	.	.	hypothetical protein OsI_009337 [Oryza sativa (indica cultivar-group)]		up (2.7600,3.1200)		1	0	0	1
SCJLLR1107C08.g	GS1_SS_13249_09692	Unknown	Uncharacterized	.	Probable carbohydrate esterase At4g34215	unknown [Zea mays]		down (-2.5900,-3.8500)		0	1	0	1
SCCCLR1048A03.g	GS1_SS_15979_14228	Unknown	Uncharacterized	.	IgA FC receptor; Beta antigen; B antigen;	unknown [Zea mays]			up (7.4000,5.1900)	1	0	0	1
SCUTLR2008H10.g	GS1_AS_09610_06204	Unknown	uncharacterized	.	.	unknown [Zea mays]			up (3.4400,4.5100)	1	0	0	1
SCEQLB1068G06.g	GS1_SS_19385_08576	Unknown	Uncharacterized	.	Uncharacterized GPI-anchored protein At3g06035;	unknown [Zea mays]		down (-3.6900,-3.7500)		0	1	0	1
SCSBFL1039G06.g	GS1_AS_20092_00178	Unknown	uncharacterized	.	.	hypothetical protein [Oryza sativa Japonica Group] hypothetical protein [Oryza sativa Japonica Group]		up (2.2500,3.3500)		1	0	0	1
SCSGFL4C04A05.g	GS1_SS_21899_01687	Unknown	Uncharacterized	.	.	hypothetical protein OsJ_016534 [Oryza sativa (japonica cultivar-group)]			down (-3.9300,-3.0400)	0	1	0	1
SCQSLR1089H10.g	GS1_SS_15896_10232	Unknown	uncharacterized	.	.	unknown [Zea mays]	down (-2.8000,-1.8800)			0	1	0	1
SCVPCL6044A06.g	GS1_SS_07071_11868	Unknown	Uncharacterized	.	.	.			up (3.4900,3.5500)	1	0	0	1
SCJFLR1035F07.g	GS1_SS_07081_09349	Unknown	Uncharacterized	.	Something about silencing protein 10; Disrupter of silencing SAS10; UTP3 homolog; Charged amino acid-rich leucine zipper 1; CRL1	Os01g0104800 [Oryza sativa (japonica cultivar-group)] unknown protein [Oryza sativa Japonica Group] unknown protein [Oryza sativa Japonica Group] Os01g0104800 [Oryza sativa (japonica cultivar-group)]		down (-3.0400,-3.6000)		0	1	0	1
SCEQSB1C03A07.g	GS1_SS_17381_14275	Unknown	Uncharacterized	.	.	unknown [Zea mays]			up (2.6600,4.9700)	1	0	0	1
SCQSAM2100G02.g	GS1_SS_08780_09807	Unknown	uncharacterized	.	.	unknown [Zea mays]			up (5.8100,6.2700)	1	0	0	1
SCVPCL6045C01.g	GS1_SS_07084_00155	Unknown	uncharacterized	.	.	putative retroelement protein [Sorghum bicolor]			up (5.2600,5.3700)	1	0	0	1
SCRFAM1026C04.g	GS1_SS_17369_05686	Unknown	Uncharacterized	.	.	unknown [Zea mays]			up (2.8800,3.8800)	1	0	0	1
SCSBAM1088A01.g	GS1_SS_03195_08289	Unknown	uncharacterized	.	.	Os09g0469900 [Oryza sativa (japonica cultivar-group)] putative queuine tRNA-ribosyltransferase [Oryza sativa Japonica Group] putative queuine tRNA-ribosyltransferase [Oryza sativa Japonica Group] Os09g0469900 [Oryza sativa (japonica cultivar-group)]		down (-2.8700,-3.0900)		0	1	0	1
SCVPRZ2037E03.g	GS1_AS_15121_03483	Unknown	Uncharacterized	.	.	.			down (-4.0400,-4.1500)	0	1	0	1
SCMCST1052G03.g	GS1_SS_17900_05313	Unknown	Uncharacterized	.	.	hypothetical protein LOC100192523 [Zea mays] unknown [Zea mays]		down (-2.2600,-3.1600)		0	1	0	1
SCSGFL4031B05.g	GS1_SS_21854_14453	Unknown	uncharacterized	.	.	hypothetical protein OsI_019840 [Oryza sativa (indica cultivar-group)]			down (-4.2900,-4.8100)	0	1	0	1
SCCCRZ3004E12.g	GS1_SS_15226_09240	Unknown	Uncharacterized	.	PsbP-like protein 1, chloroplastic; PsbP-related thylakoid lumenal protein 2; OEC23-like protein 4;	unknown [Zea mays]			down (-3.7100,-4.8800)	0	1	0	1
SCVPAM2065F07.g	GS1_SS_05589_14055	Unknown	Uncharacterized	.	.	.			up (3.3300,4.1400)	1	0	0	1
SCRUFL1120F01.g	GS1_SS_24040_13068	Unknown	Uncharacterized	.	.	unknown [Zea mays]		down (-5.2200,-4.0400)		0	1	0	1
SCCCCL3080D06.g	GS1_SS_10949_11586	Unknown	Uncharacterized	.	RING finger protein 126-B	unknown [Zea mays]			down (-2.6200,-3.1300)	0	1	0	1
SCSBAD1054F04.g	GS1_SS_20522_07380	Unknown	Uncharacterized	.	RCC1 and BTB domain-containing protein 1; Regulator of chromosome condensation and BTB domain-containing protein 1	unknown [Zea mays]			down (-3.0800,-4.9200)	0	1	0	1
SCQSRT2033E04.g	GS1_SS_13697_06821	Unknown	Uncharacterized	.	.	hypothetical protein LOC100191169 [Zea mays] unknown [Zea mays]		down (-2.1200,-5.0300)		0	1	0	1

SCRFLR2038A05.g	GS1_SS_04156_10152	Unknown	uncharacterized	.	Protein EXECUTER 1, chloroplastic;	unknown [Zea mays]		up (2.1700,2.9800)		1	0	0	1
SCEPAM1021H11.g	GS1_SS_01988_14366	Unknown	Uncharacterized	.	Rubredoxin; Rd	Os08g0323400 [Oryza sativa (japonica cultivar-group)] putative rubredoxin [Oryza sativa Japonica Group] Os08g0323400 [Oryza sativa (japonica cultivar-group)] hypothetical protein OsI_027854 [Oryza sativa (indica cultivar-group)]			down (-4.5600,-5.1100)	0	1	0	1
SCEPAM1022B03.g	GS1_SS_01990_03904	Unknown	Uncharacterized	.	.	unknown [Zea mays]		down (-3.1600,-4.9600)		0	1	0	1
SCVPCL6042H01.g	GS1_SS_02010_10791	Unknown	uncharacterized	.	.	unknown [Zea mays]			down (-3.3400,-3.2800)	0	1	0	1
SCEPAM1023G09.g	GS1_SS_02008_10627	Unknown	uncharacterized	.	.	unknown [Zea mays]		up (5.0400,6.2200)		1	0	0	1
SCRFFL1025D09.g	GS1_SS_23973_10738	Unknown	Uncharacterized	.	.	Os06g0603600 [Oryza sativa (japonica cultivar-group)] putative ids-4 protein [Oryza sativa Japonica Group] Os06g0603600 [Oryza sativa (japonica cultivar-group)] hypothetical protein OsJ_021053 [Oryza sativa (japonica cultivar-group)]	down (-1.5700,-1.5900)			0	1	0	1
SCRFST1043C02.g	GS1_SS_17125_05715	Unknown	uncharacterized	.	Ubiquinone biosynthesis methyltransferase coq-5, mitochondrial;	unknown [Zea mays]			down (-3.7500,-4.3100)	0	1	0	1
SCQSFL3037F08.b	GS1_SS_22639_11081	Unknown	uncharacterized	.	.	unknown [Zea mays]		down (-3.3300,-3.0900)		0	1	0	1
SCUTRZ3106F01.g	GS1_SS_17141_18080	Unknown	uncharacterized	No matches	non-coding	.			down (-2.7900,-5.3900)	0	1	0	1
SCRURT3064H08.g	GS1_SS_25020_13193	Unknown	Uncharacterized	.	Probable plastid-lipid-associated protein 8, chloroplast precursor (Fibrillin-8)	unknown [Zea mays]			down (-3.1800,-3.5600)	0	1	0	1
SCSGSB1006G07.g	GS1_SS_22670_12122	Unknown	uncharacterized	.	.	unknown [Zea mays]		down (-3.4900,-2.8800)		0	1	0	1
SCSBFL5018D05.g	GS1_SS_23264_08545	Unknown	uncharacterized	.	.	hypothetical protein LOC100192013 [Zea mays] unknown [Zea mays]			down (-3.9400,-5.1000)	0	1	0	1
SCUTFL3075D09.g	GS1_SS_23241_16748	Unknown	Uncharacterized	Transcription Factor	hormone-related/auxin/Aux/IAA	Auxin-responsive protein IAA25			down (-4.8500,-3.7600)	0	1	0	1
SCSBFL1044C08.g	GS1_SS_00345_11894	Unknown	uncharacterized	.	.	unknown [Zea mays]		down (-3.6100,-8.5400)	down (-3.9000,-6.6800)	0	2	0	2
SCUTFL1055C05.g	GS1_AS_21344_07587	Unknown	uncharacterized	.	.	.			up (3.8900,4.5000)	1	0	0	1
SCJLFL4183G10.g	GS1_SS_24287_11029	Unknown	uncharacterized	.	.	hypothetical protein OsI_014192 [Oryza sativa (indica cultivar-group)]			down (-6.8500,-8.7600)	0	1	0	1
SCRLAM1012C06.g	GS1_SS_04481_06854	Unknown	Uncharacterized	.	Rop guanine nucleotide exchange factor 2 (RopGEF2)	unknown [Zea mays]			down (-3.4500,-4.9400)	0	1	0	1
SCACAM1071F11.g	GS1_SS_01677_14995	Unknown	Uncharacterized	Receptors	receptor Ser/Thr kinase	caneS receptor-21		up (2.2500,2.5600)		1	0	0	1
SCVPST1061E07.g	GS1_SS_18283_16067	Unknown	Uncharacterized	Protein metabolism	Lysyl-tRNA synthetase (EC 6.1.1.6) (Lysine-tRNA ligase) (LysRS)	hypothetical protein OsJ_007346 [Oryza sativa (japonica cultivar-group)]		down (-2.5600,-2.4700)		0	1	0	1
SCEQRZ3091A05.g	GS1_SS_23603_09830	Unknown	Uncharacterized	.	.	unknown [Zea mays]			up (5.5500,3.8200)	1	0	0	1
SCEZHR1088F08.g	GS1_SS_07766_08810	Unknown	Uncharacterized	.	.	unknown [Zea mays]			down (-4.2000,-3.9500)	0	1	0	1
SCJFRZ1007H05.g	GS1_SS_17513_09918	Unknown	Uncharacterized	.	.	Os09g0456100 [Oryza sativa (japonica cultivar-group)] remorin protein-like [Oryza sativa Japonica Group] remorin protein-like [Oryza sativa Japonica Group] Os09g0456100 [Oryza sativa (japonica cultivar-group)]		down (-3.1000,-4.1000)		0	1	0	1
SCACAM1071C11.g	GS1_SS_01690_10145	Unknown	uncharacterized	.	Filament-like plant protein 1; AtFPP1	hypothetical protein OsI_034060 [Oryza sativa (indica cultivar-group)]		down (-2.6900,-5.0700)		0	1	0	1
SCJLRZ1024B01.g	GS1_SS_22617_13999	Unknown	uncharacterized	.	.	unknown [Zea mays]		down (-3.0000,-3.2400)		0	1	0	1
SCVPLR2019H02.g	GS1_SS_21581_04497	Unknown	Uncharacterized	.	Pyrophosphate-energized vacuolar membrane proton pump; Pyrophosphate-energized inorganic pyrophosphatase; H(+)-PPase; Vacuolar H(+)-pyrophosphatase	vacuolar H+-translocating inorganic pyrophosphatase [Zea mays]			down (-3.4400,-3.2700)	0	1	0	1
SCQGSD1045G04.g	GS1_SS_25462_11885	Unknown	Uncharacterized	.	Lysine histidine transporter-like 1 OS=Arabidopsis thaliana GN=At1g48640 PE=3 SV=2	unknown [Zea mays]			up (6.8800,5.7800)	1	0	0	1
SCEQLB1063H05.g	GS1_SS_09095_14043	Unknown	Uncharacterized	.	.	unknown [Zea mays]			down (-4.0500,-5.7200)	0	1	0	1
SCJFRZ2012G04.g	GS1_SS_00453_00482	Unknown	uncharacterized	.	Vacuolar transporter chaperone 2; Phosphate metabolism protein 1	unknown [Zea mays]	down (-1.9200,-1.7800)			0	1	0	1

SCSGAM2076F12.g	GS1_SS_05443_01262	Unknown	Uncharacterized	.	High affinity cationic amino acid transporter 1; CAT-1; CAT1; System Y+ basic amino acid transporter; Solute carrier family 7 member 1; Ecotropic retroviral leukemia receptor; Ecotropic retrovirus receptor; ERR	unknown [Zea mays]		up (3.1800,3.4500)	up (4.9400,4.4400)	2	0	0	2
SCJLST1019A04.g	GS1_SS_25442_07597	Unknown	uncharacterized	.	.	.			down (-3.7300,-3.0000)	0	1	0	1
SCSGAD1143A04.g	GS1_SS_23926_14280	Unknown	uncharacterized	.	.	Os03g0628800 [Oryza sativa (japonica cultivar-group)] H1flk, putative, expressed [Oryza sativa (japonica cultivar-group)] Os03g0628800 [Oryza sativa (japonica cultivar-group)]			down (-3.5800,-4.0000)	0	1	0	1
SCRFAM2127C07.g	GS1_SS_22733_00407	Unknown	uncharacterized	.	UPF0016 membrane protein sll0615	unnamed protein product [Vitis vinifera]			down (-4.6600,-6.9000)	0	1	0	1
SCMCCL6027C07.g	GS1_SS_06692_14115	Unknown	uncharacterized	.	.	unknown [Zea mays]			up (4.8700,5.3100)	1	0	0	1
SCJFRT2055A07.g	GS1_AS_13349_04391	Unknown	Uncharacterized	.	.	hypothetical protein OsI_019431 [Oryza sativa (indica cultivar-group)]		down (-2.3000,-3.6000)		0	1	0	1
SCSFAM1077G08.g	GS1_SS_03255_07840	Unknown	Uncharacterized	.	.	hypothetical protein SORBIDRAFT_01g013080 [Sorghum bicolor] hypothetical protein SORBIDRAFT_01g013080 [Sorghum bicolor]			up (4.2900,4.1700)	1	0	0	1
SCEPAM2053E04.g	GS1_SS_04031_10312	Unknown	Uncharacterized	.	.	unknown [Zea mays]			down (-3.1500,-3.8100)	0	1	0	1
SCEZFL5086G02.g	GS1_SS_23680_05168	Unknown	uncharacterized	.	.	unknown [Zea mays]		down (-3.5900,-4.2400)	down (-5.9800,-5.4400)	0	2	0	2
SCCCSB1003D09.g	GS1_SS_21552_04813	Unknown	uncharacterized	.	Protein IQ-DOMAIN 14	hypothetical protein OsI_004661 [Oryza sativa (indica cultivar-group)]		down (-3.0700,-4.1200)	down (-2.9300,-5.5900)	0	2	0	2
SCSFAM1077C08.g	GS1_SS_03248_12705	Unknown	Uncharacterized	.	.	.			up (3.4000,4.7600)	1	0	0	1
SCEZLB1014G11.g	GS1_SS_09548_09048	Unknown	Uncharacterized	.	Pollen-specific protein C13 precursor	unknown [Zea mays]		down (-2.2700,-7.0500)	down (-5.5100,-6.4600)	0	2	0	2
SCRULB1060H04.g	GS1_SS_02491_04922	Unknown	Uncharacterized	.	.	unknown [Zea mays]		up (4.1300,4.0400)		1	0	0	1
SCMCAM2080E02.g	GS1_SS_02481_16398	Unknown	Uncharacterized	Development	Transcriptional co-repressor LEUNIG	putative transcriptional corepressor LEUNIG [Oryza sativa Japonica Group]		up (4.0300,6.2000)	up (4.4300,4.6700)	2	0	0	2
SCCCHR1004C05.g	GS1_SS_07375_12099	Unknown	uncharacterized	No matches	.	Os05g0128100 [Oryza sativa (japonica cultivar-group)] Os05g0128100 [Oryza sativa (japonica cultivar-group)]		down (-3.4000,-4.9200)	down (-4.8400,-3.5000)	0	2	0	2
SCBFRZ2019D02.g	GS1_SS_03259_08864	Unknown	uncharacterized	No matches	non-coding	unknown [Zea mays]			down (-4.5600,-6.5100)	0	1	0	1
SCUTST3089H07.g	GS1_AS_16325_07363	Unknown	uncharacterized	.	.	hypothetical protein OsI_009930 [Oryza sativa (indica cultivar-group)]		up (2.5900,4.3900)	up (5.4900,4.4200)	2	0	0	2
SCRUAD1134G12.g	GS1_SS_25641_13345	Unknown	Uncharacterized	.	.	.			up (3.2400,3.5000)	1	0	0	1
SCEQRT2027E11.g	GS1_SS_12997_06596	Unknown	uncharacterized	.	.	hypothetical protein [Oryza sativa Japonica Group] hypothetical protein [Oryza sativa Japonica Group] hypothetical protein OsJ_001613 [Oryza sativa (japonica cultivar-group)]			down (-2.9100,-4.0900)	0	1	0	1
SCCCCL3001D12.g	GS1_SS_18681_12592	Unknown	uncharacterized	.	.	.			down (-4.8800,-3.6600)	0	1	0	1
SCJFRZ2031G06.g	GS1_SS_18112_09552	Unknown	uncharacterized	.	Putative leucine-rich repeat-containing protein DDB0188916	Os10g0504600 [Oryza sativa (japonica cultivar-group)] Chorion family 2 protein, expressed [Oryza sativa (japonica cultivar-group)] Os10g0504600 [Oryza sativa (japonica cultivar-group)]		down (-2.5600,-5.0300)	down (-3.4800,-5.6100)	0	2	0	2
SCRFST1045E11.g	GS1_SS_18115_12912	Unknown	Uncharacterized	.	.	Os04g0644600 [Oryza sativa (japonica cultivar-group)] OSJNBa0033G05.17 [Oryza sativa (japonica cultivar-group)] Os04g0644600 [Oryza sativa (japonica cultivar-group)] hypothetical protein OsJ_015670 [Oryza sativa (japonica cultivar-group)]			down (-3.6100,-3.6700)	0	1	0	1
SCEQRT1025F12.g	GS1_SS_18129_08434	Unknown	Uncharacterized	.	.	unknown [Zea mays]			down (-3.2000,-4.1500)	0	1	0	1
SCSGFL4036B09.g	GS1_SS_22987_07206	Unknown	Uncharacterized	.	.	unknown [Zea mays]			up (3.4700,3.3200)	1	0	0	1
SCSGAD1007F02.g	GS1_SS_01384_10946	Unknown	uncharacterized	.	.	unknown [Zea mays]		up (4.9700,5.2900)	up (4.1600,3.4400)	2	0	0	2
SCRLSD1013A03.g	GS1_SS_25276_09274	Unknown	Uncharacterized	No matches	.	.		up (3.1100,3.3200)		1	0	0	1

SCSGST3121C08.g	GS1_SS_19337_12324	Unknown	Uncharacterized	.	.	unknown [Zea mays]			down (-4.1100,-5.3600)	0	1	0	1
SCSGHR1070G09.g	GS1_AS_08601_04577	Unknown	uncharacterized	.	.	.			down (-2.7000,-4.4700)	0	1	0	1
SCEPLR1030C10.g	GS1_SS_12574_08648	Unknown	uncharacterized	.	Fasciclin-like arabinogalactan protein 16;	unknown [Zea mays]		down (-2.8600,-5.4200)	down (-3.2800,-6.0700)	0	2	0	2
SCSGST1069F09.g	GS1_SS_18187_00760	Unknown	Uncharacterized	.	Probable gibberellin receptor GID1L3; GID1-like protein 3	unknown [Zea mays]			down (-3.9200,-5.4100)	0	1	0	1
SCEPRZ1009A03.g	GS1_SS_14268_12617	Unknown	uncharacterized	.	.	unknown [Zea mays]			down (-6.1400,-4.1300)	0	1	0	1
SCSFAM1075A09.g	GS1_SS_24278_11465	Unknown	Uncharacterized	.	Photosystem II stability/assembly factor HCF136, chloroplastic;	plastid high chlorophyll fluorescence 136 precursor [Zea mays] unknown [Zea mays]			down (-3.5100,-4.6200)	0	1	0	1
SCUTSD1024A05.g	GS1_SS_25963_19440	Unknown	Uncharacterized	Carbohydrate metabolism	Isoamylase-type starch debranching enzyme ISO2	unknown [Zea mays]			down (-3.3000,-3.7300)	0	1	0	1
SCQGSD1048C09.g	GS1_SS_25961_16238	Unknown	uncharacterized	No matches	.	.		down (-4.0800,-3.9000)	down (-4.2800,-3.9000)	0	2	0	2
SCBGST3104A06.g	GS1_SS_22365_04682	Unknown	Uncharacterized	.	Pentatricopeptide repeat-containing protein At1g06580	hypothetical protein OsJ_017109 [Oryza sativa (japonica cultivar-group)]			down (-3.0600,-2.6400)	0	1	0	1
SCMCLR1053D05.g	GS1_SS_02233_07202	Unknown	Uncharacterized	.	.	Os02g0153000 [Oryza sativa (japonica cultivar-group)] ternary complex factor MIP1-like [Oryza sativa Japonica Group] ternary complex factor MIP1-like [Oryza rufipogon] ternary complex factor MIP1-like [Oryza sativa (indica cultivar-group)] Os02g01530		down (-4.1700,-3.5200)		0	1	0	1
SCVPFL3047B03.g	GS1_SS_22356_16195	Unknown	Uncharacterized	Development	cell differentiation	Annexin A13 (Annexin XIII) (Annexin, intestine-specific) (ISA)		down (-2.3500,-2.4900)		0	1	0	1
SCJLRT1022D02.g	GS1_SS_12603_01519	Unknown	Uncharacterized	.	.	hypothetical protein LOC100193259 [Zea mays] unknown [Zea mays]			down (-4.5100,-3.5800)	0	1	0	1
SCCCLR2C02E04.g	GS1_SS_11306_09232	Unknown	uncharacterized	.	Probable arylformamidase; Kynurenine formamidase; KF	unknown [Zea mays]		up (5.7100,3.3100)		1	0	0	1
SCEPLB1044H12.g	GS1_SS_25310_18827	Unknown	Uncharacterized	Unknown protein	.	unknown [Zea mays]			down (-3.4100,-3.4600)	0	1	0	1
SCRFSD1022C11.g	GS1_SS_25632_12776	Unknown	uncharacterized	.	.	branched-chain amino acid aminotransferase [Hordeum vulgare subsp. vulgare]		up (6.9400,5.1800)	up (6.7100,5.0300)	2	0	0	2
SCRFSD1022A08.g	GS1_SS_25631_19996	Unknown	Uncharacterized	Stress	.	induced			up (3.0500,3.2800)	1	0	0	1
SCMCLV1033A03.g	GS1_SS_26273_06159	Unknown	Uncharacterized	.	.	Os08g0515800 [Oryza sativa (japonica cultivar-group)] unknown protein [Oryza sativa Japonica Group] Os08g0515800 [Oryza sativa (japonica cultivar-group)] hypothetical protein OsJ_026814 [Oryza sativa (japonica cultivar-group)]		down (-2.7100,-3.9100)		0	1	0	1
SCUTFL1063H04.g	GS1_SS_22782_10003	Unknown	Uncharacterized	.	.	unknown [Zea mays]		down (-1.9800,-3.1900)		0	1	0	1
SCJLLR1107G06.g	GS1_SS_22777_13393	Unknown	uncharacterized	Carbohydrate metabolism	Aspartyl protease-like	hypothetical protein LOC100192571 [Zea mays] unknown [Zea mays] unknown [Zea mays]		down (-2.8200,-5.6300)		0	1	0	1
SCBFRZ2048F01.g	GS1_SS_26284_05683	Unknown	uncharacterized	.	Ser/Thr-rich protein T10 in DGCR region	unknown [Zea mays]			up (6.6100,6.4500)	1	0	0	1
SCVPRT2080A07.g	GS1_SS_01321_01595	Unknown	Uncharacterized	.	Low affinity sulfate transporter 3	Putative sulfate transporter [Oryza sativa (japonica cultivar-group)] hypothetical protein OsJ_009404 [Oryza sativa (japonica cultivar-group)]			down (-2.8300,-3.5400)	0	1	0	1
SCJLRT1023H11.g	GS1_SS_24859_18199	Unknown	Uncharacterized	No matches	.	unknown [Zea mays]			up (5.4500,5.8400)	1	0	0	1
SCQGFL3054F10.g	GS1_SS_23006_08233	Unknown	Uncharacterized	.	.	unknown [Zea mays]			down (-3.9200,-4.1600)	0	1	0	1
SCRFAM1025F12.g	GS1_SS_02662_13730	Unknown	uncharacterized	.	.	unknown [Zea mays]		down (-4.7600,-4.2400)	down (-5.2200,-6.9700)	0	2	0	2
SCEPSB1136B08.g	GS1_SS_18252_02954	Unknown	uncharacterized	.	.	unknown [Zea mays]			down (-3.0200,-3.8900)	0	1	0	1
SCCCLR1067B06.g	GS1_SS_18246_11096	Unknown	uncharacterized	.	Probable biogenesis of lysosome-related organelles complex 1 subunit 2; BLOC subunit 2	unknown [Zea mays]		up (1.8500,3.4200)	up (3.4700,4.3500)	2	0	0	2
SCEQRT2091D12.g	GS1_SS_13048_08838	Unknown	uncharacterized	.	Barwin	unknown [Zea mays]	up (1.4200,1.6700)			1	0	0	1
SCQGLB1038E11.g	GS1_SS_01280_11438	Unknown	Uncharacterized	.	.	unknown [Zea mays]		up (2.5000,3.6700)	up (3.9100,5.4400)	2	0	0	2
SCJFST1014A02.b	GS1_SS_12258_09006	Unknown	uncharacterized	.	.	.			up (3.4500,3.4100)	1	0	0	1
SCUTRZ3103B07.g	GS1_SS_24883_09341	Unknown	Uncharacterized	.	Uncharacterized 38.1 kDa protein	Os11g0109500 [Oryza sativa (japonica cultivar-group)] Os11g0109500 [Oryza sativa (japonica cultivar-group)]			down (-3.5800,-3.9800)	0	1	0	1

SCRLLR1110H11.g	GS1_SS_01284_12339	Unknown	uncharacterized	.	.	hypothetical protein LOC100191278 [Zea mays] unknown [Zea mays]			up (3.4300,3.9100)	1	0	0	1
SCEQLR1092A03.g	GS1_SS_10385_03982	Unknown	uncharacterized	.	.	unknown [Zea mays]		down (-2.6600,-3.8900)	down (-4.3300,-4.1600)	0	2	0	2
SCQGSD2047F01.g	GS1_SS_25860_10456	Unknown	Uncharacterized	.	.	unknown [Zea mays]			up (4.4700,5.2000)	1	0	0	1
SCSBRZ3125B01.g	GS1_SS_18537_11660	Unknown	uncharacterized	.	Cysteine-rich receptor-like protein kinase 28; Cysteine-rich RLK28;	unknown [Zea mays]			up (4.6500,3.7500)	1	0	0	1
SCVPHR1094H06.g	GS1_SS_18536_09986	Unknown	uncharacterized	.	.	.			down (-4.5100,-4.0400)	0	1	0	1
SCRFRZ3055H05.g	GS1_SS_16185_00711	Unknown	Uncharacterized	.	.	unknown [Zea mays]		up (2.5400,2.8500)		1	0	0	1
SCEQRT2029C02.g	GS1_SS_11437_11716	Unknown	uncharacterized	.	.	unknown [Zea mays]		down (-2.6700,-3.3100)		0	1	0	1
SCUTSD2025D08.g	GS1_SS_25514_13378	Unknown	Uncharacterized	.	.	hypothetical protein LOC100191176 [Zea mays] unknown [Zea mays]		down (-2.8400,-3.0200)		0	1	0	1
SCUTLR2008H10.g	GS1_SS_09610_06203	Unknown	uncharacterized	.	.	unknown [Zea mays]			up (3.4200,5.2300)	1	0	0	1
SCRULB1059F07.g	GS1_SS_04611_12552	Unknown	Uncharacterized	Pathogenicity	R-gene	With LRR / NBS-LRR			down (-4.2700,-3.8100)	0	1	0	1
SCEQRT1030C02.g	GS1_SS_12084_12778	Unknown	Uncharacterized	.	.	unknown [Zea mays]			up (3.7800,4.1400)	1	0	0	1
SCEQLR1092B01.g	GS1_SS_10388_08647	Unknown	uncharacterized	.	.	unknown [Zea mays]			down (-5.8500,-8.1300)	0	1	0	1
SCEZLB1007D12.g	GS1_SS_10899_12734	Unknown	uncharacterized	.	.	unknown [Zea mays]		down (-2.4200,-4.2400)	down (-4.0800,-6.2000)	0	2	0	2
SCEQLR1092A06.g	GS1_SS_10386_13595	Unknown	Uncharacterized	.	.	unknown [Zea mays]			down (-3.8600,-4.5700)	0	1	0	1
SCEZAD1081D01.g	GS1_SS_19544_12238	Unknown	uncharacterized	.	.	unknown [Zea mays]			down (-3.0500,-5.0000)	0	1	0	1
SCRFRZ3055F03.g	GS1_SS_16181_09065	Unknown	uncharacterized	.	Chemocyanin precursor (Basic blue protein) (Plantacyanin)	hypothetical protein OsI_021677 [Oryza sativa (indica cultivar-group)]			up (6.7100,3.5600)	1	0	0	1
SCEQLR1091E07.g	GS1_SS_10378_04719	Unknown	Uncharacterized	.	.	Os02g0694800 [Oryza sativa (japonica cultivar-group)] unknown protein [Oryza sativa Japonica Group] unknown protein [Oryza sativa Japonica Group] Os02g0694800 [Oryza sativa (japonica cultivar-group)]			down (-3.3000,-4.1500)	0	1	0	1
SCJFRZ2009D09.g	GS1_SS_14796_14133	Unknown	uncharacterized	.	OTU domain-containing protein 4; HIV-1-induced protein HIN-1	unknown [Zea mays]			down (-3.9300,-5.2500)	0	1	0	1
SCRLRZ3040E11.g	GS1_SS_16235_12911	Unknown	Uncharacterized	.	.	hypothetical protein LOC100191676 [Zea mays] unknown [Zea mays]			down (-3.6500,-4.2000)	0	1	0	1
SCSGLV1012B03.g	GS1_AS_26145_02181	Unknown	Uncharacterized	.	.	.		up (4.3300,6.9900)	up (5.7100,5.8500)	2	0	0	2
SCMCLR1010A05.g	GS1_AS_10755_04665	Unknown	Uncharacterized	.	.	.			down (-3.9400,-3.4400)	0	1	0	1
SCJLST1020E05.g	GS1_SS_17782_07409	Unknown	Uncharacterized	.	.	unknown [Zea mays]			up (3.5500,3.9800)	1	0	0	1
SCQSLB1051H09.g	GS1_SS_19213_02661	Unknown	Uncharacterized	.	.	unknown [Zea mays]		up (6.1400,3.7300)		1	0	0	1
SCVPLB1016D01.g	GS1_SS_19588_16380	Unknown	Uncharacterized	Amino acid metabolism	Cell division inhibitor	Os02g0834700 [Oryza sativa (japonica cultivar-group)] cell division inhibitor-like [Oryza sativa Japonica Group] cell division inhibitor-like [Oryza sativa Japonica Group] Os02g0834700 [Oryza sativa (japonica cultivar-group)]			down (-3.1600,-3.6100)	0	1	0	1
SCCCLR1C11C06.g	GS1_SS_09664_02008	Unknown	uncharacterized	No matches	.	unknown [Zea mays]		down (-2.6200,-5.9100)		0	1	0	1
SCEZRZ1017B05.g	GS1_SS_04550_10066	Unknown	Uncharacterized	.	.	hypothetical protein OsI_006820 [Oryza sativa (indica cultivar-group)]			down (-3.5300,-3.5900)	0	1	0	1
SCRLSD2009D06.g	GS1_AS_25430_03194	Unknown	uncharacterized	No matches	.	.			down (-3.7200,-4.7900)	0	1	0	1
SCSBFL1036C06.g	GS1_SS_20246_00899	Unknown	Uncharacterized	.	Uncharacterized protein C24B11.05	unknown [Zea mays]		up (3.8300,3.2900)	up (4.2100,3.9400)	2	0	0	2
SCCCFL4089H04.g	GS1_AS_22999_08089	Unknown	uncharacterized	.	.	.		down (-2.1400,-3.2700)		0	1	0	1
SCCCLR1024C07.g	GS1_SS_10093_10376	Unknown	uncharacterized	.	.	unknown [Zea mays]		up (1.9600,4.3300)		1	0	0	1
SCCCFL3002D01.g	GS1_SS_19820_09213	Unknown	uncharacterized	.	.	unknown [Zea mays]		down (-2.5200,-3.4500)		0	1	0	1
SCBGLR1120B07.g	GS1_SS_10078_07251	Unknown	uncharacterized	.	.	unknown [Zea mays]			down (-4.0300,-4.2700)	0	1	0	1
SCEZLR1052C01.g	GS1_SS_10435_05099	Unknown	uncharacterized	.	.	.			up (3.1600,3.2900)	1	0	0	1
SCUTLR2015D05.g	GS1_SS_12128_18579	Unknown	Uncharacterized	Protein metabolism	UDP-N-acetylglucosamine--peptide N-acetylglucosaminyltransferase 110 kDa subunit (EC 2.4.1.	hypothetical protein OsI_007133 [Oryza sativa (indica cultivar-group)]			up (4.0900,4.3700)	1	0	0	1
SCCCRZ1001F11.g	GS1_SS_03474_00920	Unknown	Uncharacterized	.	Uncharacterized protein ycf39	hypothetical protein LOC100192036 [Zea mays] unknown [Zea mays]			down (-3.6500,-5.4000)	0	1	0	1
SCRUAD1063A09.g	GS1_SS_00996_13218	Unknown	uncharacterized	.	30S ribosomal protein S21, chloroplastic	unknown [Zea mays]			down (-3.6000,-3.8100)	0	1	0	1
SCEQLB1063D10.g	GS1_SS_09088_14427	Unknown	uncharacterized	.	Protein RUPTURED POLLEN GRAIN 1	unknown [Zea mays]		up (2.0900,3.1600)	up (4.5500,3.5100)	2	0	0	2
SCEQRT1029H04.g	GS1_SS_12121_10055	Unknown	Uncharacterized	Library-specific	By domain	Tryp_alpha_amyl			down (-3.3900,-7.0600)	0	1	0	1
SCSGHR1066C01.g	GS1_SS_08480_19263	Unknown	Uncharacterized	Transporters	Sugar transporters	putative sorbitol transporter [Oryza sativa (japonica			up (3.0500,4.8200)	1	0	0	1
SCJFRZ2025E01.g	GS1_SS_16600_21832	Unknown	uncharacterized	Unknown protein	.	.			up (3.4600,4.8400)	1	0	0	1

SCUTRZ2022E05.g	GS1_SS_15058_11513	Unknown	uncharacterized	.	.	.		down (-4.0600,-5.8900)		0	1	0	1
SCJFST1016C10.g	GS1_SS_17694_09351	Unknown	Uncharacterized	.	Uncharacterized protein At1g08160 OS=Arabidopsis thaliana GN=At1g08160 PE=2 SV=1	Os10g0547200 [Oryza sativa (japonica cultivar-group)] Harpin-induced protein 1 containing protein, expressed [Oryza sativa (japonica cultivar-group)] Os10g0547200 [Oryza sativa (japonica cultivar-group)] hypothetical protein OsJ_031087 [Oryza sativa			up (6.0300,4.7500)	1	0	0	1
SCEZLB1006D09.g	GS1_SS_16619_12462	Unknown	uncharacterized	.	.	Os08g0505200 [Oryza sativa (japonica cultivar-group)] unknown protein [Oryza sativa Japonica Group] unknown protein [Oryza sativa Japonica Group] Os08g0505200 [Oryza sativa (japonica cultivar-group)]		down (-2.0600,-5.0200)	down (-3.8900,-6.8100)	0	2	0	2
SCACLR1132D10.g	GS1_SS_22097_13974	Unknown	uncharacterized	.	.	unknown [Zea mays]			down (-3.7800,-5.2400)	0	1	0	1
SCQSLB1049B08.g	GS1_SS_22106_00067	Unknown	Uncharacterized	.	RNA-dependent RNA polymerase 1 (Protein rdp1)	hypothetical protein OsJ_008023 [Oryza sativa (japonica cultivar-group)]			down (-3.7400,-3.7800)	0	1	0	1
SCSBRZ2021A12.g	GS1_SS_15018_00238	Unknown	Uncharacterized	.	.	hypothetical protein OsI_006929 [Oryza sativa (indica cultivar-group)]		down (-4.1000,-3.9100)		0	1	0	1
SCEQLB1063H01.g	GS1_SS_18982_10957	Unknown	Uncharacterized	.	Non-specific lipid-transfer protein-like protein At5g64080;	unknown [Zea mays]		up (4.5700,4.5700)	up (5.1400,5.2800)	2	0	0	2
SCSFRT2071F11.g	GS1_SS_13907_00263	Unknown	Uncharacterized	.	.	unknown [Zea mays]			down (-3.3700,-3.0900)	0	1	0	1
SCJLRT2052C04.g	GS1_SS_13509_08680	Unknown	Uncharacterized	.	.	unknown [Zea mays]			up (3.4500,3.1800)	1	0	0	1
SCQSFL3038C04.b	GS1_SS_24205_07257	Unknown	Uncharacterized	Receptors	Receptor Ser/Thr kinase-unclassified	caneURLK-42		down (-1.9700,-3.4400)	down (-3.5000,-4.5100)	0	2	0	2
SCQGFL1096G12.g	GS1_SS_21393_10015	Unknown	Uncharacterized	.	Pentatricopeptide repeat-containing protein At1g63080, mitochondrial;	pentatricopeptide repeat protein 5 [Zea mays] pentatricopeptide repeat protein 5 [Zea mays]		down (-2.1700,-3.3800)		0	1	0	1
SCEQLR1050C11.g	GS1_SS_24210_14548	Unknown	Uncharacterized	Protein kinase	.	caneUPK-55			down (-5.0000,-4.1200)	0	1	0	1
SCSBFL1036C06.g	GS1_AS_20246_00900	Unknown	Uncharacterized	.	Uncharacterized protein C24B11.05	unknown [Zea mays]		up (3.0200,4.0400)		1	0	0	1
SCSGLR1025D03.g	GS1_SS_19125_12207	Unknown	uncharacterized	.	.	unknown [Zea mays]		down (-2.2900,-8.0400)		0	1	0	1
SCUTAM2007G01.g	GS1_SS_05369_11779	Unknown	uncharacterized	.	50S ribosomal protein L34, chloroplastic; CL34;	unknown [Zea mays]			down (-5.2000,-5.8600)	0	1	0	1
SCCCSB1002F02.g	GS1_SS_16639_06414	Unknown	Uncharacterized	.	.	unknown [Zea mays]			down (-3.2700,-5.0200)	0	1	0	1
SCRFFL1028F08.g	GS1_SS_20330_05415	Unknown	Uncharacterized	.	.	unknown [Zea mays]			down (-3.6400,-4.1900)	0	1	0	1
SCJFRZ2005B05.g	GS1_SS_22206_04937	Unknown	uncharacterized	.	.	unknown [Sorghum bicolor]		down (-2.3200,-3.7600)	down (-3.4400,-4.7500)	0	2	0	2
SCSFST1064E05.g	GS1_SS_20706_13451	Unknown	uncharacterized	.	Putative flavin-containing monooxygenase 2	unknown [Zea mays]		down (-2.2900,-3.3500)	down (-4.1200,-3.7700)	0	2	0	2
SCSBAD1126C08.g	GS1_SS_21405_10656	Unknown	Uncharacterized	.	.	hypothetical protein SORBIDRAFT_04g007840 [Sorghum bicolor] hypothetical protein SORBIDRAFT_04g007840 [Sorghum bicolor]		up (2.2000,5.8300)		1	0	0	1
SCVPFL1138E03.b	GS1_SS_24140_00113	Unknown	Uncharacterized	.	Glutamate receptor 3.4; AtGLR4; Ligand-gated ion channel 3.4;	hypothetical protein OsI_023330 [Oryza sativa (indica cultivar-group)]			down (-4.3900,-3.1200)	0	1	0	1
SCSBAM1085H07.g	GS1_SS_23824_07279	Unknown	Uncharacterized	.	.	expressed protein [Oryza sativa (japonica cultivar-group)] hypothetical protein OsJ_033073 [Oryza sativa (japonica cultivar-group)]		up (5.4000,4.4600)		1	0	0	1
SCBGAM1093E09.g	GS1_SS_01813_10587	Unknown	uncharacterized	.	.	unknown [Zea mays]			down (-1.8800,-3.2100)	0	1	0	1
SCCCRZ1001F11.g	GS1_AS_03474_00919	Unknown	Uncharacterized	.	Uncharacterized protein ycf39	hypothetical protein LOC100192036 [Zea mays] unknown [Zea mays]			down (-2.4000,-3.0900)	0	1	0	1
SCEQRT2027C06.g	GS1_SS_24536_12695	Unknown	uncharacterized	.	.	unknown [Zea mays] unknown [Zea mays]	down (-1.8300,-1.8600)			0	1	0	1
SCRLAM1009H06.g	GS1_SS_03073_10462	Unknown	Uncharacterized	.	Transcription factor ICE1; Inducer of CBF expression 1; Basic helix-loop-helix protein 116; bHLH 116; AtbHLH116	undeveloped tapetum 1 [Oryza sativa (japonica cultivar-group)]		up (2.4900,3.2600)	up (4.3200,3.3000)	2	0	0	2
SCRLCL6030F09.g	GS1_SS_06848_13857	Unknown	uncharacterized	.	.	.			up (3.2600,3.6400)	1	0	0	1
SCEZRT2019G08.g	GS1_SS_13220_12766	Unknown	Uncharacterized	.	.	unknown [Zea mays]			up (8.1500,5.1000)	1	0	0	1
SCCCLB1002B03.g	GS1_SS_07177_13460	Unknown	Uncharacterized	.	ELMO domain-containing protein DDB_0233912	hypothetical protein OsJ_024650 [Oryza sativa (japonica cultivar-group)]		up (3.1400,2.5800)		1	0	0	1

SCSFRT2069D03.g	GS1_SS_03447_06968	Unknown	uncharacterized	.	.	hypothetical protein OsJ_016009 [Oryza sativa (japonica cultivar-group)]		down (-2.6500,-3.6500)		0	1	0	1
SCRLAM1009F09.g	GS1_SS_03068_07297	Unknown	uncharacterized	.	.	putative gag-pol polyprotein [Zea mays]		up (2.8500,3.0800)		1	0	0	1
SCSFSB1072B06.g	GS1_SS_16938_06182	Unknown	Uncharacterized	.	Protein FLOWERING LOCUS T	ZCN19 protein [Zea mays] ZCN19 [Zea mays] ZCN19 [Zea mays]			up (3.6300,5.7700)	1	0	0	1
SCEPFL3085F03.g	GS1_SS_23488_07450	Unknown	Uncharacterized	.	.	unknown [Zea mays]		down (-2.2500,-4.6700)		0	1	0	1
SCSBFL1101H09.g	GS1_SS_23828_13863	Unknown	Uncharacterized	.	.	unknown [Zea mays]	up (1.6300,1.9100)			1	0	0	1
SCEZAM1079E05.g	GS1_SS_02260_13046	Unknown	uncharacterized	.	.	hypothetical protein OsJ_024166 [Oryza sativa (japonica cultivar-group)]		down (-3.6100,-3.0100)		0	1	0	1
SCBFRT3095H08.g	GS1_SS_25213_07990	Unknown	uncharacterized	.	.	.			down (-3.7900,-3.7800)	0	1	0	1
SCVPLR2012E08.g	GS1_SS_11675_13721	Unknown	uncharacterized	.	.	unknown [Zea mays]		up (7.3500,4.7500)	up (5.3800,6.2500)	2	0	0	2
SCSBFL4066F01.g	GS1_SS_23398_15833	Unknown	uncharacterized	No matches	.	.		up (1.8400,2.5700)	down (-2.8200,-5.0900)	1	1	0	2
SCQGST3154C02.g	GS1_SS_16900_19689	Unknown	Uncharacterized	Transcription Factor	.	NAM		down (-3.0800,-6.9100)	down (-4.2400,-4.6300)	0	2	0	2
SCCCSD2003H02.g	GS1_SS_25185_13860	Unknown	Uncharacterized	.	.	hypothetical protein-like protein [Sorghum bicolor]		down (-2.5300,-3.0400)	down (-3.3800,-3.9300)	0	2	0	2
SCJLRZ1023D09.g	GS1_SS_22864_10620	Unknown	Uncharacterized	.	.	unknown [Zea mays]		down (-2.4900,-5.6600)		0	1	0	1
SCCCCL3005A05.b	GS1_SS_05831_10248	Unknown	uncharacterized	.	.	unknown [Zea mays] unknown [Zea mays]			down (-4.0500,-4.0500)	0	1	0	1
SCQSHR1021B05.g	GS1_SS_16488_12071	Unknown	Uncharacterized	.	Putative protein TPRXL	unknown [Zea mays]			up (5.4900,6.3300)	1	0	0	1
SCRUSB1078F07.g	GS1_SS_22493_11974	Unknown	Uncharacterized	.	.	unknown [Zea mays]		up (4.4700,4.7700)	up (2.9100,4.0300)	2	0	0	2
SCUTFL3071A04.g	GS1_SS_23438_12789	Unknown	uncharacterized	.	Pentatricopeptide repeat-containing protein At1g18900	hypothetical protein LOC100191386 [Zea mays] unknown [Zea mays]		down (-3.4600,-6.7100)		0	1	0	1
SCCCST1008F01.g	GS1_SS_02325_00892	Unknown	Uncharacterized	.	.	unknown [Zea mays]		down (-3.7000,-5.3300)		0	1	0	1
SCVPCL6061B12.g	GS1_SS_15679_05046	Unknown	zinc-binding protein	.	Uncharacterized RING finger protein C2F3.16	unknown [Zea mays]		up (3.8100,5.5200)	up (5.5600,5.2700)	2	0	0	2
SCQGHR1011A04.g	GS1_SS_25114_09855	Unknown		.	O-acyltransferase WSD1 OS=Arabidopsis thaliana GN=WSD1 PE=2 SV=1	hypothetical protein OsJ_003506 [Oryza sativa (japonica cultivar-group)]		up (2.5200,2.9400)		1	0	0	1
SCMCRT2107F12.g	GS1_SS_15529_12941	Unknown		.	.	unknown [Zea mays]			down (-3.8600,-2.9800)	0	1	0	1
SCBFAM2023B06.g	GS1_SS_26061_07590	Unknown		.	.	hypothetical protein LOC100194285 [Zea mays] unknown [Zea mays]			down (-3.8700,-4.6900)	0	1	0	1
SCCCST2004B02.g	GS1_SS_18321_11168	Unknown		.	.	unknown [Zea mays]		down (-3.1800,-3.1400)		0	1	0	1
SCEZLB1012B01.g	GS1_SS_09214_14042	Unknown		.	.	hypothetical protein OsJ_013071 [Oryza sativa (japonica cultivar-group)]		down (-4.1700,-3.8800)	down (-3.0500,-3.9200)	0	2	0	2
SCEQRZ3092B09.g	GS1_SS_15512_10419	Unknown		.	.	hypothetical protein OsJ_028671 [Oryza sativa (japonica cultivar-group)]		down (-5.3800,-7.7300)		0	1	0	1
SCBFSD1038F10.g	GS1_SS_25371_03932	Unknown		.	.	hypothetical protein OsJ_022788 [Oryza sativa (japonica cultivar-group)]			up (2.8200,5.1900)	1	0	0	1
SCSGCL6072C01.g	GS1_SS_06974_02509	Unknown		.	.	.			up (3.3100,5.6000)	1	0	0	1
SCBGFL5077B07.g	GS1_SS_23640_09836	Unknown		.	.	.		down (-2.4700,-4.0600)		0	1	0	1
SCMCAM2083C12.g	GS1_SS_04503_10804	Unknown		.	.	hypothetical protein OsJ_025460 [Oryza sativa (japonica cultivar-group)]			up (5.1000,5.1800)	1	0	0	1
SCJFRZ2015B01.g	GS1_AS_14889_18273	Unknown		No matches	.	.			up (3.5500,4.3400)	1	0	0	1
SCRFAM2071G07.g	GS1_SS_04475_11606	Unknown		.	.	Os02g0617700 [Oryza sativa (japonica cultivar-group)] Os02g0617700 [Oryza sativa (japonica cultivar-group)]		down (-2.1700,-4.1400)		0	1	0	1
SCEQLB1063H04.g	GS1_SS_09094_13039	Unknown		.	.	unknown [Zea mays]		down (-4.5200,-5.6800)		0	1	0	1
SCEPSB1135D05.g	GS1_SS_21061_13273	Unknown		.	.	hypothetical protein LOC100193109 [Zea mays] unknown [Zea mays]		up (6.2300,4.0900)	up (5.0400,4.1800)	2	0	0	2
SCCCLR2C02G11.g	GS1_SS_18193_12935	Unknown		.	.	unknown [Zea mays]			down (-3.7800,-4.1600)	0	1	0	1
SCEQRT2091H09.g	GS1_SS_13053_10192	Unknown		.	.	unknown [Zea mays]			up (4.4300,5.4100)	1	0	0	1
SCCCRT1003B05.g	GS1_SS_11724_09775	Unknown		.	.	.			up (2.8500,5.3500)	1	0	0	1
SCCCRZ2002F11.g	GS1_SS_11680_09616	Unknown		.	.	unknown [Zea mays]		down (-4.2300,-6.3500)		0	1	0	1
SCJFRT2059C10.g	GS1_SS_05003_12892	Unknown		.	.	unknown [Zea mays]			down (-5.9800,-3.6200)	0	1	0	1
SCSBLB2040H08.g	GS1_AS_24760_01971	Unknown		.	.	.		up (3.4400,3.1600)	up (3.8400,4.0100)	2	0	0	2
SCBFSD2034D05.g	GS1_SS_25882_10615	Unknown		.	Uncharacterized protein At3g15000, mitochondrial	unknown [Zea mays]		down (-3.0400,-4.4600)		0	1	0	1
SCCCLR2004H02.g	GS1_SS_16640_12236	Unknown		.	.	hypothetical protein LOC100191649 [Zea mays] unknown [Zea mays]			down (-4.9900,-4.4200)	0	1	0	1
SCCCRZ2C01H11.g	GS1_AS_21744_06635	Unknown		.	.	.			down (-2.9400,-4.6700)	0	1	0	1
SCEZRT2021G08.g	GS1_SS_13248_09439	Unknown		.	.	.			up (6.0800,7.9400)	1	0	0	1
SCEZRZ3099D10.g	GS1_SS_02714_09706	Unknown		.	.	.		down (-3.2800,-5.3400)	up (4.9900,5.3600)	1	1	0	2
SCQSRT2033E01.g	GS1_SS_16543_17072	Vitamin and Cofactor metabolism	Transferase family	Secondary metabolism	putative nicotinate phosphoribosyltransferase	unknown [Zea mays]		up (1.8200,2.6800)		1	0	0	1

Log ratio are shown for replicates
