# Supplementary material for: Identification of sense and antisense transcripts regulated by drought in sugarcane
Source: Plant Mol Biol. 2012 May 19;79(4):461–77. doi: 10.1007/s11103-012-9922-1 (PMC3369129; doi:10.1007/s11103-012-9922-1)
Supplement: Supplementary file 4 — Electronic Suplementary Table 4 – qPCR validation of differentially expressed sense transcripts. (PDF 66 kb) [file 11103_2012_9922_MOESM4_ESM.pdf]

IDENTIFICATION OF SENSE AND ANTISENSE TRANSCRIPTS REGULATED BY DROUGHT IN SUGARCANE

Plant Molecular Biology

Carolina Gimiliani Lembke*[1], Milton Yutaka Nishiyama Jr*[1], Paloma Mieko Sato[1], Rodrigo Fandiño de Andrade[1], Glaucia Mendes Souza[1#]

[1]Laboratório de Transdução de Sinal, Departamento de Bioquímica, Instituto de Química, Universidade de São Paulo.

[#]Corresponding author e-mail address: glmsouza@iq.usp.br

Electronic Supplementary Table 4 – qPCR validation of differentially expressed sense transcripts.

SAS	Functional Category	Structural Category	Gene Name	Oligo info	qPCR primers efficiency	Oligoarray 24h drought	Oligoarray 72h drought	Oligoarray 120h drought	qPCR 24h control	qPCR 24h drought	qPCR 72h control	qPCR 72h drought	qPCR 120h control	qPCR 120h drought
SCACAD1037B06.g	Carbohydrate metabolism	hydratase	Aconitate hydratase 3	GS1_SS_00142_20039	105		up (2.340,2.590)				1.000 (0.159,0.137)	4.103 (0.477,0.427)		
SCEPRT2048D06.g	Carbohydrate metabolism	kinase/phosphatase	Pyrophosphate-fructose 6-phosphate 1-phosphotransferase alpha subunit	GS1_SS_06461_16032	93,4			down (-5.090,-4.520)					1.000 (0.477,0.427)	0.068 (0.025,0.018)
SCUTAM2089E05.g	Carbohydrate metabolism	lyase	Beta-Amylase	GS1_SS_05498_17977	100,5		down (-4.480,-7.430)	down (-5.550,-5.850)			1.000 (0.056,0.053)	0.012 (0.015,0.006)	1.000 (0.368,0.269)	0.086 (0.117,0.049)
SCCCFL4002D04.g	Carbohydrate metabolism	transferase	ADP-glucose pyrophosphorylase small subunit	GS1_SS_19851_18640	106			down (-3.310,-3.700)					1.000 (0,062,0.059)	0.083 (0.028,0.021)
SCEZLB1006F11.g	Carbohydrate metabolism	kinase/phosphatase	Phosphoglycerate kinase	GS1_SS_17085_18093	108,7			down (-4.530,-6.160)					1.000 (0.037,0.036)	0.032 (0.011,0.008)
SCCCCL5003D05.g	RNA metabolism	RNA binding protein	Putative H/ACA ribonucleoprotein complex subunit 1-like protein 1	GS1_SS_08869_19374	105,4		down (-2.860,-4.050)				1.000 (0.103,0.093)	0.473 (0.063,0.056)		
SCCCCL3140E02.g	Signal Transduction	transcription factor	NAC domain-containing protein	GS1_SS_08163_00789	94,9			up (3.840,3.440)					1.000 (0.164,0.141)	18.700 (6.349,4.740)
SCEPLB1041E10.g	Signal Transduction	methyltransferase	Dehydration-responsive family protein	GS1_SS_05313_18090	100,1			down (-4.780,-6.570)					1.000 (0.158,0.137)	0.252 (0.335,0.144)
SCJFRT1059C11.g	Transport	transporter	Aquaporin PIP2-5	GS1_SS_12288_21519	106,4		up (6.040,2.920)				1.000 (0.111,0.100)	0.586 (0.143,0.115)		

The Table indicates gene expression level in Oligoarray and qPCR experiments. In the Oligoarray colum are indicated log ratios from the two biological replicates and in the qPCR colums are indicated the level of expression using control sample as reference and +error and -error values.
