# Supplementary material for: Identification of sense and antisense transcripts regulated by drought in sugarcane
Source: Plant Mol Biol. 2012 May 19;79(4):461–77. doi: 10.1007/s11103-012-9922-1 (PMC3369129; doi:10.1007/s11103-012-9922-1)
Supplement: Supplementary file 5 — Electronic Suplementary Table 5 - qPCR validation of differentially expressed sense and antisense transcripts. (PDF 67 kb) [file 11103_2012_9922_MOESM5_ESM.pdf]

IDENTIFICATION OF SENSE AND ANTISENSE TRANSCRIPTS REGULATED BY DROUGHT IN SUGARCANE

Plant Molecular Biology

Carolina Gimiliani Lembke*[1], Milton Yutaka Nishiyama Jr*[1], Paloma Mieko Sato[1], Rodrigo Fandiño de Andrade[1], Glaucia Mendes Souza[1#]

[1]Laboratório de Transdução de Sinal, Departamento de Bioquímica, Instituto de Química, Universidade de São Paulo.

[#]Corresponding author e-mail address: glmsouza@iq.usp.br

Electronic Supplementary Table 5 – qPCR validation of differentially expressed sense and antisense transcripts.

SAS	Functional Category	Structural Category	Gene Name	Oligo info	qPCR primers efficiency	Oligoarray 24h drought	Oligoarray 72h drought	Oligoarray 120h drought	qPCR 24h control	qPCR 24h drought	qPCR 72h control	qPCR 72h drought	qPCR 120h control	qPCR 120h drought
SCBGST3105A06.g	Carbohydrate metabolism	kinase/phosphatase	Fructose-1,6-bisphosphatase I	GS1_SS_18508_15468	96,5			down (-3.240,-5.140)					1.000 (0.172,0.147)	0.187 (0.062,0.047)
				GS1_AS_18508_15469				down (-2.730,-3.120)					1.000 (0.425,0.298)	0.091 (0.017,0.015)
SCJFLR1017E09.g	Carbohydrate metabolism	synthase	Alpha galactosidase 1	GS1_SS_08724_06083	102,2		up (4.670,2.360)				1.000 (0.266,0.210)	84.253 (7.543,6.922)		
				GS1_AS_08724_06084				up (3.960,4.490)					1.000 (0.342,0.255)	16.951 (5.069,3.902)
SCJFRZ2014D06.g	DNA metabolism	transcription factor	ATAF1 Protein	GS1_SS_12278_15377	95,3		up (4.230,3.910)	up (5.120,3.640)			1.000 (0.343,0.255)	38.098 (2.934,2.723)	1.000 (0.110,0.099)	8.614 (2.322,1.829)
				GS1_AS_12278_15378			up (3.750,3.020)				1.000 (0.350,0.259)	51.032 (8.940,7.607)		
SCCCLR1075B06.g	Light Harvesting	others	Photosystem II 10kDa polypeptide	GS1_SS_10234_14797	93,1		up (8.230,5.400)				1.000 (0.095,0.087)	5556.651 (238.188,228.398)		
				GS1_AS_10234_14796			up (3.150,3.750)				1.000 (0.444,0.307)	9068.616 (1165.976,1033.142)		
SCQGLR1085G01.g	Light Harvesting	photosystem I subunit	Photosystem I reaction center subunit V	GS1_SS_25636_20097	93,1			down (-5.240,-6.890)					1.000 (0.052,0.050)	0.067 (0.018,0.014)
				GS1_AS_25636_20096				down (-4.240,-3.990)					1.000 (0.334,0.251)	0.027 (0.004,0.004)
SCACLR1057H07.g	Phorphyrin and chlorophyll metabolism	others	Magnesium chelatase subunit ChlI	GS1_SS_04477_20876	96,1		down (-3.920,-7.360)	down (-7.620,-8.400)			1.000 (0.121,0.108)	0.002 (0.001,0.001)	1.000 (0.190,0.159)	0.010 (0.009,0.005)
				GS1_AS_04477_20877			down (-1.320,-3.280)				1.000 (0.059,0.055)	0.006 (0.001,0.001)		
SCJLRT1016G06.g	RNA metabolism	hydrolase	Ribonuclease	GS1_SS_12487_19039	98,4		up (5.650,3.540)				1.000 (0.367,0.269)	92.518 (19.005, 15.766)		
				GS1_AS_12487_19040			up (2.990,3.220)				1.000 (0.514,0.339)	194.460 (46.272,37.377)		
SCBFLR1026B07.g	RNA metabolism	RNA binding protein	Nucleolar protein Nop56	GS1_SS_24393_15654	110,7		down (-2.670,-4.480)				1.000 (0.134,0.118)	0.227 (0.056,0.044)		
				GS1_AS_24393_15653			down (-2.830,-3.450)				1.000 (0.868,1.153)	0.302 (0.129,0.090)		

The Table indicates gene expression level in Oligoarray and qPCR experiments. In the Oligoarray colum are indicated log ratios from the two biological replicates and in the qPCR colums are indicated the level of expression using control sample as reference and +error and -error values.
