# Supplementary material for: Identification of sense and antisense transcripts regulated by drought in sugarcane
Source: Plant Mol Biol. 2012 May 19;79(4):461–77. doi: 10.1007/s11103-012-9922-1 (PMC3369129; doi:10.1007/s11103-012-9922-1)
Supplement: Supplementary file 6 — Electronic Supplementary Table 6 – Primer sequences for qPCR validation of differentially expressed transcripts. (PDF 7 kb) [file 11103_2012_9922_MOESM6_ESM.pdf]

IDENTIFICATION OF SENSE AND ANTISENSE TRANSCRIPTS REGULATED BY DROUGHT IN SUGARCANE

Plant Molecular Biology

Carolina Gimiliani Lembke*[1], Milton Yutaka Nishiyama Jr*[1], Paloma Mieko Sato[1], Rodrigo Fandiño de Andrade[1], Glaucia Mendes So

[1]Laboratório de Transdução de Sinal, Departamento de Bioquímica, Instituto de Química, Universidade de São Paulo.

[#]Corresponding author e-mail address: glmsouza@iq.usp.br

Supplementary Table 6 – Primer sequences for qPCR validation of differentially expressed transcripts.

SAS	Forward primer name	Forward primer sequence	Reverse primer name	Reverse primer sequence
SCACAD1037B06.g	Fw 1037B06RT	GCATCCTCACCAGGCTTGAA	Rv 1037B06RT	GAGGATCCACCGCAGCAA
SCEPRT2048D06.g	Fw 2048D06RT	GGAAGGCGTGGCTGATGA	Rv 2048D06RT	GTACGGTGATGCTGCAATCG
SCUTAM2089E05.g	Fw 2089E05RT	TACGACTGGAGCGCCTACAA	Rv 2089E05RT	TGATGGCCTGCAGCTTGAG
SCCCFL4002D04.g	Fw 4002D04RT	CGAGGTGCTGATCATTGTCAA	Rv 4002D04RT	AGGATGCTTTACTCCCTAGTGGAA
SCEZLB1006F11.g	Fw 1006F11RT	GGAATTGCAGATGCTGGAACA	Rv 1006F11RT	TGTTGTCATTGCTGATAAGTTTGC
SCCCCL5003D05.g	Fw 5003D05RT	TTAGAGGGCGAGGCAGGTT	Rv 5003D05RT	GGCAGCTATGATGCTTCAAGAA
SCCCCL3140E02.g	Fw 3140E02RT	GGTAATTACAGCGGTTAGGCAACT	Rv 3140E02RT	CATGTTGATTGATTATTACTGTCTGATTG
SCEPLB1041E10.g	Fw 1041E10RT	TGCTGTTCTTTTTGGACGGTACT	Rv 1041E10RT	TTTCCACCCCTGTTACACCTTAA
SCJFRT1059C11.g	Fw 1059C11RT	GCGTCGTCGGTTGCTTCT	Rv 1059C11RT	AATACAGACGCAAACTGATCATAAGG
SCBGST3105A06.g	Fw 3105A06RT	CGGGTCCAAGAACGAGATCA	Rv 3105A06RT	AGCCTGGACGAGCCGTAGA
SCJFLR1017E09.g	Fw1017E09RT	CGCGGCTGAGCCATTCT	Rv1017E09RT	CCCCATCTCTCGTGCACAT
SCJFRZ2014D06.g	Fw 2014D06RT	AAGAACCTCGCCTGTAGGAAATC	Rv 2014D06RT	GCTCTGATCCAATCTTTCCAATAATC
SCCCLR1075B06.g	Fw 1075B06RT	TCCAAACATCCCAGCCTTTC	Rv 1075B06RT	TGCATCAACACCATCCTTCAA
SCQGLR1085G01.g	Fw 1085G01RT	GGATACGACCCCAACTTCTTCTG	Rv 1085G11RT	AACGGAGTAGAATACAAGCTCAAGAAC
SCACLR1057H07.g	Fw 1057H07RT	CTACTGTCATTCCAAACTGCTTGAG	Rv 1057H07RT	AGCAAACCCGAGTCAATGGAT
SCJLRT1016G06.g	Fw 1016G06RT	CGAGTTCCCGGCGTTCTA	Rv 1016G06RT	TTGCAGAGAATTGGAACAACTGA
SCBFLR1026B07.g	Fw 1026B07RT	AGAGGATGCACAGGAGGAGAAC	Rv 1026B07RT	TGGGAGTCTCGGTCTCTTCAG
